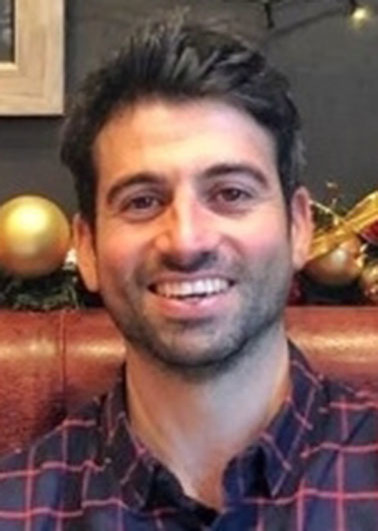# New Trends and Future Opportunities in the Enzymatic Formation of C−C, C−N, and C−O bonds

**DOI:** 10.1002/cbic.202100464

**Published:** 2021-11-24

**Authors:** Jack J. Sangster, James R. Marshall, Nicholas J. Turner, Juan Mangas‐Sanchez

**Affiliations:** ^1^ Institute of Chemical Synthesis and Homogeneous Catalysis Spanish National Research Council (CSIC) Pedro Cerbuna 12 50009 Zaragoza Spain; ^2^ ARAID Foundation Zaragoza Spain; ^3^ Department of Chemistry Manchester Institute of Biotechnology University of Manchester 131 Princess Street Manchester M1 7DN UK

## Abstract

Organic chemistry provides society with fundamental products we use daily. Concerns about the impact that the chemical industry has over the environment is propelling major changes in the way we manufacture chemicals. Biocatalysis offers an alternative to other synthetic approaches as it employs enzymes, Nature's catalysts, to carry out chemical transformations. Enzymes are biodegradable, come from renewable sources, operate under mild reaction conditions, and display high selectivities in the processes they catalyse. As a highly multidisciplinary field, biocatalysis benefits from advances in different areas, and developments in the fields of molecular biology, bioinformatics, and chemical engineering have accelerated the extension of the range of available transformations (E. L. Bell *et al*., *Nat. Rev. Meth. Prim*. **2021**, *1*, 1–21). Recently, we surveyed advances in the expansion of the scope of biocatalysis via enzyme discovery and protein engineering (J. R. Marshall *et al*., *Tetrahedron* **2021**, *82*, 131926). Herein, we focus on novel enzymes currently available to the broad synthetic community for the construction of new C−C, C−N and C−O bonds, with the purpose of providing the non‐specialist with new and alternative tools for chiral and sustainable chemical synthesis.

## Graphical abstract

**Recent research** on the biocatalytic formation of new C−C, C−N, and C−O is surveyed in this review to provide an overview of the current state‐of‐the‐art in the field as well as future perspectives.

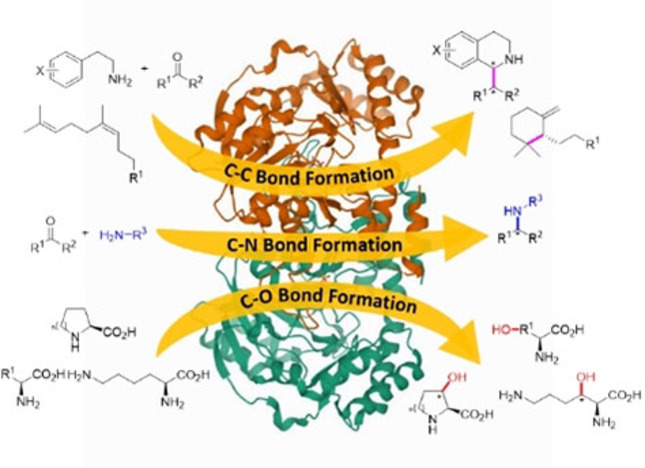

## 1 Emerging Enzymes for Biocatalytic C−C Bond Formation

The formation of carbon‐carbon (C−C) bonds is arguably the most fundamental transformation underpinning organic synthesis; it enables the coupling of small building blocks to form the framework of a multitude of organic molecules including API's, natural products, and agrochemicals.[^1^, ^2^] Transition metal based catalysts have demonstrated to be excellent tools for the construction of new C−C bonds in an asymmetric fashion, although such transformations often require expensive metal catalysts, organic solvents and harsh reaction.[^1^, ^3^, ^4^] By contrast, enzymes are highly selective catalysts which operate in water at ambient temperatures, and therefore offer a more sustainable approach to asymmetric C−C bond formation.^[5]^ Even though enzymatic catalysis is far from reaching the plethora of transformations available through small molecule chemical catalysts, major advancements in metagenomic sequencing, recombinant DNA technology, and directed evolution since the turn of the millennium provide a promising platform to the adoption of biocatalysis in this area.[^5^, ^6^, ^7^] However, the toolbox of biocatalysts currently available to synthetic chemists for C−C bond formation is still limited by the narrow range of newly discovered enzyme classes, thus hindering their widespread application. Previously, biocatalytic C−C bond formation catalysed by aldolases, hydroxynitrile lyases, transketolases and ThDP‐dependent carboligases have been extensively employed in organic synthesis and this research is the subject of several reviews.[^1^, ^3^, ^8^, ^9^] To address the issue of the narrow window of enzymatic transformations mentioned above, the discovery of enzymes capable of catalysing novel C−C bond forming reactions is essential for the broader utilisation of biocatalysis by synthetic chemists. Over the past five years, biocatalytic approaches to Friedel‐Crafts alkylation/acylation, trifluoromethylation as well as highly engineered non‐natural carbene transferases have been outlined.[^6^, ^10^] Continued research interest in biocatalytic C−C bond formation has resulted in more novel enzymes and methodologies for carbon bond formation being reported in literature. This review examines these novel biocatalytic transformations, which include cyclisations, and the Diels‐Alder and Pictet‐Spengler reactions.

### 1.1 Biocatalytic cyclisations (cyclases)

Both nature, and synthetic chemists, have widely employed cyclisation reactions, enabling the generation of multi‐functional products from simple building blocks. These reactions can occur either through inter‐ or intramolecular cyclisation and involve the formation of a single C−C bond or multiple C−C bonds via a cascade process.

#### 1.1.1 Terpene cyclases

**Scheme 1:**
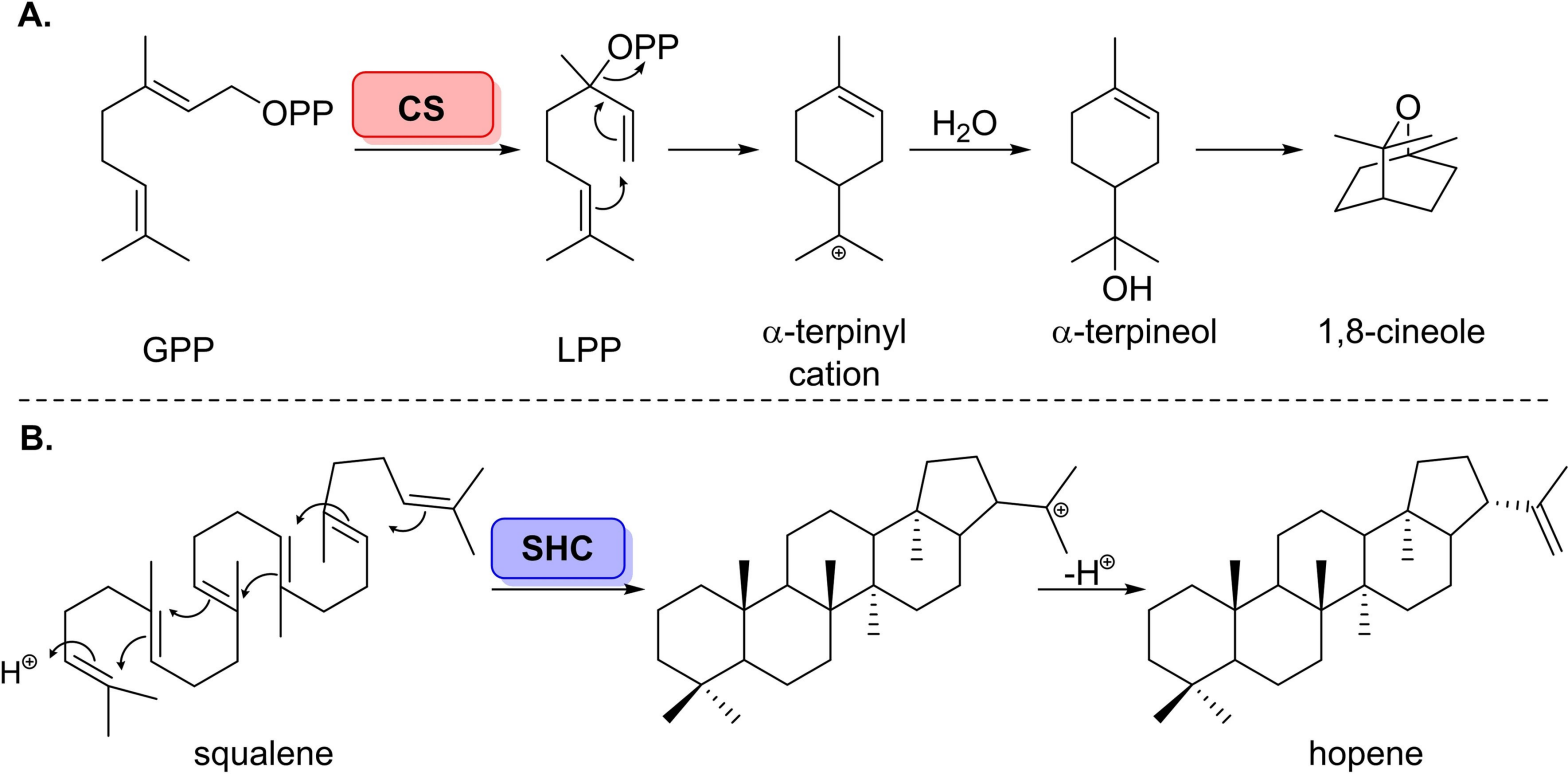
(A) The mechanism for cyclisation of GPP catalysed by class I terpene cyclase, cineole synthase (CS) to afford 1,8‐cineole. (B) Mechanism for the class II terpene cyclase, squalene‐hopene cyclase (SHC), showing the proton‐induced cyclisation of squalene.

Terpenoids are the most structurally diverse family of natural products, comprising of over 80 000 compounds which have uses in flavourings, fragrances, and pigments.[^11^, ^12^, ^13^] Impressively, the diverse family of terpenoids derive from only two isomeric C_5_ methyl branched precursors.^[14]^ Molecular complexity in these biosynthetic molecules is generated through cascade cyclisation of unsaturated terpene scaffolds to cyclic terpenoids, wherein multiple fused rings are formed stereoselectively in a single step.[^11^, ^15^] The outcome of these cyclisations are controlled by the interactions of the substrate with a hydrophobic pocket in the active site of the enzyme.[^15^, ^18^] There are two main enzyme classes which have evolved to catalyse these transformations; class I and II terpene cyclases (Scheme 1).^[12]^ Class I terpene cyclases are defined by their catalytic mechanism, whereby the abstraction of diphosphate instigates the cyclisation which proceeds via nucleophilic addition of the C−C double bonds onto the carbocation intermediates, and is terminated either by deprotonation or addition of water.^[11]^ On the other hand, the mode of catalysis exhibited by class II terpene cyclases is mechanistically dissimilar to that of class I enzymes, resembling Brønsted acid catalysis. The mechanism for class II terpene cyclases relies on protonation, or epoxidation of the terminal isoprenoid C−C double bond prior to cyclisation.^[16]^

#### 1.1.2 Class I terpene cyclases

Class I terpene cyclases are metal‐dependent enzymes which catalyse the cyclisation of isoprenoid diphosphate substrates to products containing one or more fused rings.[^12^, ^15^] During catalysis, the oligo‐prenyl precursor binds to a cluster of three Mg^2+^ ions resulting in loss of the substrate diphosphate group to form a carbocation‐inorganic diphosphate (PP_i_) pair. It is this carbocation which then undergoes cyclisation.^[17]^ The family of class I cyclases can be split into three sub‐classes which are; monoterpene e. g. (−)‐limonene cyclase, sesquiterpene e. g. α‐bisabolol cyclases and diterpene cyclases e. g. taxadiene cyclases.[^18^, ^19^, ^20^] These three sub‐families all share a very similar catalytic mechanism, but can be differentiated by the structure of the native substrate and hence, the outcome of the cyclisation reaction.^[12]^ The majority of research carried out on class I terpene cyclases has focused on sesquiterpene, while examples on mono‐ and diterpene cyclases are lacking.[^11^, ^21^]

Marketed under the brand name Taxol, the diterpene paclitaxel is one of the most successful and highest grossing chemotherapeutics.^[22]^ During a natural product screening campaign carried out in the 1960s by the National Cancer Institute (NCI) and the U.S department for agriculture, paclitaxel was first isolated from the bark of the pacific yew tree (*Taxus bervifolia*) and its cytotoxicity established.^[23]^ Currently, the drug is prescribed in generic form for the treatment of ovarian, breast, non‐small‐cell lung carcinoma and Kaposi's sarcoma.[^24^, ^25^, ^26^, ^27^] The industrial production of paclitaxel remains problematic, as the yield for natural harvesting can be as low as 0.001–0.05 %.^[28]^ In order to overcome this issue, around thirty research groups were working towards a total synthesis of this diterpene during the 1990s. Nicolaou and co‐workers were the first group to publish their approach, however the synthesis required over 26 chemical steps resulting in a very low overall yield of 0.078 %.^[29]^ Since this initial publication, only nine examples of the total synthesis of paclitaxel have been reported, all requiring numerous chemical manipulations and hindering their commercial viability.[^30^, ^31^, ^32^, ^33^, ^34^, ^35^, ^36^, ^37^] Around the same time, Witherup *et al*. showed that large quantities of the paclitaxel biosynthetic intermediate, 10‐deacetylbaccatin III (baccatin III), could be isolated from European yew tree *Taxus baccata*, enabling a concise semisynthesis of paclitaxel.^[38]^ This approach was further improved by Holton and co‐workers who outlined a 4 step semisynthesis with an overall yield of 4–5 %, which was subsequently patented and sold to Bristol‐Myers Squibb (BMS).^[39]^ Although this approach has improved upon the previous methods and is used for the commercial production of paclitaxel, it is still ecologically demanding and requires toxic chemical reagents. This has led to a great deal of interest in exploiting synthetic biology and biocatalysis for the *in vivo* or *in vitro* preparation of paclitaxel and its analogues.

**Scheme 2:**
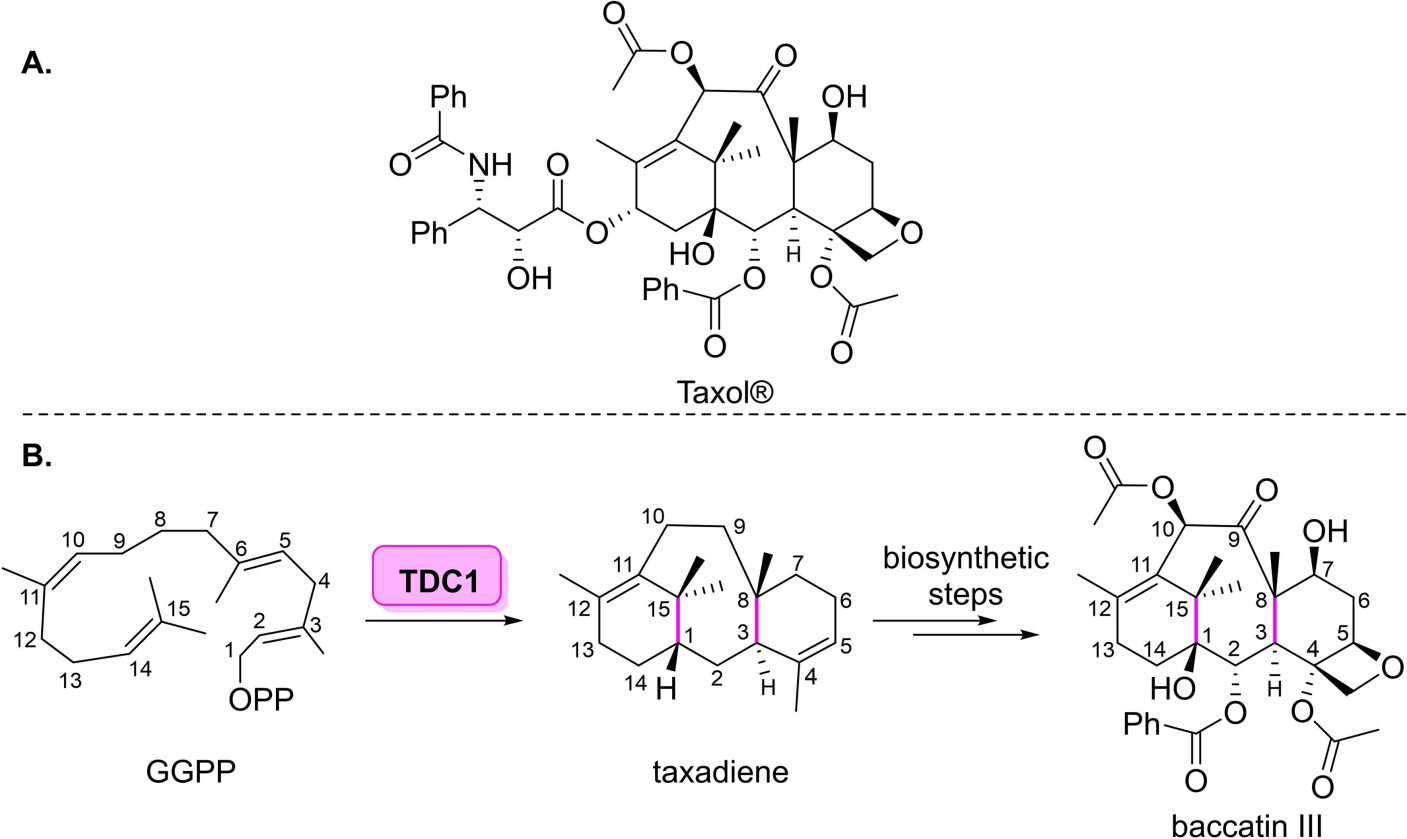
(A) Structure of paclitaxel (Taxol®) (B) Polycyclisation of GGPP catalysed by TDC1 in the biosynthesis of paclitaxel.

The biosynthesis of this tetracyclic diterpene is thought to contain nineteen steps, starting from isoprenyl diphosphate (IPP) and dimethylallyl diphosphate (DMAPP).^[28]^ The first committed step in the pathway involves the cyclisation of geranylgeranyl diphosphate (GGPP) to tax‐4(5),11(12)‐diene, which is catalysed by the diterpene cyclase, taxadiene cyclase (TDC1) from *Taxus bervifolia* (Scheme 2).[^20^, ^40^] A biosynthetic route towards paclitaxel has the potential to overcome the ecological issues and the dependence on toxic reagents required in the semisynthetic approach. Metabolic engineering can be used to manipulate the biosynthesis of paclitaxel in bacterial or yeast has potential over plant‐cell fermentation methods. Although the cultivation of paclitaxel cell lines in the lab has shown to be commercially viable, this approach is not able to easily access paclitaxel analogues, hindering its applicability.^[41]^ Currently, taxadiene cyclase has been exploited for taxadiene overproduction in *E. coli*, yeasts and *Artemisia annua L*, paving the way for a full biosynthetic approach to paclitacxel.[^42^, ^43^, ^44^]

**Scheme 3:**
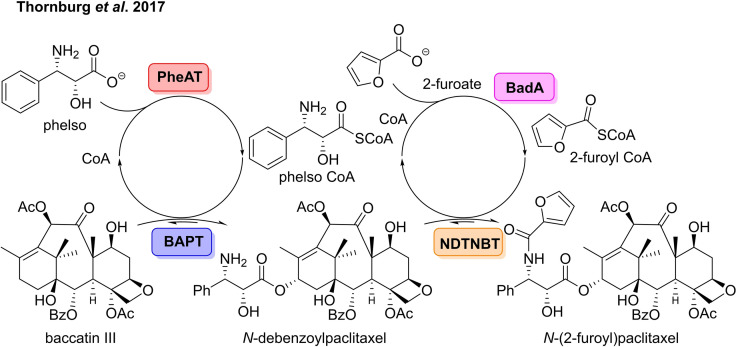
Four enzyme cascade for the preparation of paclitaxel analogues starting from commercially available baccatin III.

The metaphorical cherry on top of the cake for the preparation of paxitaxel would involve a streamlined biocatalytic cascade to prepare the drug and its analogues from easily accessible starting materials, reducing side‐reactions and easing subsequent isolation. Over the past twenty years, Walker and co‐workers have outlined a plethora of enzymes involved in paclitaxel biosynthesis.[^45^, ^46^, ^47^, ^48^] In 2017 the group was able to use their previous knowledge to build a four enzyme cascade towards a paclitaxel analogue.^[49]^ The cascade involved a benzoate CoA ligase (BadA), a modified nonribosomal peptide synthase (PheAT), a phenylpropanoyltransferase (BAPT), and a benzoyltransferase (NDTNBT) to furnish a paclitaxel analogue from commercially available precursor, baccatin III (Scheme 3).[^48^, ^49^, ^50^] After optimisation of the multi‐enzyme cascade, the group was able to prepare 230 ng of *N*‐(2‐furoyl)paclitaxel, which was show to be more cytotoxic than paclitaxel against certain cell lines. The group also demonstrated that the cascade could be used to prepare the intermediate, *N*‐debenzoylpaclitaxel, with 20 times greater yield than that for *N*‐(2‐furoyl)paclitaxel. The group have also shown that BadA can convert aroyl acids to aroyl CoA thioesters, generating an *N*‐aroylpaclitaxel analogue.^[51]^ By recycling the free CoA generated in the cascade, it was possible to furnish the product in higher yield than in assays carried out without CoA recycling.

**Scheme 4:**
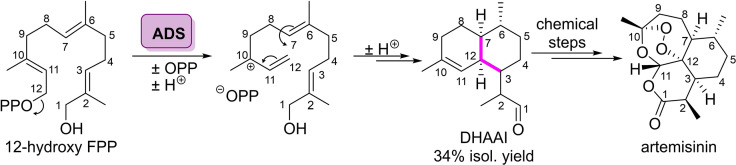
Formal synthesis of artemisin via biocatalytic cyclisation catalysed by the amorphadiene synthase (ADS) from *Artemisia annua*.

Sesquiterpene cyclases (STCs) catalyse the cyclisation of farnesyl pyrophosphate (FPP) to cyclic sesquiterpenes.^[52]^ This cyclisation can proceed through four possible C−C bond forming trajectories depending on which C−C double bond reacts initially.[^12^, ^53^] Thus, the molecular complexity generated in sesquiterpene cyclisation is far larger than those generated through monoterpene cyclisation. Recent work has focused on the transformation of non‐natural substrates catalysed by known STCs, allowing access to novel terpene architectures. Allemann and co‐workers used the amorphadiene synthase (ADS) from *Artemisia annua* to cyclise 12‐hydroxy FPP to afford the artemisinin precursor dihydroartemisinic aldehyde (DHAAI) (Scheme 4).[^54^, ^55^] Artemisinin is a potent anti‐malarial drug which can be synthesised from DHAAI in four chemical steps.[^56^, ^57^] The group scaled up the synthesis of DHAAI, incubating 12‐hydroxy FPP with purified ADS for 48 hours, obtaining DHAAI in 34 % yield. They further improved on this work, affording DHAAI in an excellent yield of 90 %, using the natural substrate under flow conditions.^[58]^ These initial studies with chemically modified FPP substrates paved the way for further development in this area.

**Scheme 5:**
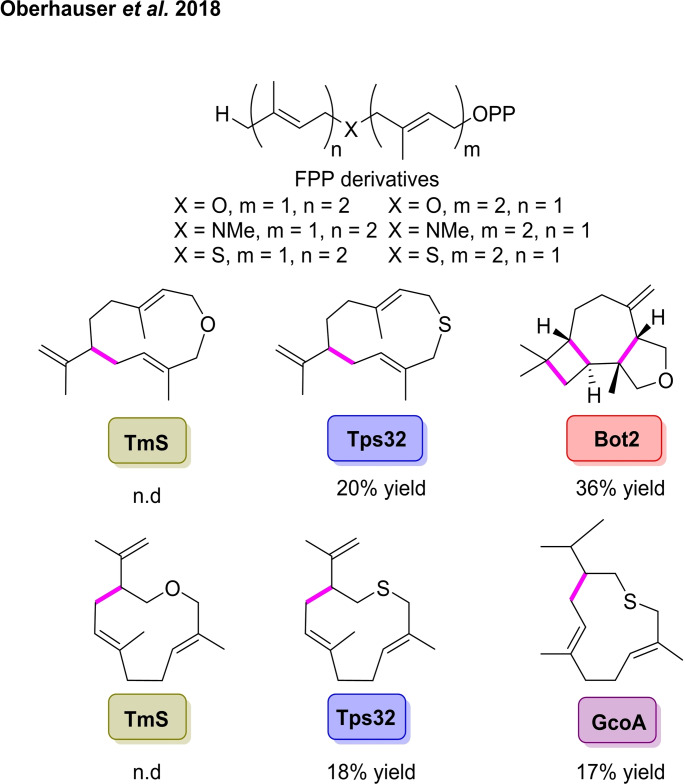
Mono‐ and polycyclisation of non‐natural heteroatom functionalised FPP derivatives catalysed by sesquiterpene cyclases (STCs).

This work was built on by Kirschning *et al*. who reported the synthesis of novel non‐natural terpenoids through the cyclisation of heteroatom‐modified FPPs with a number of STCs (Scheme 5).^[59]^ The cyclases used in their investigation were: patchoulol synthase (Pts) from *Pogostemon cablin*, viridiforene synthase (Tps32) from *Solanum lycopersicum*, vetispiradiene synthase (Hvs1) from *Hyoscamus muticus*, caryolan‐1‐ol synthase (GcoA1) from *Streptomyces griseus*, (+)‐T‐muurolol synthase (TmS) from *Roseifexus castenholzii*, pentalenene synthase (PenA) from *Streptomyces exfoliatus*, presilphiperfolan‐8b‐ol synthase (Bot2) from *Botrytis cinerea*, and cubebol synthase (Cop4) from *Corinus cinereus*.[^60^, ^61^, ^62^, ^63^, ^64^, ^65^, ^66^, ^67^, ^68^] The group initially found that the two amine‐containing FPP derivatives were not converted by any of the enzymes. The cyclisation of the oxo‐ and thio‐substrates was successfully achieved by a number of the STCs, generating a series of novel oxygen‐ and sulphur‐containing macrocycles through a single C−C bond forming step. The most unusual result observed was the formation of the tricyclic product in 36 % yield, formed from the oxo‐substituted FPP catalysed by Bot2. This tricyclic compound could have potential use in fragrances, showing olfactory properties which include ethereal, peppery and camphoric.^[69]^

**Scheme 6:**
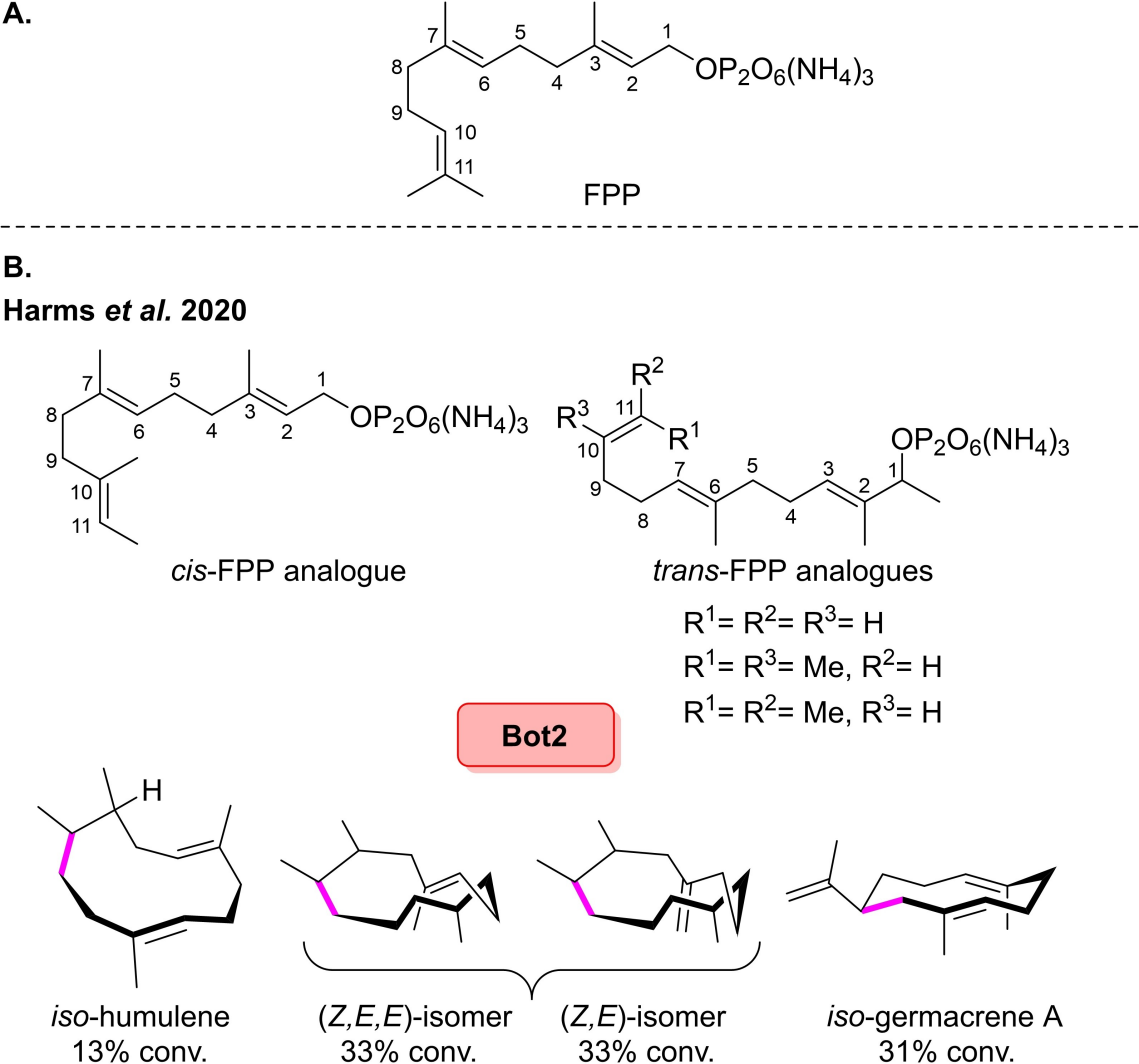
(A) Structure of farnesyl pyrophosphate (FPP). (B) *Bc*BOT2 mediated cyclisation of novel FPP analogues.

Then in 2020, Kirschning and co‐workers were also able to access other novel sesquiterpene backbones using non‐natural FPPs.^[70]^ The previously reported cyclase, *Bot*2, was chosen as the biocatalysts for this investigation.^[67]^ The group first prepared the *cis*‐FPP analogue, where one of the C11 methyl groups in FPP was shifted onto C10 in a *cis*‐configuration (Scheme 6A). For the *cis*‐FPP, enzymatic cyclisation afforded a single major product, *iso*‐humulene in 13 % conversion (Scheme 6B). *Trans*‐FPP analogues were synthesised, wherein the methyl groups on C3 and C7 of FPP were moved to C2 and C6, respectively. Also, one methyl group on C11 in FPP was transferred to C10 in a *trans‐*configuration. Bot2 catalysed cyclisation of the *trans*‐FPP derivative where R^1^=R^2^=Me R^3^=H afforded the cyclised product, *iso*‐germacrene A, in 33 % conversion. However, the *trans*‐FPP derivatives where R^1^=R^3^=Me R^2^=H was transformed into two main isomeric macrocyclic compounds, (*Z*,*X*,*X*)‐isomer and (*Z*,*E*)‐isomer in 33 % conversion for each. This is the first reported example of Bot2 converting a non‐natural FPP derivative into a 10‐membered macrocycle, although it is postulated that the substrate initially undergoes C1 to C11 cyclisation prior to Wagner‐Meerwein rearrangement. The novel terpenoids which were afforded through these biotransformations showed a range of olfactoric properties, which could also show potential in the flavours and perfume industries.[^67^, ^69^]

**Scheme 7:**
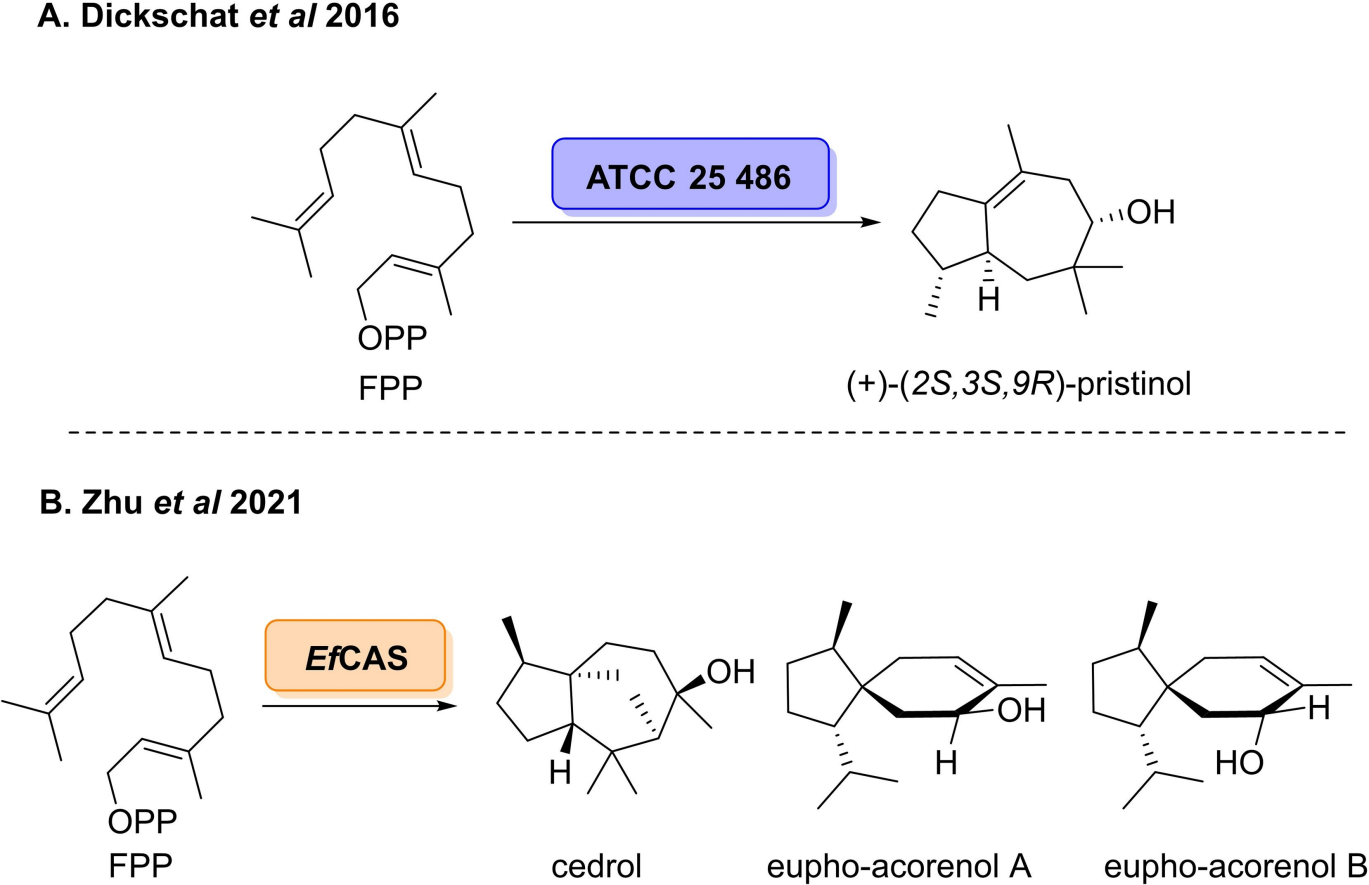
Novel STCs to access **5**–**8** bicyclic sesquiterpene alcohols.

An alternative approach to access novel terpene sesquiterpene scaffolds is to use metagenomics to elucidate STCs which catalyse novel chemistries, enabling access to new molecular topologies. In 2016, Dickschat *et al*. reported the characterisation of a bacterial STC from *Streptomyces pristinaespiralis, ATCC 25 486*, affording a novel **5**–**8** bicyclic carbon skeleton.^[71]^ The enzyme was able to convert the natural substrate FPP into a single sesquiterpene alcohol, which was isolated in 33 % yield (Scheme 7A). Using ^1^H NMR experiments and single‐wavelength anomalous dispersion (SAD) X‐ray crystallography the group was able to determine the structure of the novel **5**–**8** bicyclic product, which was named (*2S,3S,9R*)‐pristinol. The 8‐mebered ring structure generated by *ATCC 25 486* is rarely found in sesquiterpenes, exemplifying how enzyme discovery can be used to find enzymes which can catalyse novel cyclisations. Plant species which have been used throughout history as traditional medicines can also be exploited for discovering new enzymes.^[72]^ In 2021, Zi and co‐workers reported the discovery of a novel STC which could afford a similar 5,8‐bicyclic core.^[73]^ The enzyme was discovered from *Euphorbia ficheriana* steud, the root of which is used in traditional Chinese medicine for the treatment of edema and tuberculosis.[^72^, ^74^] The newly discovered enzyme, *Ef*CAS, efficiently catalysed the cyclisation of FPP to cedrol and two tricho‐acorenol stereoisomers; eupho‐acorenols A and B which were characterised by NMR spectroscopy (Scheme 7B). Both products are constituents of plant volatile oils, which have application in flavour, perfume and cosmetics industries. While tricho‐acorenol is a metabolite of *Trichoderma* fungi, which has applications in biocontrol and biostimulant agents.[^75^, ^76^]

**Scheme 8:**
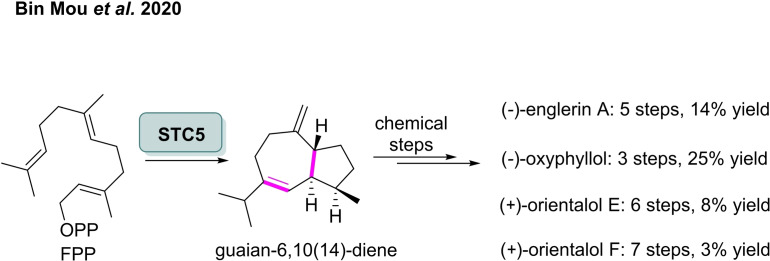
Highly stereoselective enzymatic synthesis of guaian‐6,10(14)‐diene precursor prior to chemical transformation to biologically active epoxy‐guaiane sesquiterpenes.

It has also been shown that enzymatic cyclisation can be exploited to produce highly functionalised sesquiterpene scaffolds, which can then be diversified through chemical modification.^[77]^ Through metabolic engineering of the isoprenoid pathway in *E. coli*, Mou and co‐workers were able to produce the molecular scaffold, guaian‐6,10(14)‐diene, with a high degree of stereo‐ and regio‐selectivity exploiting the cyclases, STC5.^[78]^ This enzyme catalyses the cyclisation of the natural substrate FPP to afford a guaian‐6,10(14)‐diene intermediate, which they then converted chemically into a number of bioactive compounds (Scheme 8).^[79]^ A total of six chemical and enzymatic steps were employed to convert FPP into (−)‐englerin A with an overall yield of 14 %. They also reported the synthesis of (−)‐oxyphyllol, (+)‐orientalol E and (+)‐oriental F, achieving overall yields of 25 %, 8 % and 3 %, respectively. The evaluation of the biological activities of these compounds is currently under investigation.

#### 1.1.3 Class II terpene cyclases

Triterpene cyclases, such as oxidosqualene (OSC) and squalene‐hopene (SHC) cyclases, belong to the family of class II terpene cyclases.[^80^, ^81^] The most comprehensively studied class II triterpene cyclase is the SHC from *Alicyclobacillus acidocaldarius*, *Aac*SHC.[^82^, ^83^, ^84^] It converts the natural substrate squalene to the pentacyclic products hopene and hopanol.^[11]^ SHC's have also been shown to accept terpene analogues, which has previously been outlined in several reviews.[^1^, ^80^] On the other hand, oxidosqualene cyclases catalyse the cyclisation of squalene epoxide to form lanosterol, which is the first step in cholesterol biosynthesis.^[12]^ Current trends in this area has focused on using substrate engineering to generate novel terpenoids, genetic engineering of SHC to manipulate the outcome of the cyclisation and also using metagenomics to discovery novel cyclases which catalyse new reactions.

**Scheme 9:**
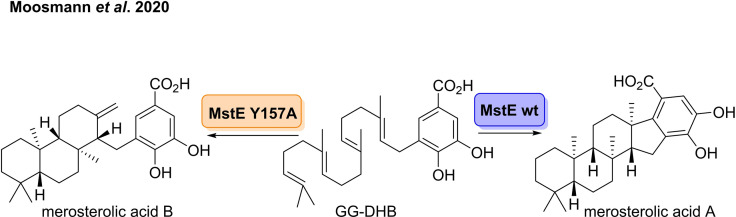
A novel terpene cyclase catalysed polycyclisation of GG‐DHB to merosterolic acid A in the biosynthesis of cytotoxin merosterol A.

Through genome‐mining, Piel and co‐workers discovered the first example of a monodomain class II terpene cyclase, merosterolic acid synthase (MstE).^[85]^ The enzyme was shown to catalyse the cyclisation of GG‐DHB, to generate merosterolic acid A which can be further converted to the cytotoxin Merosterol A (Scheme 9). The MstE catalysed cyclisation of GG‐DHB is initiated by direct protonation of the terminal double bond, analogous to that observed for SHC's, and the carbocation is terminated by electrophilic aromatic substitution.^[86]^ Final re‐aromatisation produces merosterolic acid A. The group also noted that transformation of the natural substrate with MstE variant Y157A produced two products, merosterolic acid A and merosterolic acid B. The tricyclic product merosterolic acid A is thought to be produced via premature deprotonation of the methyl group due to rotation of the aromatic ring no longer being stabilised by Y157.

**Scheme 10:**
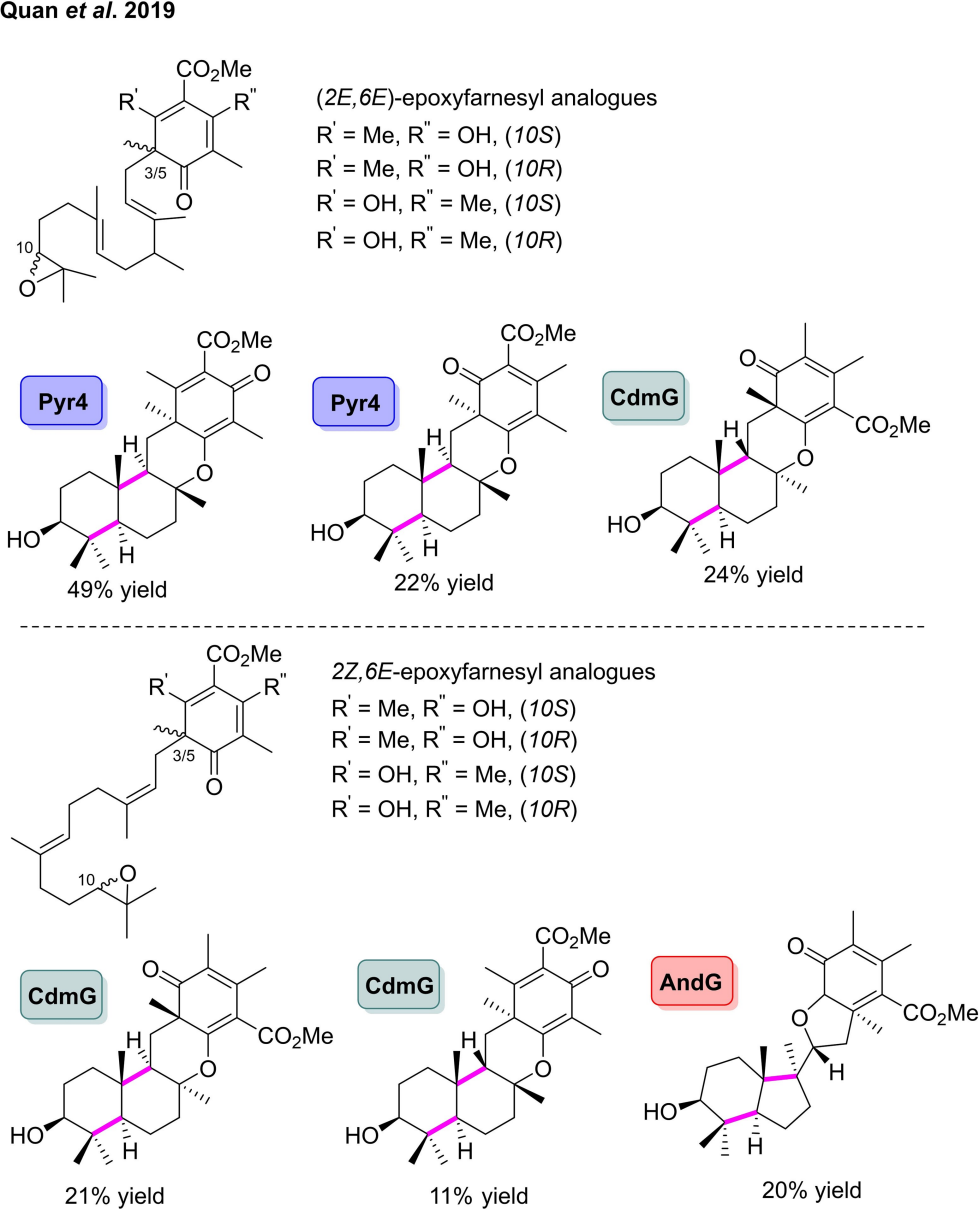
Enzymatic polycyclisation of synthetic epoxyfarnesyl analogues catalysed by a number of meroterpenoid cyclases.

In the laboratory of Abe, an oxidosqualene cyclase was identified in the biosynthetic pathway of ascochlorin from *Fusarium* sp.^[87]^ The enzyme, AsCFG, was able to cyclase the natural epoxy substrate, along with a brominated analogue, forming the unique trimethylcyclohexanone ring in the cyclised meroterpenoid products. The brominated product was found to display potent cytotoxicity towards the MCF‐7 breast cancer cell line. Building on from their previous work on fungal meroterpene cyclases, Abe and co‐workers investigated the reaction of eight linear terpenoids analogues, with nine previously reported meroterpenoid cyclases, Pyr4, CdmG, AndB, AdrI, NvfL, PrhH, Trt1, AscF and MacJ.[^88^, ^89^, ^90^, ^91^, ^92^, ^93^, ^94^, ^95^, ^96^] The group prepared eight epoxyfarnesyl analogues, starting from dimethylorsellinic acid (DMAO) (Scheme 10).^[21]^ The two (10*S*)‐(2*E*,6*E*)‐substrates where R’=Me, R’’=OH and R’=OH, R’’=Me were cyclised by Pyr4 to afford the tetracyclic products in 49 % and 22 % conversion, respectively. CdmG was also able to catalyse the cyclisation of the (10*S*)‐(2*E*,6*E*)‐substrates where R’=OH, R’’=Me, affording the desired product in 24 % yield. The (10*S*)‐(2*Z*,6*E*)‐substrates with R’=Me, R’’=OH and R’=OH, R’’=Me were both transformed by CdmG to novel meroterpenoids in 21 % and 11 % conversion, respectively. However, the most unusual reaction observed was the AndG catalysed cyclisation of (10*S*)‐(2*Z*,6*E*)‐substrate with R’=Me, R’’=OH to produce the **6**–**5** cyclised product in 20 % conversion. Crystalline sponge‐X‐ray analysis revealed the novel **6**–**5** ring system connected to a further **6**–**5** ring system through a single C−C bond. This work has the potential to revolutionise how fungal meroterpenoids are synthesised, addressing a crucial challenge in how the pharmaceutical industry accesses these highly bioactive compounds.^[21]^

**Scheme 11:**
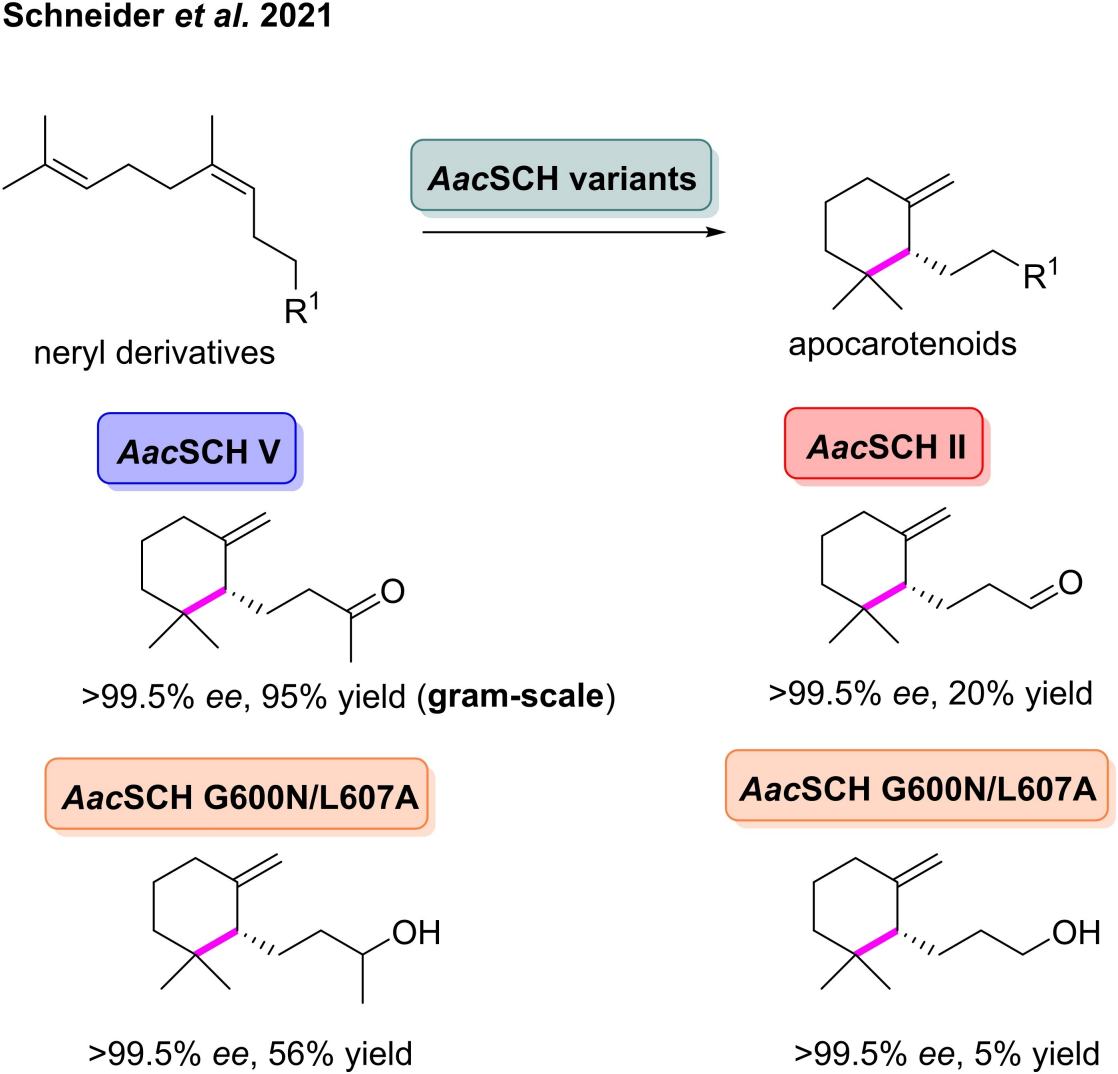
*Aac*SCH variants catalyse the highly stereoselective monocyclisation of neryl derivatives to furnish high‐value apocarotenoids.

As well as using genomics to discover cyclases which can generate novel terpenoids, it is also possible to engineer existing enzymes to influence the nature of the cyclisation and hence, the outcome of the reaction. Using site‐specific mutagenesis, Hoshino and co‐workers engineered the active‐site residues of the SHC from *Alicylobacillus acidocaldarius* (*Aa*SCH) to alter the normal polycyclisation.^[97]^ *Aa*SCH variants with increased steric bulk inside the active site (A306T and A306V) were reacted with squalene, leading to the accumulation of 6,6,6,5‐fused tetracyclic scaffolds in high yields. The structure of the observed products shows that increasing steric bulk at position 306 of *Aa*SHC significantly alters the stereochemical fate of the polycyclisation. Hauer *et al*. also used a similar approach to engineer *Aa*SHC to catalyse a novel cyclisation of citronella.^[98]^ The replacement of residues in the active site of *Aa*SHC resulted in two triple mutants which catalysed the cyclisation of (*R*)‐citronella to (+)‐*neo*‐isopulegol and (−)‐isopulegol with up to >99 % *dr*, respectively. The product of this cyclisation, (−)‐isopulegol, is an intermediate in the synthesis of (−)‐menthol, which is widely used in pharmaceuticals, fragrances and cosmetics.[^99^, ^100^] In 2021, the group further improved on their initial work by engineering *Aac*SHC to access a range of apocarotenoids, which are used in flavours, fragrances and in the pharmaceutical industry.^[16]^ Through site‐directed mutagenesis, the group could control cyclisation by anchoring dynamic substrates in the active site via designed hydrogen bonding. The native reaction of *Aac*SHC involves polycyclisation, generating bicyclic products, on the other hand the engineered *Aac*SHC variants were shown to only form monocyclic products.^[80]^ Conversion of the four neryl derivatives was observed with a number of *Aac*SHC variants (Scheme 11), however no reaction was observed for the wild‐type enzyme for any substrate. These variants catalysed a single C−C bond forming cyclisation, furnishing the corresponding 6‐membered products with *ee* values of >99.5 % and conversion of 5–95 %. Using the variant, *Aac*SHC *V*, the group was also able to access γ‐dihydroionone at a gram‐scale in 89 % isolated yield and >99.5 % *ee*, replacing previous multi‐step chemical syntheses.^[101]^

### 1.2 Diels‐Alderases

The Diels‐Alder (D−A) reaction is one of the most powerful transformations in a synthetic chemist's arsenal, enabling the construction of multiple C−C bonds with a high degree of stereoselectivity.[^102^, ^103^] The D−A reaction involves the [4+2] cycloaddition of a conjugate diene and a dienophile to form an unsaturated cyclohexane scaffold with a high degree of regio‐ and stereo‐selectivity.^[104]^ These D−A reactions can be classified as either intra‐ or inter‐molecular and, depending on the substituents, the reaction can occur via the normal‐ or inverse‐electron demand (NEDDA or IEDDA).[^102^, ^105^, ^106^] The electron demand of D−A reactions depends on the difference in energy levels between the frontier molecular orbitals (HOMO and LUMO) of the diene and dienophile.^[107]^

As the cyclohexane ring motif is ubiquitous in natural products, it has been speculated that [4+2] cyclising enzymes are involved in the biosynthesis of these secondary metabolites.[^108^, ^109^, ^110^] However, as these enzymes can exhibit dual activities, along with the controversy over their classification, the identification of specific enzymes that catalyses these reactions has proved very challenging.^[111]^ By the early 2000s, only three enzymes had been purported to catalyse D−A type reaction; macrophomate synthase, lovastatin nonaketide synthase (LovB) and solanapyrone synthase (Sol5), but confirming their influence on the cycloaddition step has not been easy.[^112^, ^113^, ^114^] The first publicised example of an enzyme catalysed D−A reaction was achieved via computational design of an artificial enzyme by Baker and co‐workers.^[115]^ The group used *de novo* protein design to build an active site which positions the diene and dienophile in the correct orientation for the reaction to occur. In 2011, Liu *et al*. identified a Diels‐Alderase, *Spn*F, in the spinosyn A biosynthetic gene cluster of *Saccharopolyspora spinosa*.^[116]^ The group found that the enzyme catalysed the intramolecular [4+2] cyclisation to generate the cyclohexene ring in spinosyn A with an estimated 500 fold rate enhancement. This is the first widely accepted example of an enzyme catalysed intramolecular D−A reaction. Since the characterisation of *Spn*F, technological advancements in genome mining have resulted in the discovery of many D‐Alderases in the biosynthetic pathways of bacteria, fungal and plant origins.[^109^, ^110^, ^111^]

**Scheme 12:**
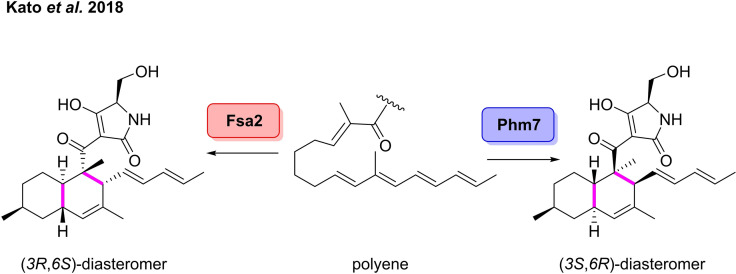
Diastereoselective Diels‐Alderases in the synthesis of decalin scaffolds.

The intramolecular D−A reaction is proposed as the key reaction step in the biosynthesis of decalin‐containing fungal polyketides.[^110^, ^117^] These decalin‐containing natural products display a broad spectrum of biological activities.^[118]^ In 2015, Osada and co‐workers first outlined a gene cluster, *fsa*, involved in the biosynthesis of a number of biologically active decalin‐containing pyrrolidin‐2‐ones in *Fusarium* sp.^[119]^ Examples of these bioactive compounds includes the HIV‐1 integrase inhibitors, equisetin and phomasetin.^[120]^ Through genetic analysis, a new enzyme, *fsa*2, was also identified which was involved in the [4+2] cycloaddition to form the decalin‐containing pyrrolidin‐2‐one scaffold. The group further built on this through the discovery of a homologue of *fsa*2 which is involved in the biosynthesis of the enantiomerically opposite *trans*‐decalin analogue (Scheme 12).^[121]^ Another *fsa* homologue, *phm*7, was identified from the biosynthetic gene cluster of phomasetin in *Pyrenochaetopsis* sp*. Phm7* was shown to catalyse the formation of the (3*S*,6*R*)‐diastereomer. Gene replacement of *phm*7 with *fsa2* in the gene cluster also enabled the formation of the decalin skeleton with a non‐natural (3*R*,6*S*)‐configuration. This is the first example of a Diels‐Alderase being exploited to afford a non‐natural analogue, and excellently demonstrates how these enzymes have untapped potential for the production of natural products with unnatural stereochemistries.

**Scheme 13:**
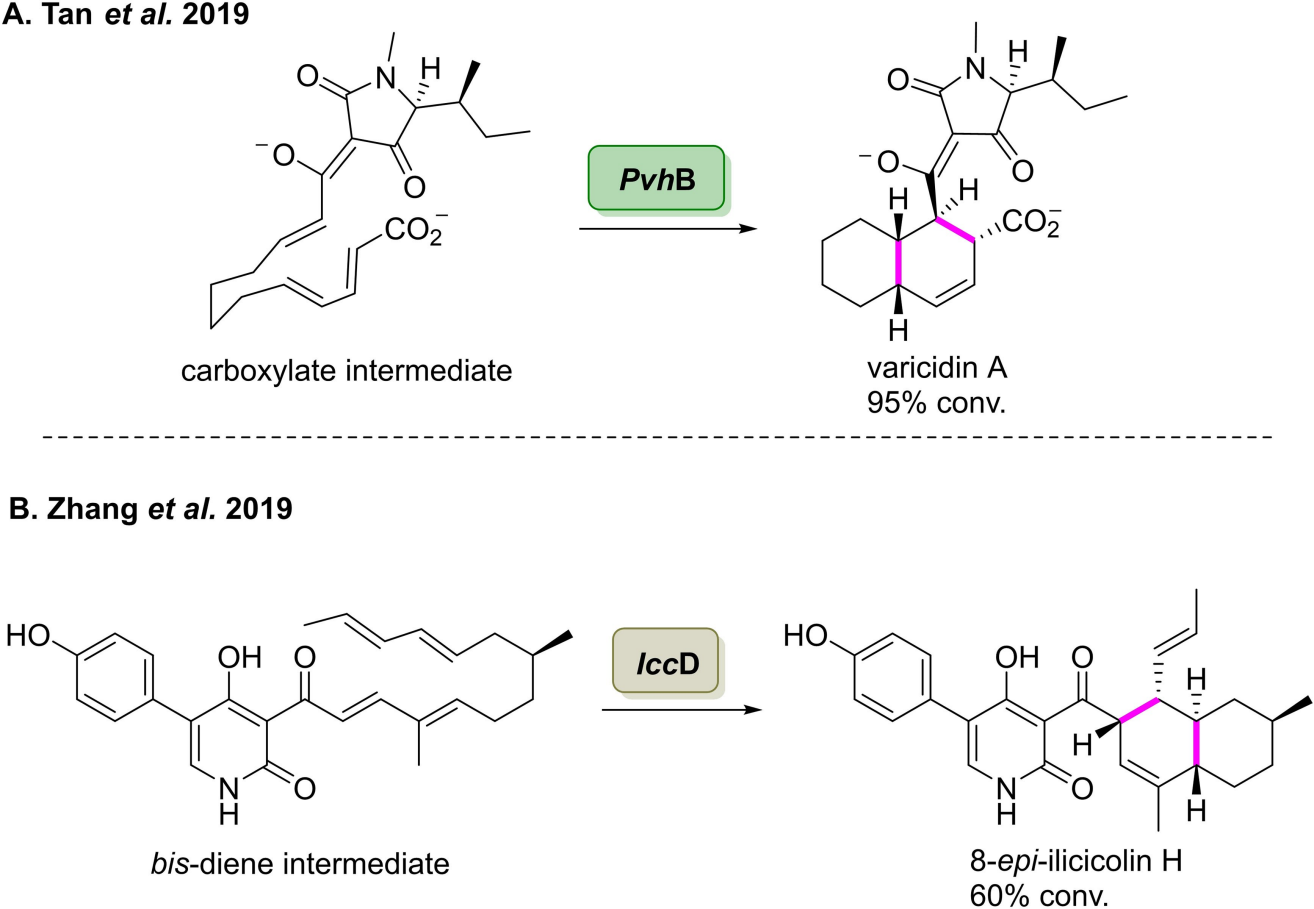
(A) A novel Diels‐Alderase, *Pvh*B, catalyses the endo‐selective Diels‐Alder reaction of the carboxylate intermediate in the biosynthesis of varicidin A. (B) Enzymatic IED Diels‐Alder reaction of *bis*‐diene to furnish 8‐*epi*‐ilicicolin H catalysed by a novel Diels‐Alderase, *Icc*D.

The above enzymes, which are involved in decalin biosynthesis, CghA and Fsa2 are examples of NED Diels‐alder reactions which proceed through the *endo* transition state.[^119^, ^121^, ^122^, ^123^] In 2019, Tang and co‐workers outlined the first example of an enzyme in this family catalysing an intramolecular IED Diels‐Alder reaction.^[124]^ Using genome mining, the group found a Diels‐Alderase, *Pvh*B, which is involved in the biosynthesis of varicidin A. The enzyme was demonstrated to catalyse the final step in the biosynthetic pathway, involving an intramolecular IED Diels‐Alder reaction of a carboxylate intermediate to form the varicidin A in 95 % conversion (Scheme 13A). The group also identified another IED Diels‐Alderase, *Icc*D, which catalyses the intramolecular IED Diels‐Alder reaction in the biosynthesis of ilicicolin H, an alkaloid that displays potent antifungal activities towards a broad range of fungal pathogens.^[125]^ *Icc*D was found to catalyse the intermolecular IEDDA reaction from a *bis*‐diene, affording 8‐*epi*‐ilicicolin H in 60 % conversion. 8‐*epi*‐Ilicicolin H was then further converted to ilicicolin H *via* enzymatic epimerisation (Scheme 13B). These two pieces of work highlight the importance of characterising enzymes which exhibit novel reactivities and can be used to synthesis new natural product analogues.

**Scheme 14:**
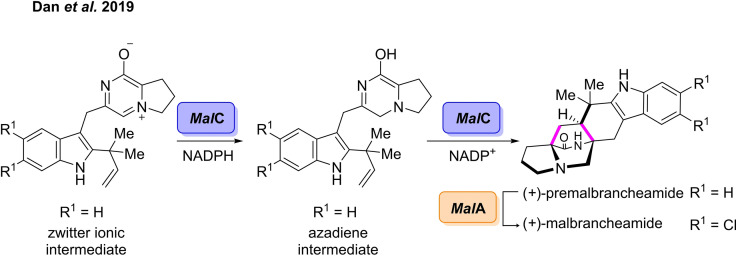
Biocatalytic synthesis of (+)‐malbrancheamide involving the bifunctional NADPH‐dependent reductase/Diels‐Alderase *Mal*C.

Previously, all known examples of Diels‐Alderases were either redox‐neutral cyclases or oxidases.[^113^, ^116^, ^121^, ^124^, ^125^, ^126^] However, Williams and co‐workers have described the first example of a reductase‐dependent Diels‐Alderase, which is involved in the biosynthesis of malbrancheamide.^[127]^ The complex bicyclo[2.2.2]diazaoctane core of these prenylated indole alkaloids has attracted attention as a target for total synthesis strategies and these compounds also display a range of biological activities.[^128^, ^129^] The group was able to elucidate the malbrancheamide biosynthetic pathway, revealing an enzyme catalysed [4+2] Diels‐Alder cyclisation as the fundamental step which generates the bicyclo[2.2.2]diazaoctane skeleton. The bifunctional NADPH‐dependent reductase/Diels‐Alderase, *Mal*C, initially catalyses the reduction of the zwitterionic intermediate to furnish the azadiene intermediate. *Mal*C subsequently catalyses the stereoselective intramolecular NEDDA cyclisation of the reduced intermediate to form (+)‐premalbrancheamide. The final step in the biosynthetic pathway involves the halogenation of premalbrancheamide, which is catalysed by the halogenase, *Mal*A, to furnish (+)‐malbrancheamide (Scheme 14).

**Scheme 15:**
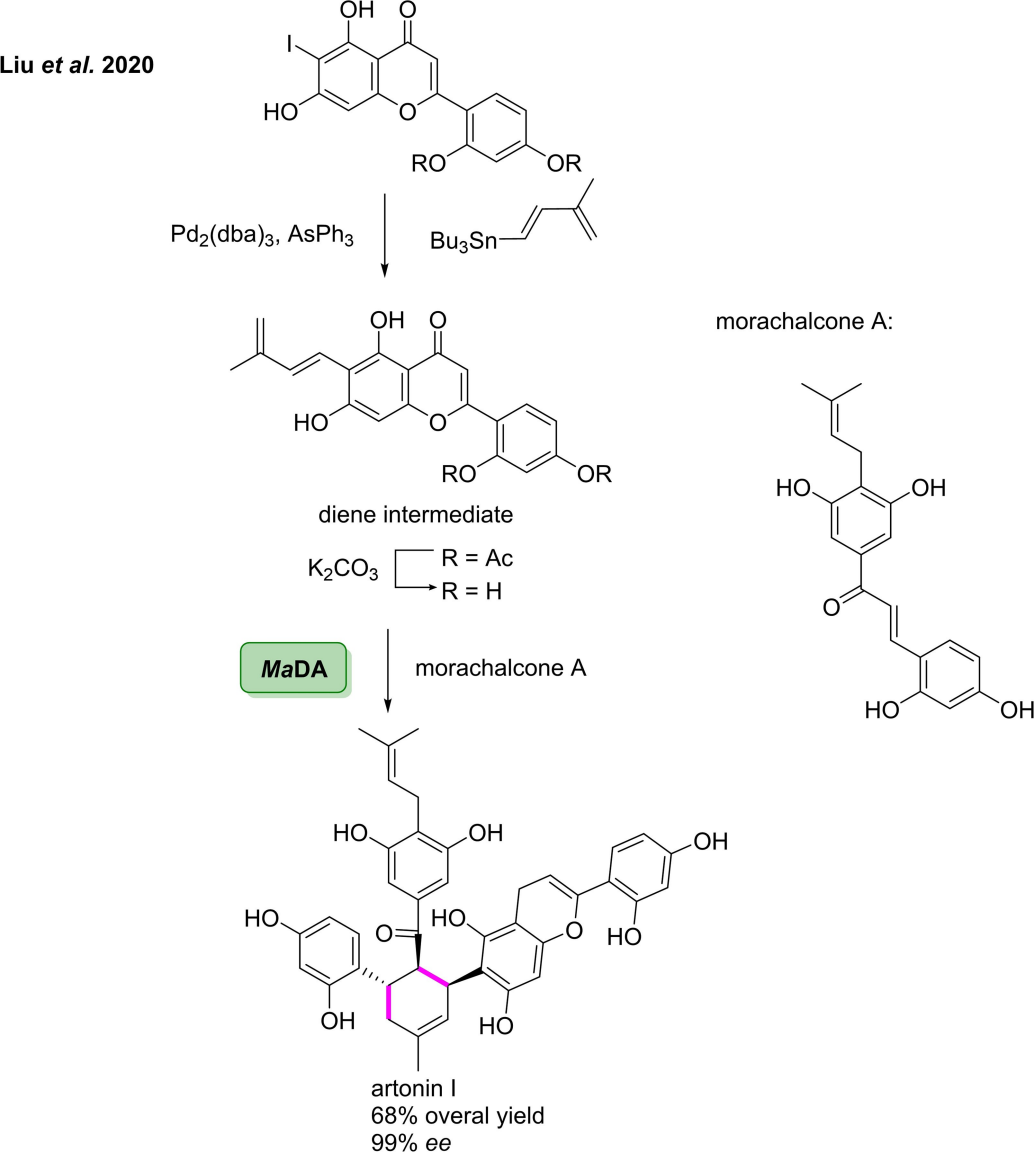
Chemoenzymatic synthesis of Artonin I involving the Diels‐Alderase *Ma*DA.

Enzymes capable of catalysing intermolecular reactions are of significant importance, as the coupling of two molecules can be used to generate more structurally diverse products. In 2020, Lei *et al* reported the first example of an intermolecular Diels‐Alderase.^[126]^ The enzymatic intermolecular Diels‐Alder reaction was proposed to occur in the biosynthesis of the isoprenylated flavonoid chalcomoracin, a natural product isolated from the root bark of the Morus plant.^[130]^ This bark has been used extensively in Traditional Chinese Medicine and natural products from this family have shown anti‐HIV and anti‐diabetic activities.[^131^, ^132^] Using a combination of activity‐based protein purification and proteomics analysis, termed BIP‐based target identification, the first intermolecular Diels‐Alderase, *Ma*DA, from *Morus alba* was characterised. The enzyme was shown to catalyse the cycloaddition of morachalcone A and a diene to afford chalcomoracin in 51 % yield. A panel of synthetic diene and morachalcone analogues were also produced which the enzyme was able convert to several isoprenylated flavonoid derivatives. The enzyme was also shown to catalyse the cycloaddition of synthetic trihydroxylated diene with morachalcone A, furnishing guangsangon in 62 % yield. The Lei group went on to develop a chemoenzymatic total synthesis of artonin I involving Stille cross‐coupling and enzymatic intermolecular Diels‐Alder reaction.^[133]^ Artonin I belongs to the same family of isoprenylated flavonoids, and has been shown to inhibit multidrug resistance in *Straphylococcus aureus*.^[134]^ A diene analogue was synthesised *via* palladium catalysed cross‐coupling in 75 % yield which was then hydrolysed to the desired intermediate prior to cyclisation (Scheme 15). *Ma*DA was then used to catalyse the intermolecular [4+2] cycloaddition with morachalcone A, affording endo‐Artonin I in 90 % yield and 99 % *ee*.

### 1.3 Biocatalytic Pictet‐Spengler reactions

For over 100 years the Pictet‐Spengler (PS) reaction has been used by organic chemists to construct the carbon framework in a broad range of heterocyclic products.^[135]^ The reaction involves the acid‐catalysed condensation of β‐arylethylamines with aldehydes to generate tetrahydroisoquinoline (THIQs) or tetrahydro‐β‐carbolines (TH‐β‐Cs) via an iminium intermediate. These alkaloid motifs are widespread in bioactive natural products and are important building blocks for many pharmaceutically relevant drug molecules. The most notorious family of THIQ alkaloids are the narcotic analgesics morphine and codeine which are derived from the opium poppy (*Papaver somniferum*).^[136]^ The β‐carboline scaffold is also present in numerous active pharmaceuticals including the anti‐malarial cipargamin, anti‐cancer lurbinectedin and the erectile dysfunction drug tadalafil.[^135^, ^137^, ^138^, ^139^, ^140^] Currently, numerous strategies have been outlined for the enantioselective Pictet‐Spengler reaction including organo‐ and transition‐metal catalysis.[^141^, ^142^] However, it was over half a century after the discovery of the chemical transformation before an enzyme catalysed Pictet‐Spengler reaction was reported.^[135]^ The first Pictet‐Spenglerase, named strictosidine synthase (STR), catalyses the condensation of tryptamine and the monoterpenoid aldehyde secologanin followed by cyclisation to give the tetrahydro‐β‐carboline (*S*)‐strictosidine.^[143]^ The substrate scope of the STR catalysed reaction is mainly limited to derivatives of secologanin as the aldehyde component, but success with various substituted tryptamines have been reported.^[1]^ The other prominent member of the Pictet‐Spenglerase family are the norcoclaurine synthases (NCSs), which are involved in the biosynthesis of tetrahydroisoquinoline alkaloids.^[144]^ The native reaction catalysed by NCSs involves the stereoselective C−C coupling of dopamine with 4‐hydroxyphenylacetaldehyde to afford (*S*)‐norcoclaurine.^[135]^ Current research in this area has focused on the genetic engineering of current enzymes and expanding the non‐natural substrate scope of these reaction to enable the synthesis of novel alkaloids.

**Scheme 16:**
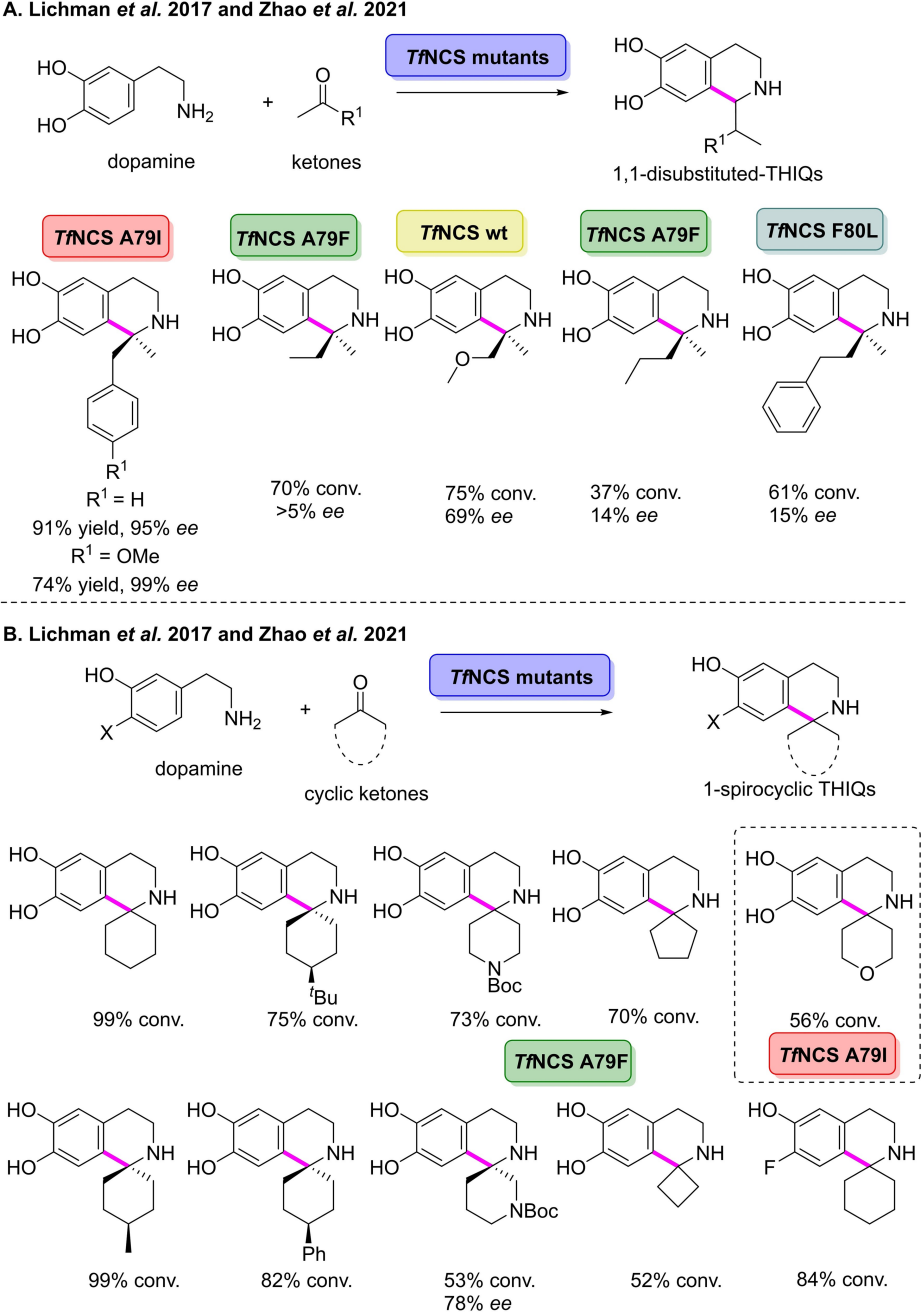
(A) Pictet‐Spengler reaction between dopamine and a number of substituted methyl ketones catalysed by norcoclaurine synthase mutants. (B) Pictet‐Spengler between dopamine with cyclic ketones catalysed by norcoclaurine synthase mutants.

In 2017, Ward and co‐workers reported the first example of NCS catalysing the PS reaction between the natural substrate dopamine and unactivated ketones.^[145]^ The approach enabled stereoselective access to 1,1’‐disubstituted and spiro‐THIQs, for which there are no current asymmetric chemicals methods.^[146]^ The group selected two wild‐type NCSs, Δ29*Tf*NCS and C*j*NCS2, to screen against the natural substrate dopamine and 4‐hydroxyphenylacetone.[^147^, ^148^] After initial investigation it was found that the wild‐type enzyme from *Thalictrum flavum*, *Tf*NCS, catalysed the PS reaction. Docking studies were then used to rationally select enzyme variants with increased substrate tolerance for the ketone partner in the reaction. The *Tf*NCS variant A79I was used in the preparative‐scale PS reaction of phenylacetone with dopamine, affording the corresponding 1,1’‐disubstituted derivative in 87 % isolated yield and 95 % *ee* (Scheme 16A). *Tf*NCS A79I was also shown to catalyse the PS reaction of *para*‐methoxy phenyl acetone with dopamine, affording the THIQ derivative in 74 % yield and 99 % *ee*. Furthermore, *Tf*NCS A79F was demonstrated to catalyse the reaction of four cyclohexanone derivatives with dopamine to furnish spiro‐THIQ derivatives in 33–58 % isolated yields. In 2021, the group built on this work by further increasing the substrate scope of the reaction towards increasingly complex ketones and a number of dopamine analogues.^[149]^ Initially, a panel of substituted methyl ketones were examined in the PS reaction with dopamine using a panel of *Tf*NCS variants, affording a number of substituted THIQ derivatives in 37–75 % conversion (Scheme 16B). A panel of cyclic ketones was then utilised to synthesise a number of spirocyclic THIQs. Reaction of dopamine with N‐Boc protected 4‐piperidone catalysed by *Tf*NCS variant A79F afforded the spirocyclic THIQ in 73 % conversion after 72 h. When the carbonyl was altered to N‐Boc protected 3‐piperidone, the corresponding spirocyclic THIQ was achieved in 53 % conversion and an *ee* of 78 %. Substitution of the nitrogen atom to an oxygen atom in the six‐membered ring gave the THIQ derivative in 56 % conversion. Four cyclic ketones with varying ring sizes were then used to show the influence of ring size in the PS reaction. Reaction of cyclobutanone with dopamine using *Tf*NCS variant A79F afforded the spiro‐THIQ in 52 % conversion. Increasing the ring size to cyclopentanone gave the corresponding THIQ derivative in 70 % conversion using the same *Tf*NCS variant. Cyclohexanone was reacted two dopamine derivatives to afford the corresponding THIQs in 54 and 84 % conversion, respectively. The work carried out in this area enables the stereoselective coupling of ketones in the PS reaction, a transformation which, via current synthetic approaches is not trivial.

**Scheme 17:**
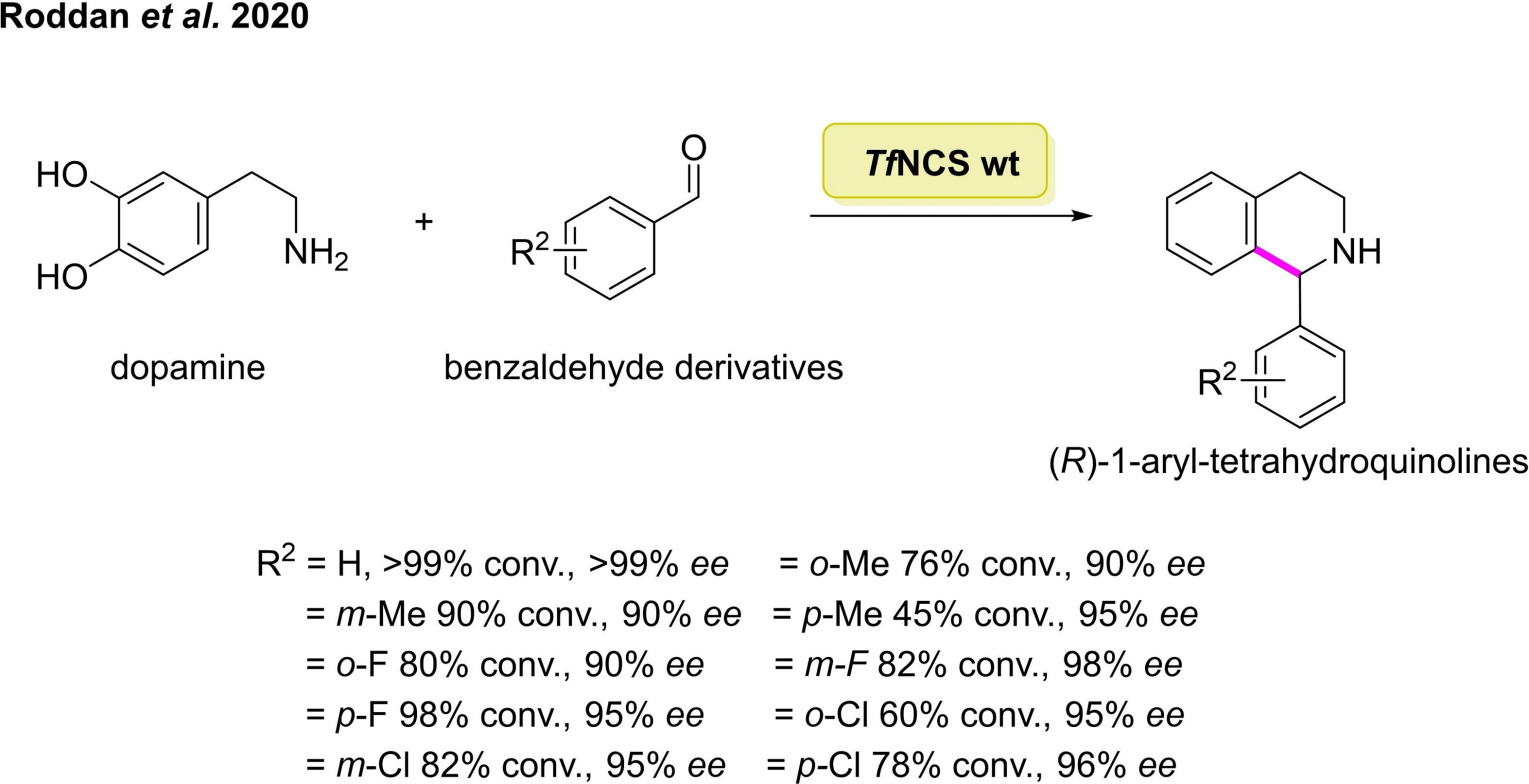
Pictet‐Spengler reaction between dopamine and a panel of substituted benzaldehydes catalysed by wild type norcoclaurine synthase (NCS).

The aldehyde scope in the NCS catalysed PS reaction has also received significant interest.[^150^, ^151^, ^152^] Hailes and co‐workers outlined a concise, stereoselective preparation of 1‐aryl THIQs utilising aryl aldehydes as the carbonyl partner in the NCS catalysed PS reaction.^[153]^ The 1‐aryl THIQ core is present in a number of APIs including the muscarinic‐receptor antagonist solifenacin.^[154]^ After initial screening, the *Tf*NCS variant M97V was found to be the most productive, catalysing the reaction of benzaldehyde with dopamine, furnishing 1‐aryl THIQ in >99 % conversion with >99 % *ee*. Methyl substituted benzaldehydes were also examined in the PS reaction with dopamine, affording the corresponding substituted 1‐aryl THIQs in 76 %, 90 %, and 45 % conversion, respectively (Scheme 17). Several halogenated benzaldehydes were also investigated with dopamine catalysed by the M97V‐*Tf*NCS mutant, achieving the desired THIQ derivatives in good to excellent yield and with a high degree of stereoselectivity. Although several other biocatalytic and chemically methodologies have been outlined for the stereoselective synthesis of 1‐aryl‐THIQs,[^155^, ^156^, ^157^] this approach generates the desired product in a single step from widely available starting materials.

**Scheme 18:**
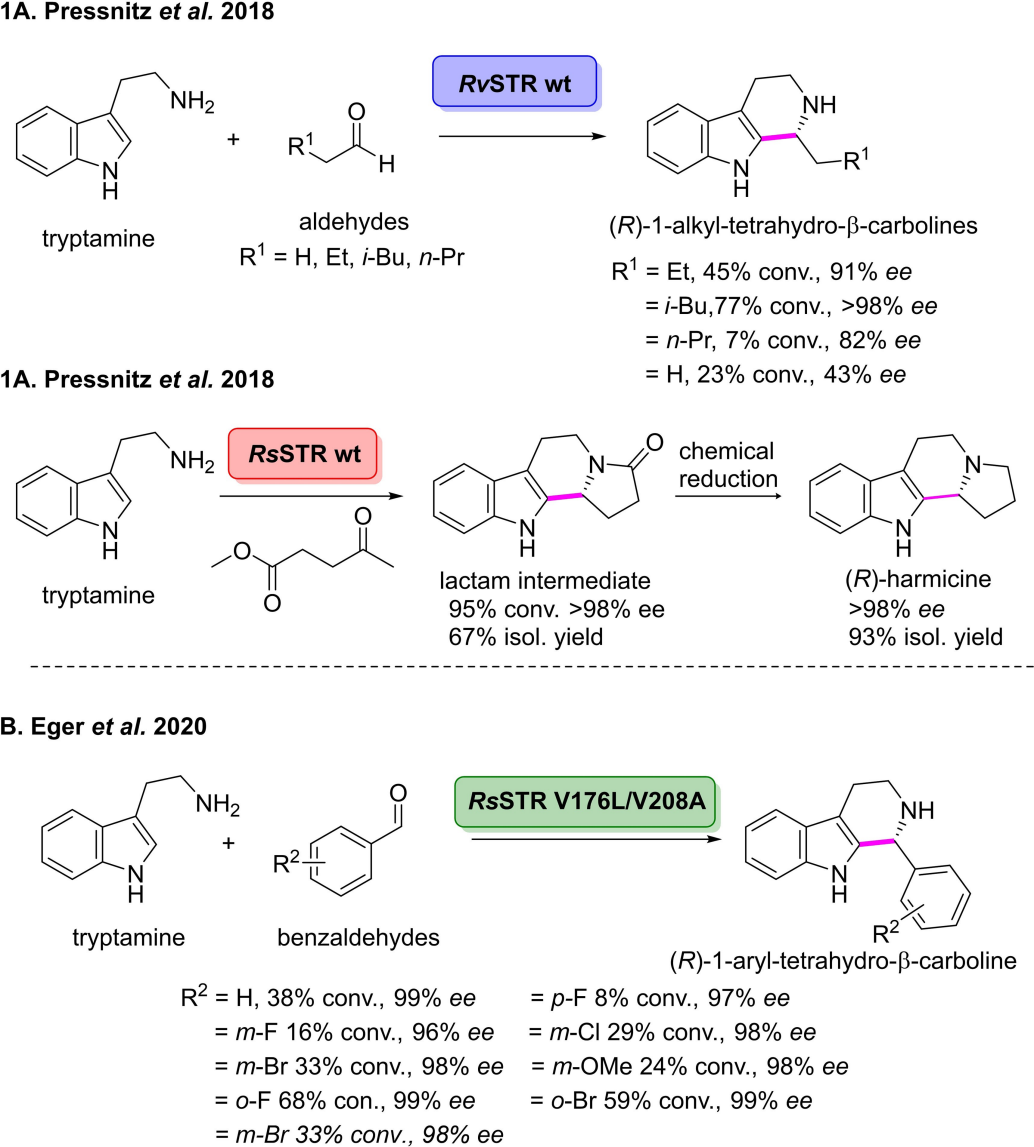
1 (A) Strictosidine synthase (STR) catalysed Pictet‐Spengler reaction between tryptamine and non‐natural aliphatic aldehydes. 2 (A) Chemoenzymatic total synthesis of (*R*)‐harmicine in two steps from commercially available starting materials. (B) Enzymatic Pictet‐Spengler reaction between tryptamine and benzaldehydes catalysed by a strictosidine synthase mutant.

In 2018, Kroutil and co‐workers used STR to prepare a number of alkyl substituted tetrahydro‐β‐carbolines with a high degree of stereoselectivity.^[158]^ These substituted tetrahydro‐β‐carbolines are key intermediates in many bioactive molecules, including the alkaloid (*R*)‐harmicine which displays potent anti‐Leishmania activity.[^159^, ^160^] Four native STRs originating from different organisms were initially chosen for screening; *Catharanthus roseus* (*Cr*STR), *Ophiorrhiza pumila* (*Op*STR), *Rauvolfia serpentine* (*Rs*STR) along with the V208A variant of *Rv*STR.[^161^, ^162^, ^163^, ^164^] The group then examined the PS reaction between the natural substrate tryptamine and a small panel of aldehydes (Scheme 15). *Rs*STR was able to effectively catalyse the reaction with isovaleraldehyde and 4‐oxobutanoate, affording the corresponding tetrahydro‐β‐carboline derivatives in 77 % and 95 % conversion, respectively, and with >98 % *ee*. Increasing the aldehyde chain length led to a significant decrease in conversion from 45 % to 7 % but both reactions retained good enantioselectivities. Ethanal was also converted by *Rv*STR in 23 % conversion and with an *ee* of 43 %. Interestingly, it was found that all reactions afforded the corresponding (*R*)‐enantiomer, which is opposite to the enzymes native reaction leading to (*S*)‐configured products. The group scaled up the *Rs*STR catalysed PS reaction of 4‐oxobutanoate with tryptamine, initially affording the 1‐substituted tetrahydro‐carboline prior to spontaneous lactimisation to furnish the corresponding lactam in 67 % isolated yield and >98 % *ee*. Chemical reduction gave (*R*)‐harmacine in 62 % yield over two steps and excellent *ee* of >98 %. This chemoenzymatic approach to (*R*)‐harmacine is the shortest and highest yielding report which has been published to date.[^158^, ^165^] This work has demonstrated a novel method for the stereoselective synthesis of pharmaceutically relevant tetrahydro‐β‐carbolines, and hence has increased the toolbox of current biocatalytic C−C bond forming reactions. The group then expanded the aldehyde scope of the STR catalysed PS reaction towards a number of substituted aromatic aldehydes.^[166]^ Initially, two native STRs, *Ophiorrhiza pumila* (*Op*STR) and *Rauvolfia serpentine* (*Rs*STR) were chosen for screening. However, neither of the wild‐type STRs were found to catalyse the conversion of benzaldehyde, so a ‘substrate‐walking approach’ was used to prepare a panel of variants which were subsequently transferred to *Rs*STR. The double mutant, *Rs*STR V176L/V208A, was found to efficiently catalyse the PS reaction of benzaldehyde with dopamine, affording 1‐aryl‐tetrahydro‐β‐carboline in 38 % conversion and 99 % *ee* (Scheme 18). A small panel of substituted aromatic aldehydes was then screened with the double *Rs*STR variant to determine the substrate scope in the PS reaction. o*rtho*‐Halogenated benzaldehydes were transformed to the corresponding 1‐aryl substituted THBCs in good yields and excellent enantioselectivity. However, the *para*‐fluorinated derivative was not well accepted (8 % conversion and 96 % *ee*). *meta*‐Substituted derivatives were transformed by the enzyme, affording *meta*‐substituted 1‐aryl THBCs in 29 %, 33 %, and 24 %, respectively, in 99 % *ee*. Further *meta*‐substituted benzaldehydes were also both accepted by the enzyme, affording the corresponding THBC derivatives in low to moderate conversions and excellent *ee* values. This work has expanded the scope of stereoselective PS reactions, enabling the preparation of tetrahydro‐β‐carboline scaffolds which are challenging to access with current chemical approaches.

**Scheme 19:**
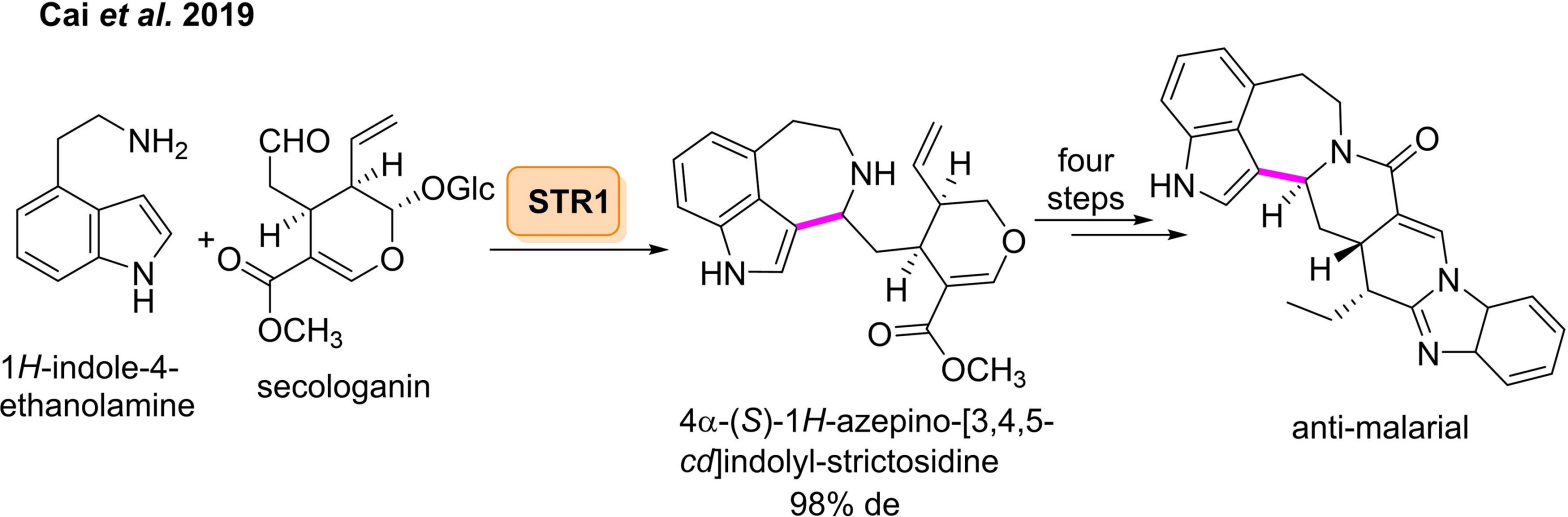
Enzyme catalysed Pictet‐Spengler reaction of non‐natural 1*H*‐indole‐4‐ethanolamine towards azepino‐indole scaffolds.

Although the aldehyde scope of STR has been widely examined, the scope of the amine partner in the PS reaction has not generated the same amount of interest.[^167^, ^168^] In 2019, a chemoenzymatic route towards azepino[3,4,5‐*cd*]‐indoles was outlined by Zou *et al*, involving an enzyme catalysed PS reaction of a tryptamine analogue with the natural substrate secologanin, followed by chemical transformation to attain the biologically active product.^[169]^ Molecules containing the azepino[3,4,5‐*cd*] indole core have displayed dopamine receptor binding activity, showing potential as drugs for neuropsychiatric diseases.^[170]^ The group reported the PS reaction between tryptamine analogue 1*H*‐indole‐4‐ethanamine and secologanin catalysed by STR1 to furnish a tricyclic intermediate with >98 % *de*, which was subsequently cyclised to afford the corresponding lactam in 24 % isolated yield (Scheme 19). The lactam was then converted in four steps to the azepino[3,4,5‐*cd*] indole with an overall yield of 26 %. This compound was found to display antimalarial activity, with an IC_50_ of 6.1±0.3 μM towards *Plasmodium falciparum* strain 3D7.

## 2 Biocatalytic C−N Bond Formation via Reductive Amination

**Figure 1:**
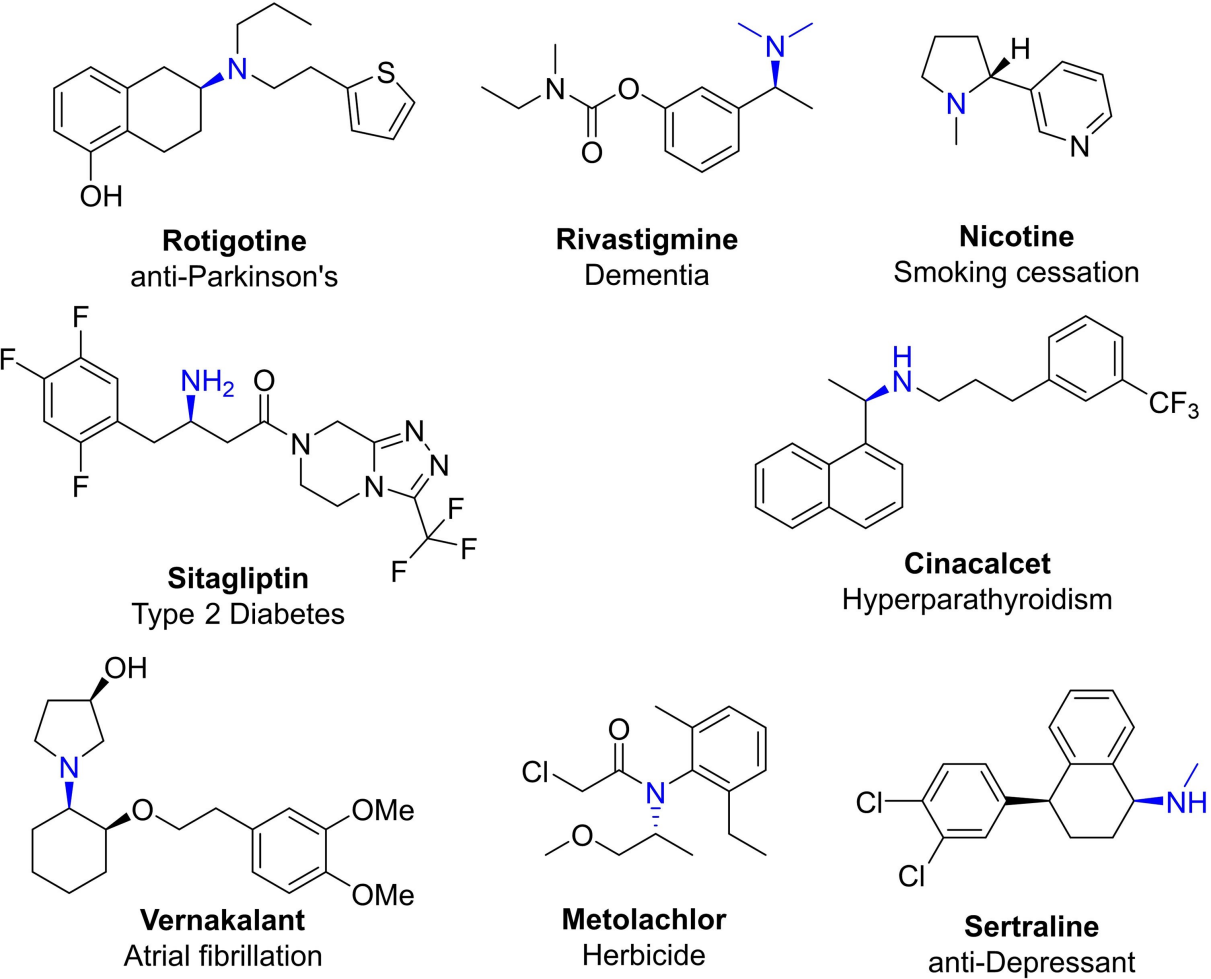
An overview of bioactive chiral amine containing compounds.

Primary, secondary, and tertiary amines are a highly prevalent motifs in a range of biologically active molecules within the pharmaceutical, agrochemical and fine chemical markets. Amines with chiral centres are of particular interest, where biocatalytic approaches are inherently selective in generating chiral amines, outcompeting traditional synthetic approaches.^[171]^ Figure 1 highlights the diversity of optically active molecules with a chiral amine constituent, where the relevant function is highlighted.

Reductive amination of a compound bearing a carbonyl moiety coupled with an amine nucleophile via an iminium intermediate to the corresponding (chiral) amine is highlighted as one of the most important and diverse reactions to generate chiral amines. Chemical approaches to asymmetric reductive amination include the use of transition metal catalysis such as ruthenium or rhodium, utilising either a reducing agent^[172]^ or H_2_.^[173]^ Organocatalytic methods employ chiral Brønsted acids to induce chirality coupled with Hantzsch esters^[174]^ or hydrosilanes^[175]^ for the hydrogen source. Although these listed techniques are well established, there are safety and environmental concerns surrounding these approaches from the use of hydrogen as a reducing agent, to the implementation of rare, costly, and toxic metal catalysts. Furthermore, reductive amination often requires an excess of the amine to prevent over alkylation, complicating downstream work‐up procedures.^[176]^

Enzymatic routes in the preparation of chiral amines offer to bypass some of the above problems. These routes utilise enzymes which are biodegradable, operate under benign conditions, and present a high degree of chemo‐, regio‐ and enantioselectivity, furthering sustainable routes to chiral amines.^[177]^ Selecting an enzyme from the chiral amine toolbox for a particular route has never been easier due to the forever expanding sequence space coupled with retrosynthetic analysis tools such as RetroBioCat.^[178]^ Where scopes of these enzymes may overlap, the mechanism by which they operate greatly differs. This has its advantages and drawbacks for each enzyme family. Some enzymes such as oxidases and transaminases have been readily amenable to evolution enhancing substrate scopes. Nevertheless, limitations with certain families exist due to the nature of their mechanism, such as transaminases, utilising a pyridoxal 5‐phosphate (PLP) co‐factor which yield strictly primary amines, thus requiring further alkylation chemistry to access chiral secondary and tertiary amines.

However, in the past decade, tremendous efforts have been invested in enzymes that generate chiral amines through reductive amination. These enzymes will be covered in this section of the review. These include the families of amine dehydrogenases, imine reductases, *N*‐methylamino acid dehydrogenases, ketimine reductases, and pyrroline‐5‐carboxylate reductases. These enzymes belong to the oxidoreductase class and operate using a nicotinamide cofactor to deliver the hydride, thus generating a plethora primary, secondary and tertiary amines.

### 2.1 Imine reductases (IREDs) and reductive aminases (RedAms)

Imine reductases were first discovered and applied initially for the reduction of pre‐formed 5‐membered cyclic imines to generate the corresponding amine in 2010 by Mitsukura and co‐workers.^[179]^ Two separate enantiocomplementary enzymes from *Streptomyces* sp. were identified generating both (*R*)‐ and (*S*)‐2‐methylpyrrolidine. Significant progress was made towards expanding the scope and applicability of these enzymes towards imine reductions, which is covered in‐depth in reviews by and Grogan *et al*.^[180]^ and Mangas‐Sanchez *et al*.^[181]^ As both reductive amination and imine reduction share a common imine intermediate, it seemed plausible to investigate whether IREDs could perform reductive amination. The first application of IREDs for reductive aminations was given in 2014 by Huber and co‐workers,^[182]^ who took (*S*)‐IRED from *Streptomyces* sp. discovered by Mitsukura and co‐workers^[179]^ and applied it for the reductive amination of 4‐phenyl‐2‐butanone in methylammonium buffer as the amine source. Following up this report quickly, different academic and industrial groups explored the use of IREDs for reductive amination,[^183^, ^184^, ^185^, ^186^] although these initial studies described processes in which a basic pH and a large excess of the corresponding amine were required to obtain high conversions, hence limiting their applicability into large‐scale processes.

#### 2.1.1 A reductive aminase from *Aspergillus oryzae*

**Scheme 20:**
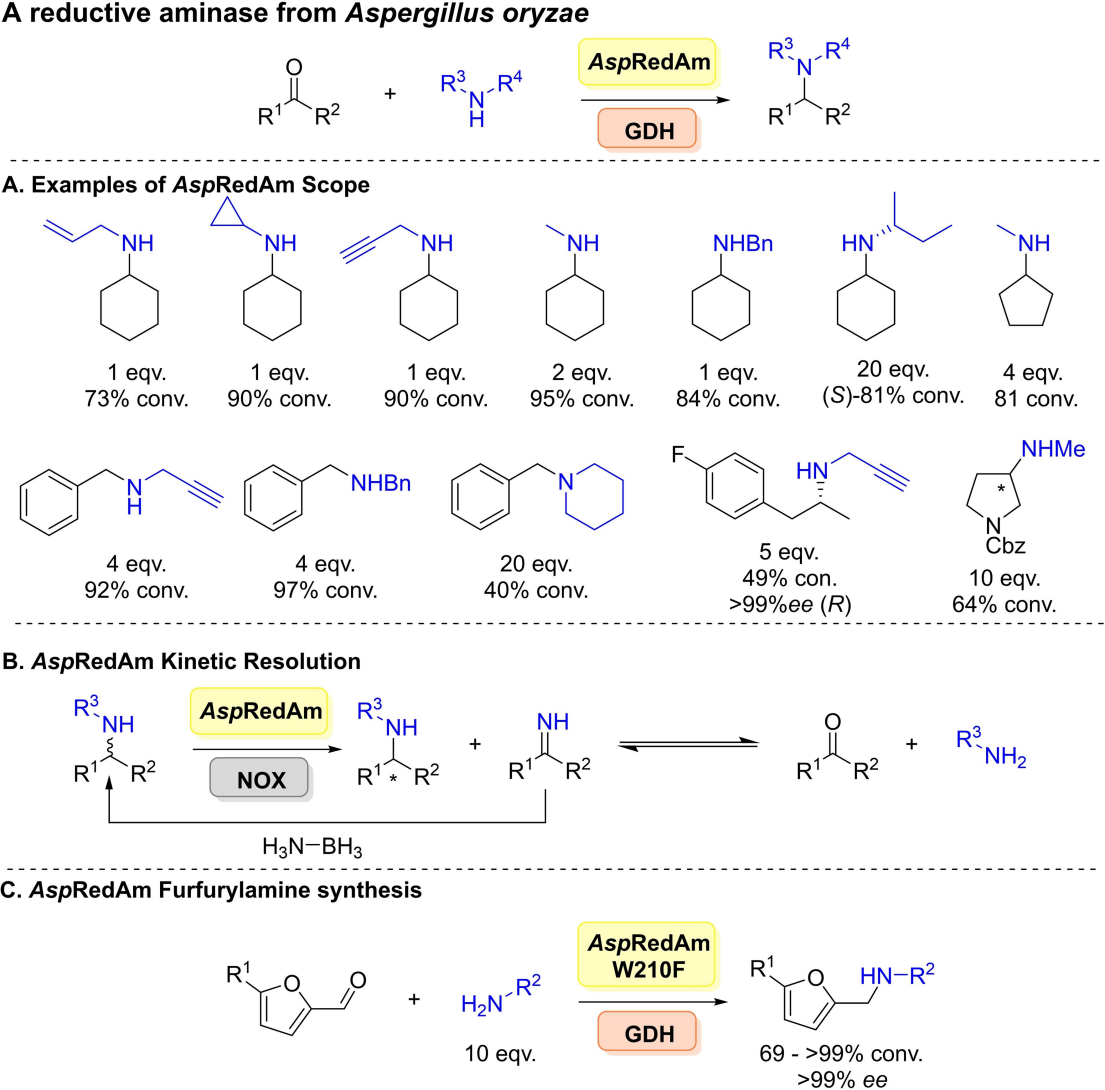
A reductive aminase from *Aspergillus oryzae*. (A) Examples of the scope of *Asp*RedAm. (B) Application of *Asp*RedAm for the kinetic resolution of racemic amines combined with ammonia borane and an NADPH‐oxidase (NOX). (C) Synthesis of furfurylamines from bio‐based furans.

All previous reports around IRED mediated reductive aminations had demonstrated IREDs working with large excesses of amine equivalents, operating at higher pH values, where the scopes did not appear to be particularly broad, overall limiting the synthetic viability. The discovery of an enzyme and several homologues by the Turner laboratory in 2017 addressed many of these issues mentioned.^[187]^ Where conversions of 94 % were achieved employing 1 : 1 ratios of ketone to amine, with other examples of low amine equivalents yielding high conversions. The enzyme was termed a reductive aminase from *Aspergillus oryzae* (*Asp*RedAm) and was the first enzyme in the field to be of fungal origin, with two other homologues. These fungal enzymes form a specific sub‐clade within the sequence space of the IRED family as highlighted by phylogenetic analysis in later studies.^[188]^ *Asp*RedAm was shown to act on a broad range of aldehydes and ketones coupled with primary and secondary amines (Scheme 20A). Several studies conducted coupled with the enzyme's unusual activity in comparison to previous IREDs coined the term reductive aminase. Highlighting the enzyme catalysed both imine formation and reduction. A pH study (pH 7.0 vs 9.0) was conducted comparing rates for the reductive amination of cyclohexanone with allylamine compared to previously studied IREDs, (*R*)‐IRED and *Ao*IRED,^[189]^ demonstrating *Asp*RedAm displayed a significantly higher specific activity irrespective of pH. Through structural data analysis, where a ternary complex was obtained with (*R*)‐rasagiline combined with point mutagenesis, which identified 6 key catalytic residues N93, D169, Y177, W210, M239 and Q240. The residue Q240 was further targeted to alter substrate specificity, and indeed the variant Q240A yielded the anti‐Parkinson's agent (*R*)‐rasagiline with 98 % *ee*, higher than previous reports.

In a follow up study in 2018, a deeper insight into the mechanism of *Asp*RedAm's activity and its fungal homologues was given through structural and mechanistic studies coupled with mutagenesis.^[190]^ *At*RedAm was co‐crystallised with cyclohexanone and allylamine suggesting residues Y183 was involved in carbonyl recognition and D175 involved amine recognition. A proposed mechanism was also proposed for both imine formation catalysis and imine reduction, where for productive catalysis both amine and ketone must be bound leading to domain closure and generating a smaller active site. Activity of the fungal enzymes was compared to (*S*)‐IRED from *Streptomyces sp*. and NaBH_3_CN, where *At*RedAm gave 73 % conversion compared 4 % for the latter two after 3 h. This difference a good indication towards such activity.

Extending the synthetic scope of *Asp*RedAm and to demonstrate its applicability in the oxidative deamination reaction direction, and rivalling scopes of enzymes such as amine oxidases.^[191]^ *Asp*RedAm was combined with an NADPH‐oxidase (NOX) and ammonia borane allowing for the deracemisation of primary and secondary amines (Scheme 20B).^[192]^ Wild‐type *Asp*RedAm was used to generate (*S*)‐amines and the W210 A variant to access (*R*)‐amines. Conversions were primarily limited to 50–60 %. Further engineering of *Asp*RedAm was undertaken to unlock reductive aminations with a range of bio‐based furans coupled with aliphatic amines nucleophiles affording up to >99 % conversions (Scheme 20C).^[193]^ Mutagenesis centred on residue W210, highlighting again its importance in substrate specificity. Furfurylamines are key scaffolds in the synthesis of pharmacologically active compounds and polymers, this enzymatic route surpasses the use of toxic catalysts and chemicals. It was also noted that *Asp*RedAm displayed low ketoreductase activity, generating the corresponding alcohols from the furans.

#### 2.1.2 Further IREDs for reductive amination

**Scheme 21:**
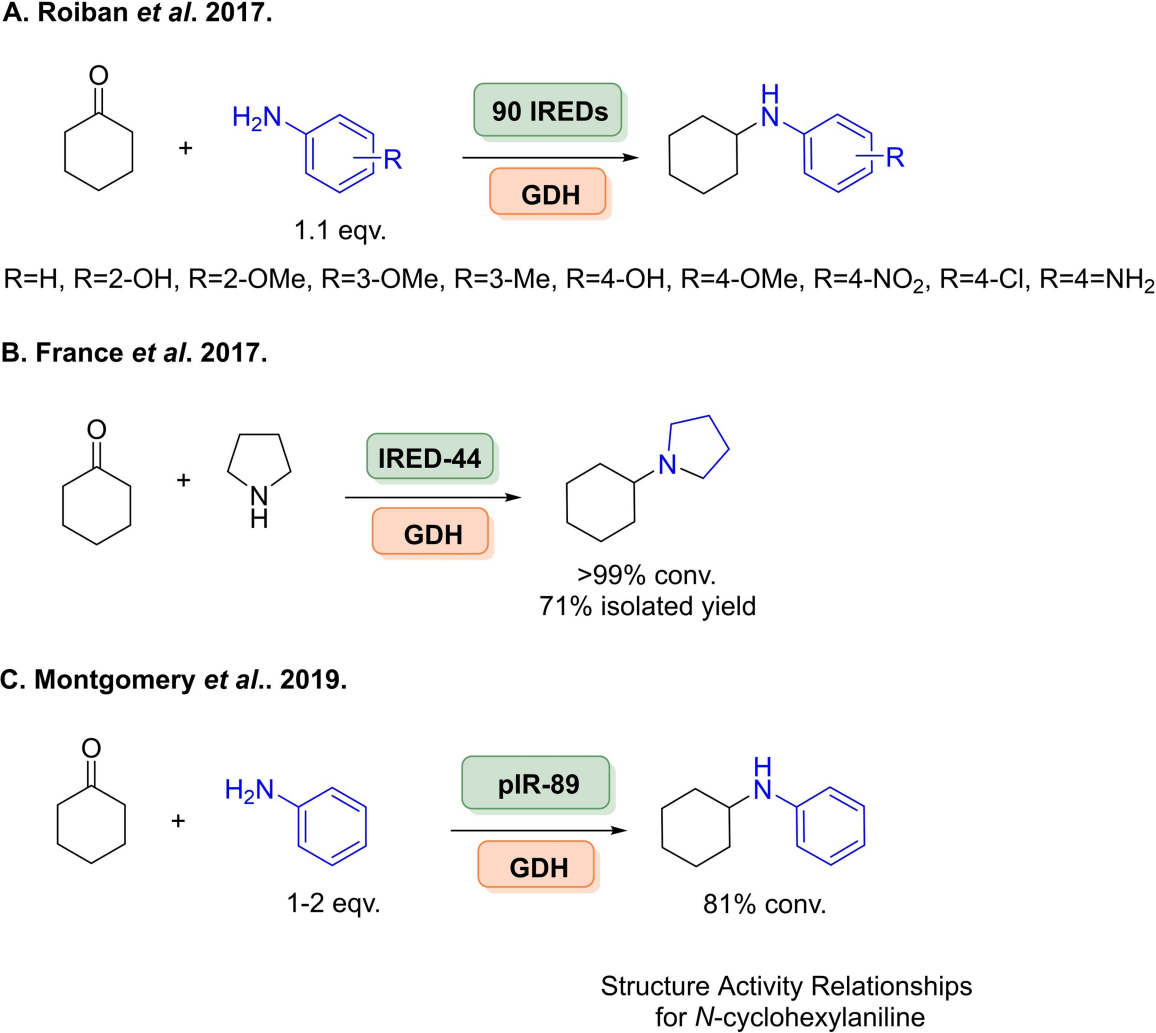
Further examples of IREDs for reductive aminations. (A) Screening of 90 genomic IREDs towards arylamine synthesis. (B) Screening of 45 bacterial genomic IREDs. (C) Screening of 90 additional genomic enzymes with aniline reaction intensifications.

The discovery of *Asp*RedAm ignited the field of IREDs for reductive amination, however studies conducted with *Asp*RedAm noted its poor stability and heterologous expression levels in *E. coli*.^[194]^ Therefore, using (meta)genome mining approaches to explore sequence space of greater diversity was carried out to overcome these issues. Researchers at GSK identified 90 bacterial IRED homologues, as well IREDs from other families implemented in natural reductive aminations such as pyrroline‐5‐carboxylate reductases,^[195]^ although little to no activity was observed with these extended families. These enzymes were screened as clarified lysates where an emphasis was placed on arylamines (Scheme 21A), particularly the weak amine nucleophile aniline, previously unaccepted by IREDs. Various functional para‐, *meta*‐ and *ortho*‐substituted anilines were screened where impressively reactions (given its nucleophilicity) employed stoichiometric quantities of aniline amine partners. Regioselectivity was also investigated with benzene‐1,4‐diamine and cyclohexanone, where GSK‐IR‐1 gave full conversion to the mono‐aminated product (Scheme 21A). The synthetic viability of this enzyme collection was demonstrated with preparative scale transformations at a 400 mg scale with yields up to 51 %.

Further bacterial sequence space was surveyed in a collaboration between Pfizer and University of Manchester, where 45 bacterial enzymes were investigated, 27 enzymes being novel (Scheme 21B).^[196]^ These enzymes were screened across a panel of ketones with methylamine, where most enzymes generated the (*S*)‐configured product. New scopes for these enzymes were investigated to demonstrate how IREDs can effectively yield tertiary amines through the coupling of cyclohexanone with pyrrolidine in 71 % yield.

**Figure 2:**
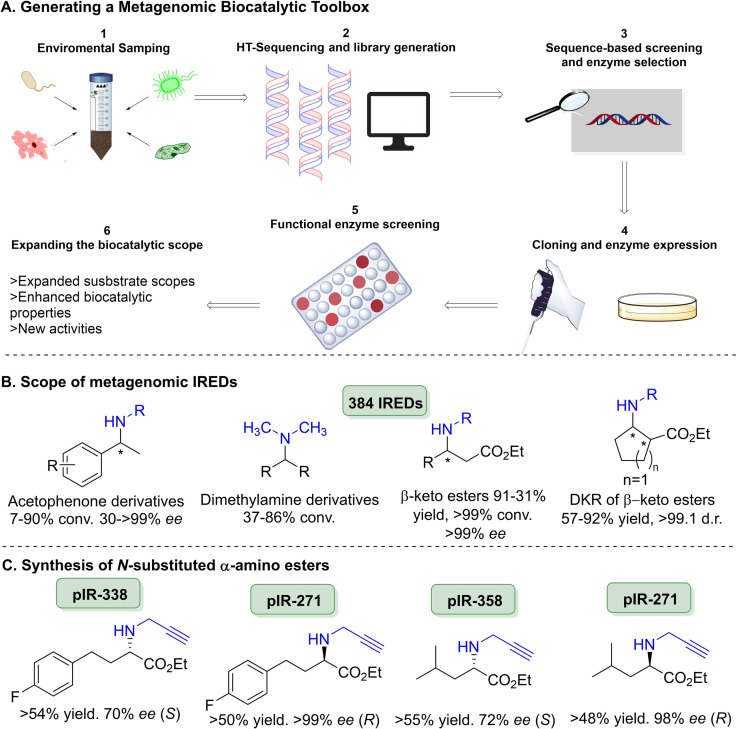
(A) Metagenomic IREDs for reductive amination. (B) Application of metagenomic IREDs, with the products of the reductive amination given. (C) Further applications of metagenomic IREDs in the synthesis of N‐substituted α‐amino acids with select products of the reactions given.

Very recently, researchers at the University of Manchester have identified considerable sizes of IRED collections, with the initial being identified by Montgomery and co‐workers in collaboration with Johnson Matthey included 90 enzymes covering many taxa across the tree of life.^[197]^ Where reaction intensifications were performed with cyclohexanone and aniline by exploring the use of co‐solvents to increase conversions without enzyme engineering (Scheme 21C). Using pIR‐89 81 % conv. was achieved with 50 mM ketone and 2 amine equiv. with 200 μL cyclohexane. Structure‐activity relationships were also performed as screening with a variety of ketone and amine substrate combinations, it was noticed that there was significant activity levels between different enzymes with high sequence identity and similar expression levels. This was then used to inform rational engineering, although this had marginal effect on the activity between the wild‐type and the variants. These 90 genomic enzymes were then blended with an additional >200 enzymes of metagenomic origin, where parental organisms are largely unknown to give an IRED toolbox of 384 enzymes.^[188]^ Generating the largest and most sequentially diverse panel of IREDs reported to date (Figure 2). By exploring metagenomics sequence space it is hoped that this would yield enzymes with greater substrate scopes, enhanced biocatalytic properties and new activities. This work was done in collaboration with Prozomix Ltd., where enzymes were arrayed in 384‐well microtitre plates for ease of enzyme accessibility and screening (Figure 2A). Coupled with this was the development of a formazan‐based colorimetric screen, a first for IREDs, which was then used characterise these enzymes across 36 diverse substrates as products of both cyclic imine reductions and reductive aminations. These enzymes were then applied to further the synthetic scope of IRED mediated reductive aminations across 3 separate avenues (Figure 2B). Where enzymes were shown to accept acetophenone derivatives (up to 99 % conv.) and dimethylamine derivatives (up to 86 %), both constituting key building blocks in a number of APIs. A new scope for IRED‐mediated reductive aminations was also demonstrated coupling β‐keto ester derivatives with a number of amine partners on preparative scale to give yields of up to 91 %, where both pairs of enantiomers could be obtained in excellent *ee* values (>99 %). This metagenomic collection was then expanded to yield a variety of *N*‐substituted α‐amino esters forming key scaffolds in a number of bioactive molecules such as *N*‐methylated analogues of peptides and peptidomimetics,^[198]^ where previous IREDs were unable to yield such products.^[187]^ These were again carried out at a preparative scale (50 mM) with >20 different transformations where separate wild type enzymes were able to afford both enantiomers for each product (Figure 2C). It was noted that depending on the substrate combinations employed, the enantioselectivity of the enzyme would invert, an observation previously noticed within this enzyme family.

**Scheme 22:**
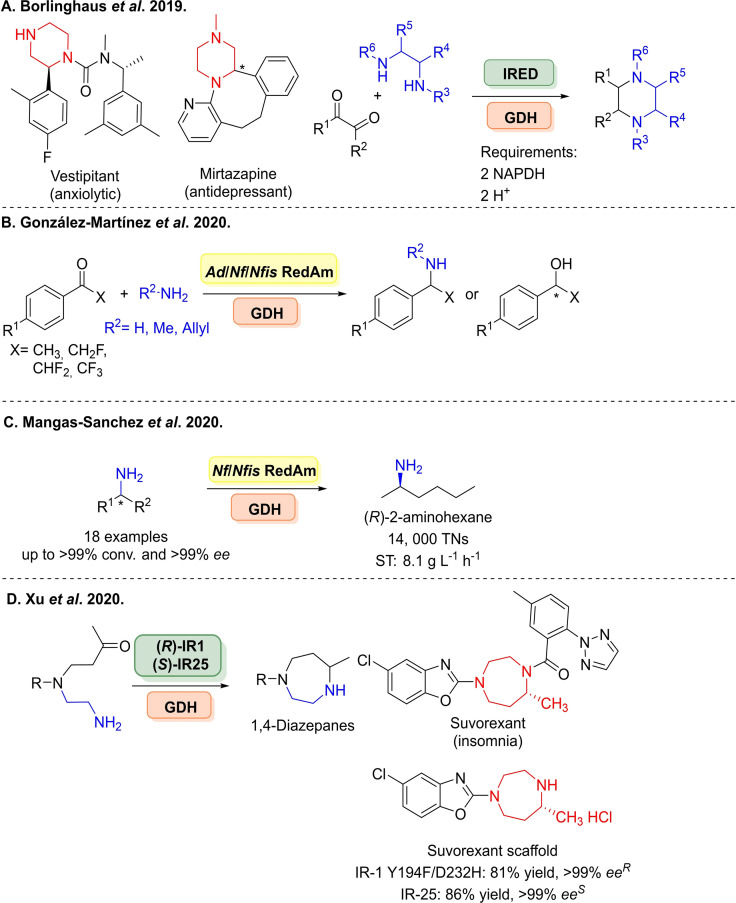
Additional examples of IRED mediated reductive aminations. (A) Synthesis of piperazines from 1,2‐carbonyl and 1,2‐diamine substrates. (B) Synthesis of β‐fluoroamines. (C) Synthesis of primary amines using thermotolerant *Nf/Nfis*RedAm. (D) Synthesis of 1,3‐diazepanes via intramolecular reductive amination with the generation of the suvorexant scaffold given.

Höhne and co‐workers also described the spectrophotometric characterisation through NAPDH consumption assays using purified IREDs from *Streptomyces tsukubaensis* and *Streptomyces ipomoeae*, Activity data was generated from a combination of 663 reductive aminations.^[199]^ However, limitations arise from requiring purified enzymes limiting its use to screen large number of variants. Furthermore, as recently shown, enzymes in this family possess ketoreductase side activity.^[193]^ Piperazine is a common building block in several pharmaceuticals such as vestipitant^[200]^ and mirtazapine.^[201]^ Interestingly, piperazine may also act as an amine nucleophile in reductive aminations. In a study by Nestl and co‐workers, piperazine derivatives were synthesised from 1,2‐carbonyl and 1,2‐diamine substrates via a double reductive amination mechanism (Scheme 22A).^[202]^ A vast range of architectures were formed, with 13 fully validated through by chromatographic analysis. Where both alkyl and aryl *N*‐ and C‐substituted products were formed, giving a diverse and novel set of API building blocks.

Recently in further work, Grogan and co‐workers identified additional fungal homologues (*Nf/Nfis*RedAm) to *Asp*RedAm which were applied to the challenging reductive amination of α‐fluoroacetophenones with ammonia (Scheme 22B), methylamine and allylamine as donors yielding β‐fluoro primary or secondary amines with >90 % conversion and 85–99 % *ee*.^[203]^ The transaminase route to such compounds is limited to 50 % due to the kinetic resolution required. It was also observed with α‐fluoroacetophenones the IRED could yield the corresponding alcohol, where control reactions determined the IREDs ketoreductase activity, a 2^nd^ observation of such activity. Additionally, these enzymes were termed reductive aminases, although a large excess of amine was used to achieve the amine product. The same 2 enzymes (*Nfis/Nf*RedAm) were also recently applied to the synthesis of a wide variety of primary amines, rivalling scopes seen with transaminases and amine dehydrogenases (Scheme 22C).^[204]^ The study by Mangas‐Sanchez demonstrated the synthetic viability of these fungal enzymes through their thermotolerance, structural properties and catalytic properties achieving 14,000 TNs and space time yields of yields 8.1 g L^−1^ h^−1^ in the generation of (*R*)‐2‐aminohexane in a continuous flow reaction system. Eighteen examples in total were given were the (*R*)‐configured product was primarily generated using ammonium chloride as the amine source.

In a first example of intramolecular reductive amination with IREDs, Yao and co‐workers carried out site‐saturation mutagenesis and generating iterative combinatorial libraries to give a 61‐fold improvement towards intramolecular reductive aminations accessing 1,4‐diazepane products employing IR‐1‐Y194F/D232H to yields the (*R*)‐configured amines and wild‐type IR‐25 to give the (*S*)‐enantiomers (Scheme 22D).^[205]^ 1,3‐Diazepanes are found in number of APIs including ripasudil, fasudil and survexetant. The latter compound the substructure was synthesised with an impressive 81 % yield and >99 % *ee*, along with 10 other preparative scale transformations.

#### 2.1.3 IREDs operating at industrial scale

**Scheme 23:**
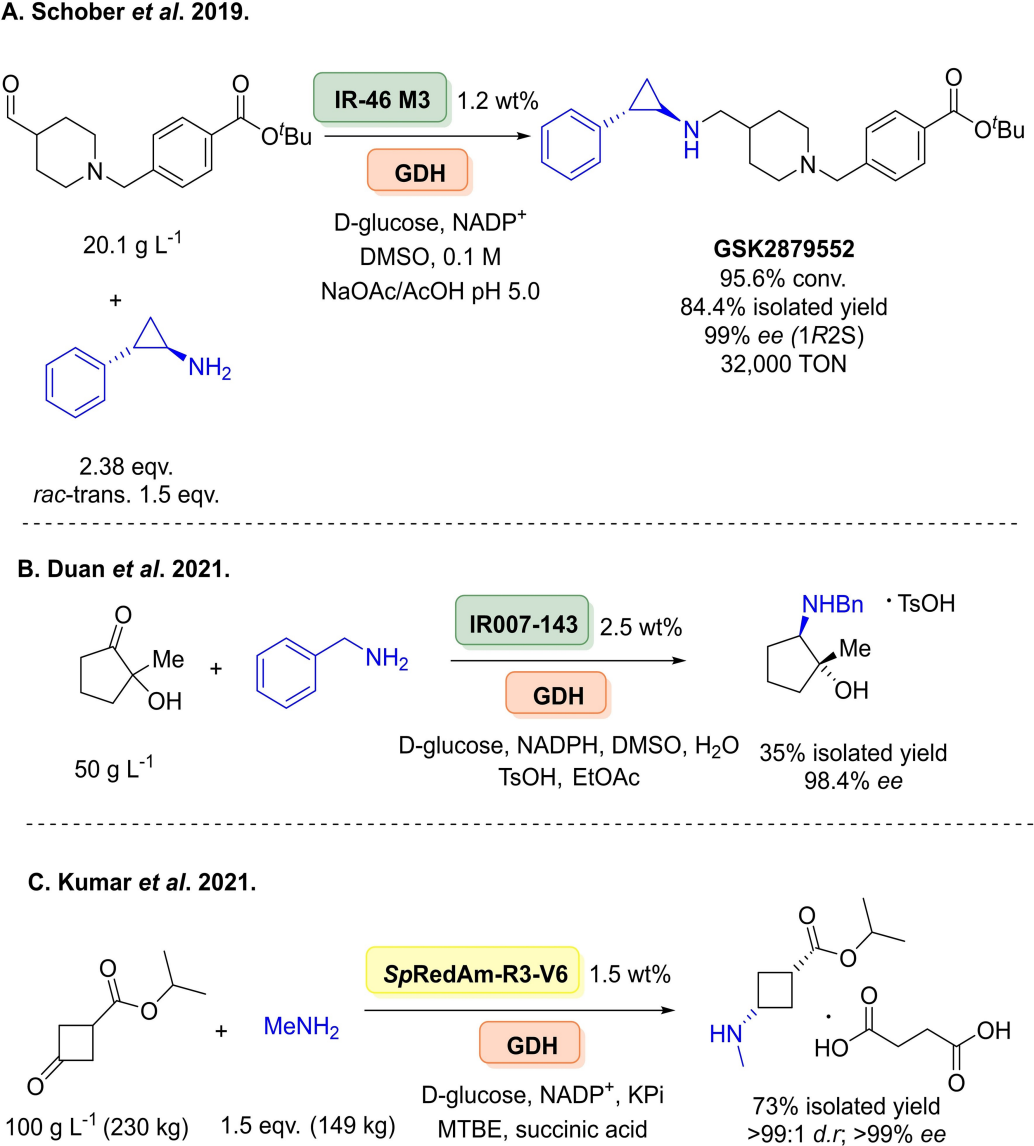
IREDs operating at industrial scale. (A) Schober and co‐workers for the directed evolution IR‐46 in the synthesis of a lysine‐specific demethylase‐1 inhibitor, GSK2879552.

A further appeal of this enzyme family is their ability to readily operate at industrial scale. Researchers at GSK implemented a robotics screening platform in the directed evolution of an IRED for the asymmetric reductive amination and concomitant kinetic resolution towards a lysine‐specific demethylase‐1 inhibitor, GSK2879552 at the time in phase II clinical trials for small cell lung cancer and acute leukaemia (Scheme 23A).^[206]^ Chemical approaches in the synthesis of the target require, **1**. the classical resolution of the amine donor, **2**. a low temperature reductive amination and **3**. subsequent multiple work‐up procedures requiring vast amounts of solvents. Initial screening of their collection of 90 enzymes identified GSKIR‐46, where a 43 % yield of the product was obtained on a 5 g scale. Over 3 rounds of directed evolution, screening nearly 10,000 variants, a variant M3 (Y142S; L37Q, A187V, L201F, V215I, Q231F, S258N; G44R, V92K, F97V, L198M, T260C, A303D) was yielded with an >38,000‐fold improvement over the wild‐type, where the enzyme was engineered towards several factors including increased substrate and biocatalyst loading, stereoselectivity and yield. The enzyme was also engineered towards operating under acidic conditions to aid substrate solubility, where previous reports had only described IREDs operating under neutral to basic conditions. Both residues L198M and L201F were identified as residues to interact with substrate stabilisation at the dimer interface. The group also noted initially library design was a challenge due to the nature of IREDs dynamic mechanism and few mutagenesis studies. M3 was then used for the reductive amination on a kilogram scale giving 84.4 % isolated yield and 99.7 % *ee*. They also investigated the use hydrogen‐borrowing cascade starting from the alcohol via the *in situ* aldehyde, although this was not viable due to reaction equilibrium issues and requiring additional amine loadings. Very recently, researchers at Pfizer have reported two large‐scale processes for the preparation of APIs involving RedAms. Firstly, they investigated the use of a RedAm for the synthesis of an amino alcohol intermediate in the synthesis of a cyclin‐dependent kinase (CDK) 2/3/4 inhibitor (Scheme 23B).^[207]^ The initial process involved a transaminase‐mediated reductive amination although issues concerning product isolation prompted the authors to reconsider the route by using an IRED with benzylamine as coupling partner. After initial screening, a wild‐type enzymes IR007 was used as a template for evolution in efforts to improve activity and stability. The final variant IR007‐143 was able to convert the substrate in 43 % conversion and in 98 % *ee* with a substrate concentration of 50 g L^−1^. They have also followed the same approach to design a novel process towards the synthesis of the Janus kinase 1 (JAK1) inhibitor abrocitinib (Scheme 23C).^[208]^ After initial screening, the RedAm from *Streptomyces purpureus* (*Sp*RedAm) proved to be the best candidate for evolution. Initially, 93 positions were selected for single site saturation mutagenesis, followed by recombination of beneficial mutations and further recombination under final process conditions, yielded a variant *Sp*RedAm−R3‐V6 able to achieve the desired diastereomer in 92.5 % conversion, >99 % *d.r*., and 73 % isolated yield at a 230 kg scale (100 g L^−1^ concentration) using 1.5 equivalents of methylamine.

Furthermore, researchers at Johnson Matthey also highlighted the viability of IREDs for reductive amination at scale.^[209]^ Bornadel and co‐workers described the necessary considerations for scaling up and IRED reductive amination between cyclohexanone and cyclopropylamine. Given the nature of reductive amination, as it is multi‐factorial reaction, design of experiment (DoE) was implemented studying different reaction conditions to give the optimal parameters. DoE identified the most influential parameters to be ketone and amine concentration, temperature and IRED loading. When the reaction was moved from batch to fed‐batch of amine and ketone this allowed for a successful scale up of the reaction. A 90 % isolated yield was achieved with 750 mM ketone concentration, with turnover numbers of 48,0000.

Finally, researchers at Novartis have recently used the IRED‐mediated reductive amination reaction to access the H4 receptor antagonist ZPL389 to compare machine‐learning guided directed evolution with classical approaches such as deep mutational scanning (DMS) and error‐prone PCR (epPCR).^[210]^ The best performing variant obtained via DMS and epPCR was used at a gram scale obtaining the desired product in 72 % isolated yield and >99 % *ee*.

#### 2.1.4 IREDs in flow biocatalysis

An emerging and promising area of biocatalysis is the use of flow biocatalysis where enzymes are (co‐)immobilised through various approaches to support resins. This approach offers to improve the efficiency of biocatalytic reactions through improved enzyme stability, enzyme recyclability, improves scalability and overcomes previously incompatible cascades.

The first example of this technique was given in 2019, as previously mentioned *Asp*RedAm displays poor stability. In an effort to improve this, *Asp*RedAm was immobilised on EnginZyme's EziG^™^’s Amber support where *Asp*RedAm was studied for the reductive coupling of cyclohexanone with allylamine, where activity was significantly higher for the co‐immobilised 34 % soluble enzyme vs 90 % immobilised.^[194]^ As IRED mediated reductive amination in a multi‐enzyme system, optimisation where multiple enzymes are co‐immobilised is a challenge. As such a DoE approach was taken to improve immobilisation of enzymes for reductive amination, where a computer aided work‐flow was used to mechanistically and empirically model predictions for optimal space‐time yields with minimal cofactor and co‐substrate requirement.^[211]^ Hydrocinnamaldehyde and allyamine was used as the model reaction. All factors of the IRED reaction were studied, where testing all combinations of factors would have required 729 experimental runs, instead through DoE only 17 runs were needed from the empirical modelling. Two preparative scale transformations were performed with one being a cost‐effective strategy and another to maximize space time yields at all costs, where both methods achieved expected and good results.

**Scheme 24:**
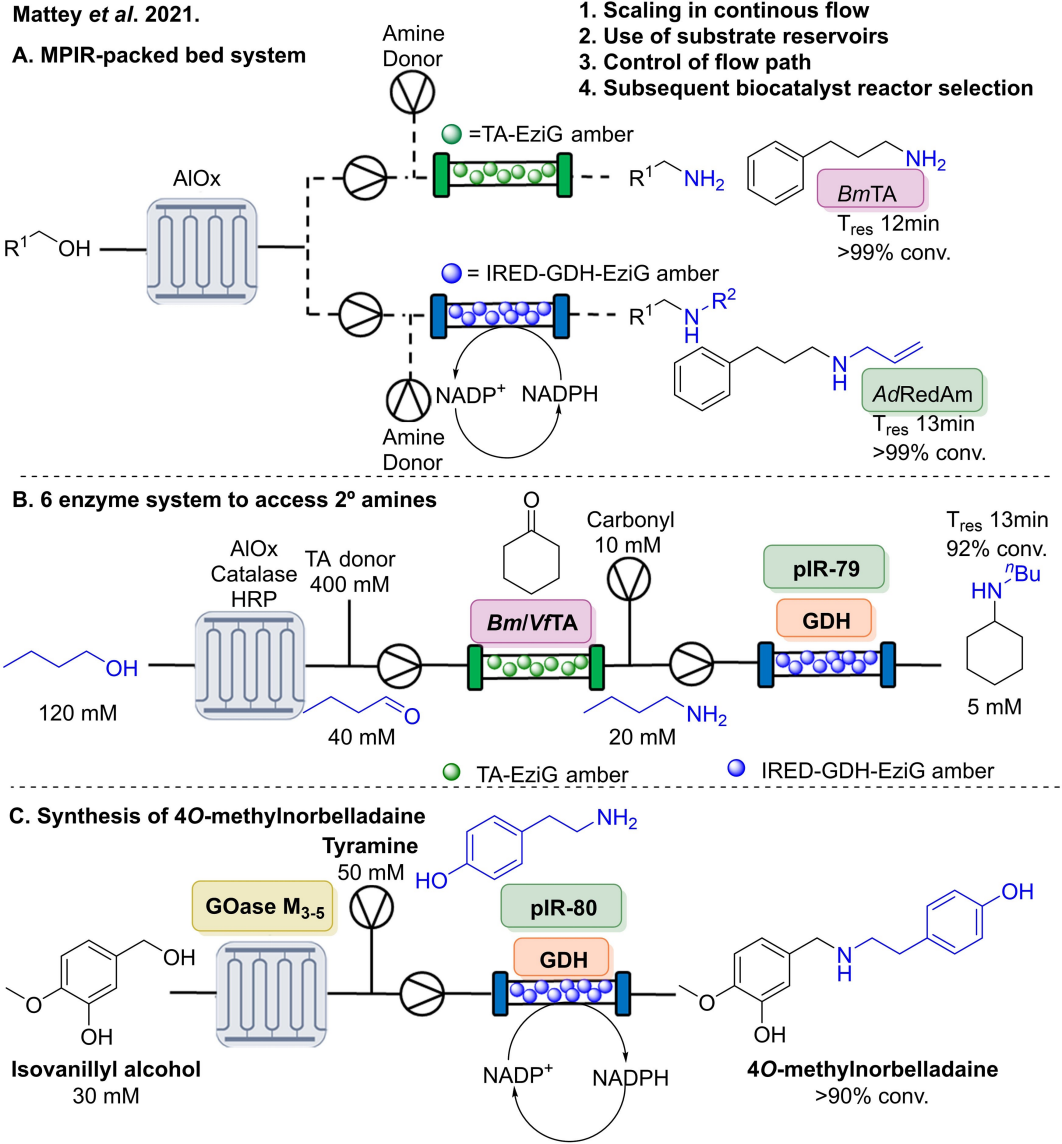
IREDs in flow. (A) An overview of the MPIR‐packed bed system with the AlOx (alcohol oxidase) in cascade with the either a transaminase (TA) or IRED. (B) A 6‐enzyme compartmentalised system to access secondary amines. (C) Combing GOase M_3‐5_ with pIR‐80 to access 4*O*‐methylnorbelladaine from isovanillyl alcohol and tyramine.

Addressing previously incompatible cascades by taking a reaction compartmentalisation approach and using switching valves to alter the direction of the intermediate products towards different downstream enzymes. Mattey and co‐workers immobilised different alcohol oxidases to generate *in situ* aldehydes to be then fed to a number of aminating enzymes including IREDs and RedAms, where transaminases were also employed to also give the analogous primary amine (Scheme 24A).^[212]^ Initially a range of benzyl alcohol derivatives were studied when galactose oxidase (GOase) variant M_3‐5_ was combined with either ω‐TA or an IRED to give primary and secondary amines, respectively. Typically, in the presence of amines galactose oxidase's copper centre is inactivated. Furthering this approach, cross reactivity between IREDs for reductive amination and ω‐TA's can be an issue in a ‘one‐pot’ process. To overcome this limitation, a TA‐IRED cascade was constructed in compartmentalised approach where pIR‐79 combined *Bm*TA gave *N*‐butylcyclohexylamine from butanal in 85 % conversion via butylamine generated from *Bm*TA. It also seemed plausible to extend this system to start from the primary alcohol, where a 6‐enzyme system was used to generate the final secondary amine with 92 % steady state conv. (Scheme 24B). Finally, screening the Turner/Prozomix metagenomic IRED collection identified pIR‐80 active towards the natural product 4*O*‐methylnorbelladine, a scaffold of the dementia API galantamine. pIR‐80 was combined with GOase M_3‐5_ to generate isovanilin which was reductively coupled by the IRED with tyramine to generate 4*O*‐methylnorbelladaine (Scheme 24C), due to its insolubility this allowed for a facile product isolation. The application of IREDs for reductive amination in flow has allowed for greater synthetic versatility of these enzymes.

#### 2.1.5 IREDs in cascades

Multi‐enzymes biocatalytic cascades are one of key steps towards delivering more renewable synthesis of chemicals. Cascades provide the convenient synthesis of many high‐value materials starting from inexpensive starting materials. Cascades also circumvent having to isolate challenging intermediate materials thus reducing time and waste from work‐up materials.

**Scheme 25:**
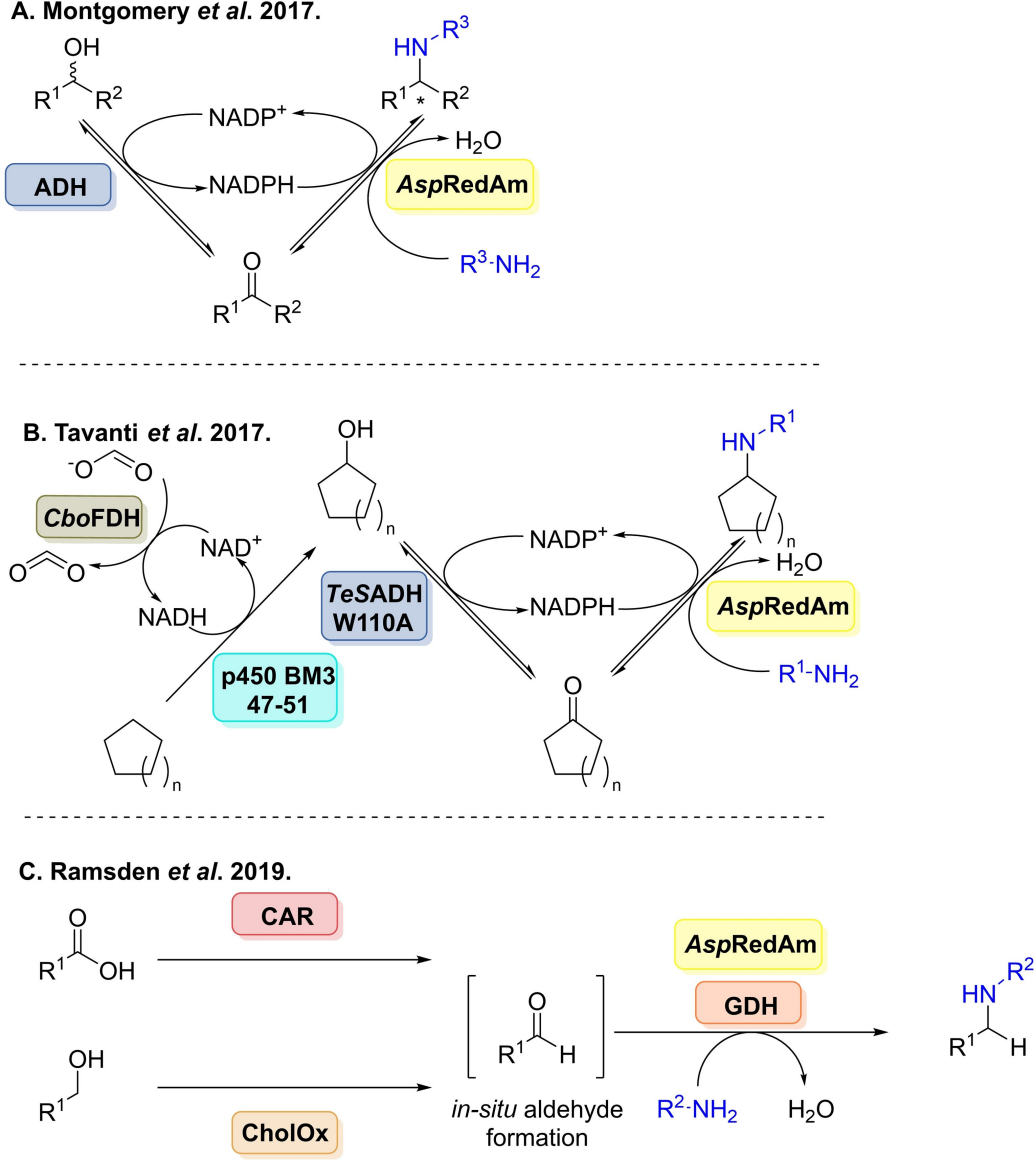
IREDs in cascades. (A) *Asp*RedAm implemented in hydrogen‐borrowing cascades for the alkylation of amines, ADH=alcohol dehydrogenase. (B) Extending the hydrogen borrowing cascade to unfunctionalised cycloalkanes by employing a P450 BM3 variant. (C) *N*‐alkylation of primary amines with primary alcohols or primary carboxylic acids, CholOx=choline oxidase.

In 2017 Montgomery and co‐workers substituted an AmDH for *Asp*RedAm in the direct alkylation of amines with alcohols in a hydrogen borrowing cascade combined with a non‐selective alcohol dehydrogenase (ADH) (Scheme 25A).^[213]^ Where the IRED is advantageous over the AmDH in being able to generate both secondary and tertiary amines. This cascade is also redox neutral generating water as the sole by‐product in a first example of biocatalytic hydrogen autotransfer for the direct preparation of secondary amines. A range of secondary amines were generated in excellent yields and selectivities. This cascade was then extrapolated further starting from unfunctionalised cycloalkanes employing a P450 BM_3_ variant to carry out the hydroxylation (Scheme 25B).^[214]^

The hydrogen borrowing approach although redox neutral inherently suffers from issues around reaction equilibria. Ramsden and co‐workers offered an alternative to this through by combining *Asp*RedAm with an alcohol oxidase or carboxylic acid reductase (CAR) starting from the alcohol or carboxylic acid, respectively (Scheme 25C).^[215]^ Both enzymes due to the nature of their mechanism are irreversible in their reactions, overcoming equilibrium issues. The cascade generated a plethora of secondary aliphatic and aryl amines. Although this approach is limited to the *in situ* aldehyde formation due to primary enzymes of the cascade.

### 2.2 Amine dehydrogenases (AmDHs) for reductive aminations

Amine dehydrogenases (AmDHs) are another synthetically relevant class of oxidoreductases that can carry out asymmetric reductive aminations of carbonyls with inexpensive ammonium salts to generate the corresponding (chiral) primary amines. These enzymes are typically coupled with a formate dehydrogenase cofactor recycling system where ammonium formate can be used as the amine and reductant source, favouring atom economy. Initially AmDHs were evolved from amino acid dehydrogenases (AADHs) through point mutations directing the substrate specificity away fr6 m amino acid acceptance. These reports have been extensively reviewed by Sharma *et al*.^[215]^ and Patil *et al*.^[217]^ In this survey we focus on later reports on the recent synthetic applications of engineered AADHs and discovery of wild‐type AmDHs.

#### 2.2.1 Substrate scopes of engineered AmDHs from AADHs

The first AmDH variants were generated by the Bommarius group via active site engineering of the leucine dehydrogenase from *Bacillus stearothermophilus* (L‐AmDH), the phenylalanine dehydrogenase PheDH from *Bacillus badius* (F‐AmDH), and a chimeric AmDH^[218]^ (Ch1‐AmDH) generated by combination of the *N*‐terminus from F‐AmDH,^[219]^ and residues 146–160 from L‐AmDH.^[220]^

Investigating the scope of F‐AmDHs in a collaboration between the Li group and GSK,^[221]^ directed evolution was carried out on PheDH from *Rhodococcus* where NNK libraries of residues K66 and N262 of the carboxylate binding pocket were generated, where the mutant N66Q/N262C (TM_pheDH) was able to aminate 4‐phenyl‐2‐butanone and phenylacetone, a moiety of the API selegiline. Expansion of the binding pocket by 2 Å allowed greater substrate accommodation. The variant was able to generate the enantiopure product from phenylacetone in 98 % *ee*. Furthermore, preparative scale reactions of 4‐phenyl‐2‐butanone were shown on a 15 mM scale giving 95 % conv. and 2800 turnover numbers with respect to NAD^+^. Although substrate scope remained narrow.

**Scheme 26:**
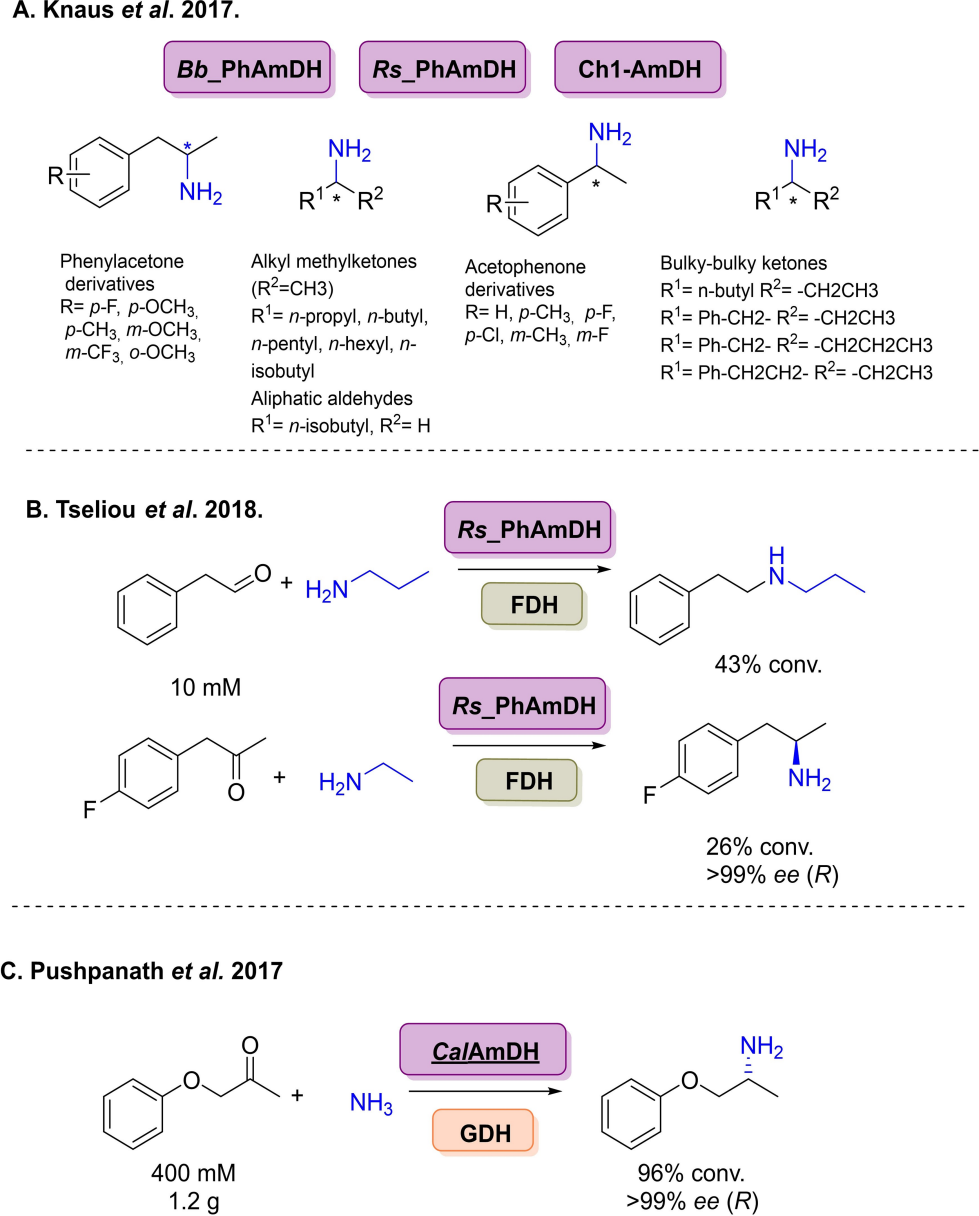
Scope of phenylalanine and chimeric AmDHs across different substrate scopes. (A) An investigation into scope of Bb_PhAmDH, Rs_PhAmDH, and Ch1‐AmDH across a variety carbonyl classes as given, with also promiscuous carbonyl reductase activity. (B) Exploring synthesis of secondary amines with AmDHs, where side activity of the primary amine synthesis was observed. (C) Exploring industrial applicability of AmDH reactions employing 400 mM substrate loadings.

In 2017 Mutti and co‐workers^[222]^ undertook a comprehensive study on the carbonyl scope and catalyst parameters of several AmDHs, including phenylalanine dehydrogenase from *Bacillus badius* (*Bb*‐PhAmDH), phenylalanine dehydrogenase from *Rhodococcus* sp. M4 (*Rs*‐PhAmDH) and the chimeric AmDH, Ch1‐AmDH. The co‐factor recycling system was identified to be a formate dehydrogenase/ammonium buffer system, where reactions went to completion in 5 h with good activity up to 50 °C, where previously in the field reactions had proceeded quite slowly. A broad carbonyl scope was investigated across all three AmDHs at 50 mM including phenylacetone derivatives, alkyl methylketones, acetophenone derivatives, and sterically demanding ketones (Scheme 26A). Ch1‐AmDH displayed good activity towards phenylacetone derivatives, whereas previously *Rs*‐PhAmDH, had been developed and tested only for the reductive amination with 4‐phenylbutan‐2‐one, overall this enzyme had a greater than expected substrate scope. Aliphatic ketones were accepted with examples up to 2‐octanone, where Ch1‐AmDH displayed the greatest activity. Interestingly the group also screened bulky‐bulky ketones bearing the carbonyl moiety conjugated with the phenyl ring, for example 1‐phenylpropan‐1‐one, which are typically challenging substrates for enzymes such as ω‐TAs. *Rs*‐PhAmDH was the optimal performing enzyme giving >99 % conv. and >99 % *ee*‐(*R*). It was also noted that when assaying hydrocinnamaldehyde with Ch1‐AmDH, the corresponding alcohol was obtained in 30 % conv. Generally, *Rs*‐PhAmDH was superior to *Bb*‐PhAmDH.

In further work by Mutti and co‐workers utilising *Rs*‐PhAmDH and Ch1‐AmDH,^[223]^ their scopes were studied for the acceptance of primary and secondary amine nucleophiles, like that seen with *Asp*RedAm. *Rs*‐PhAmDH gave up to 43 % conv. coupling phenylacetaldehyde and propylamine, generally primary amines were more successful but secondary amines were poorly accepted. Additionally, chemoselectivity was a challenge where the AmDHs generated a mixture of primary and secondary amine products (where the secondary amine product was desired), postulated to operate through a transamination mechanism (Scheme 26B). This highlights inherent issues associated with the amine nucleophile scope of AmDHs.

Workers at Johnson Mattey wanted to overcome limitations associated with *Bb*‐PhAmDH and AmDHs in general, targeting improving the general industrial applicability, and did so by comparing *Bb*‐PhAmDH to the newly generated phenylalanine dehydrogenase from *Caldalkalibacillus thermarum* (*Cal*AmDH).^[224]^ Employing *Cal*AmDH, substrate loadings of up to 400 mM were achieved with phenoxy‐2‐propanone (Scheme 26C), where high biocatalyst loadings were used. Although k_cat_ values remained low, and substrate tolerance was not great overall, this was hypothesised from a lack of stabilisation residues within the carboxylate binding pocket.

Understanding the scope of engineered L‐AmDHs, Xu and co‐workers in 2015 targeted three leucine dehydrogenases for structure‐guided evolution to infer AmDH activity.^[225]^ A homology model was generated from the leucine dehydrogenase from *Bacillus sphaericus* with the leucine dehydrogenase from *Lysinibacillus fusiformis*. The author's rationalised certain residues could be targeted to enlarge the active site. Alanine scanning generated a variant *Lf*AmDH‐M2 with greater activity towards aminating 2‐hexanone. Further mutagenesis of A113G/T134G gave a crucial enlargement of the active site generating the variant *Lf*AmDH‐M3, capable of accepting ketones of 10 carbons in chain length. This mutagenesis approach was then translated to 2 other AmDHs, *Es*AmDH from *Exiguobacterium sibiricum*, and *Bsp*AmDH from *Bacillus sphaericus*.

The Gröger group utilised the L‐AmDH from *Exigobacterium sibiricum* (*Es*LeuDH‐DM) for further engineering towards aryl‐substituted ketones.^[226]^ Acetophenone was chosen as the model substrate, where optimal activity was demonstrated, *Es*LeuDH‐DM also showed higher activity towards substrates with electron‐withdrawing substituents such as 4‐nitroacetophenone. A preparative‐scale example was given at 50 mM scale with GDH for co‐factor recycling, excellent selectivity was achieved (>99 % *ee*). Although the reaction was left to proceed for a lengthy 100 h. Furthermore, the group also studied the immobilisation of *Es*LeuDH‐DM where a hydrophobic carrier was the optimum studied allowing for a high amount of protein to be immobilised at 63.2mg/g of support. Identifying further beneficial sets of mutations, the LeuDH from *G. stearothermophilus* was mutated to generate L‐AmDH‐TV (D32A, F101S, and C290V), a variant which was able to accept branched aliphatic ketones.^[227]^ Further mutagenesis of L39A, A112G and T133G increased substrate pocket expansion for long chain aliphatic ketones.

**Scheme 27:**
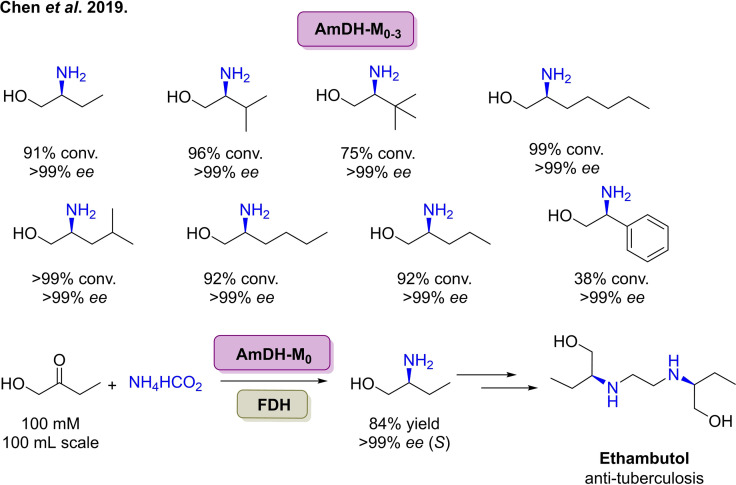
Synthesis of α‐hydroxyketones with AmDH variants of LeuDH from *Lysinibacillus fusiformis* with also the formal synthesis of Ethambutol.

The chiral vicinal amino alcohol is a key motif in several bioactive compounds, although chemical approaches to these methods require harsh reaction conditions and rare transition metal catalysts. In a 2019 study, the LeuDH from *Lysinibacillus fusiformis* was engineered to AmDH_M0‐4_ testing a panel of α‐hydroxyketones (Scheme 27).^[228]^ AmDH‐M_0_ exhibited broadest range, but AmDH‐M_3_ accepted bulkier aliphatic hydroxyketones. This system could be used in the synthesis of the anti‐tuberculosis agent, ethambutol. Using AmDH‐M_0_, the corresponding (*S*)‐product (forming the scaffold of ethambutol) could be synthesised on a 100 mM scale with 84 % yield and >99 % *ee* (*S*), then achieving final synthesis of the (*S*,*S*)‐API. Exploring similar α‐hydroxyketone scopes, 6 AADHS of both leucine and phenylalanine classes were cloned, expressed, and engineered to AmDHs.^[229]^

**Scheme 28:**
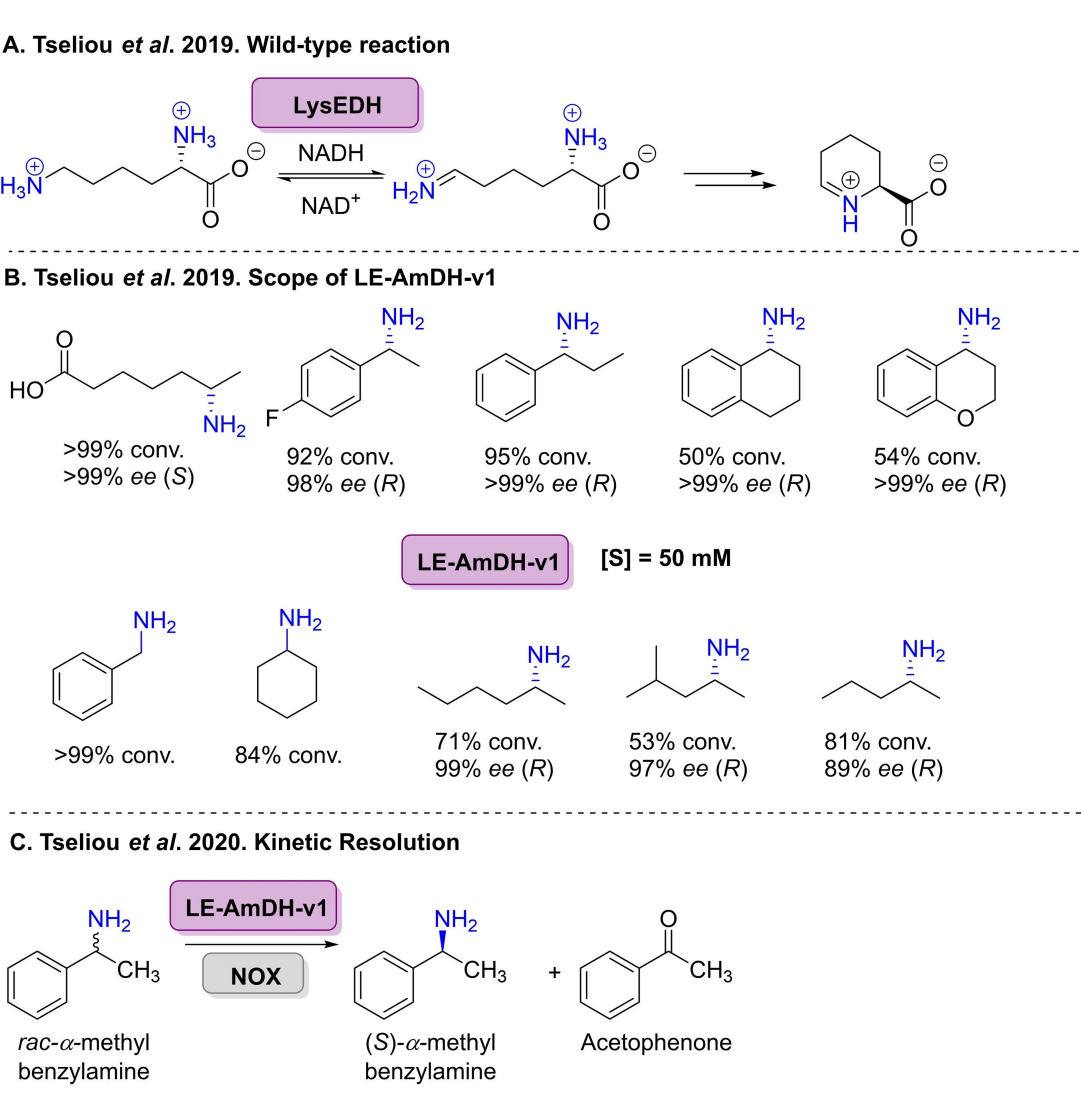
The scope and applications of the engineered ϵ‐deaminating L‐lysine dehydrogenase from *Geobacillus stearothermophilus*. (A) The wild‐type reaction is outlined. (B) Examples of the scope and selectivity of the LE‐AmDH‐v1 variant at 50 mM substrate concentration. (C) Application LE‐AmDH‐v1 combined with a NOX to allow of the kinetic resolution of primary amines, where the scope was similar to that shown in B.

Until this point the general scope of amine dehydrogenases had been quite narrow focusing on largely phenyl‐derived ketones, where AmDHs had been evolved from L‐AADHs targeting specific residues. Examining a new family of AADHs, Tseliou and co‐workers in 2019 generated an AmDH from an ϵ‐deaminating L‐lysine dehydrogenase from *Geobacillus stearothermophilus*, where the wild‐type reaction is highlighted in Scheme 28A.^[230]^ Initially, it was determined that the α‐carboxyl group is essential for substrate binding but not the α‐amino group. Homology modelling identified 2 classes of residues, 1. residues necessary for binding of the α‐amino/carboxylate (forming a hydrophilic cavity) and a 2^nd^ group of hydrophobic resides forcing the substrate into the reactive pose. 14 variants were screened with tetralone and acetophenone as the model substrates identifying the optimal variant LE‐AmDH‐v1 (Scheme 28B). A variety of ketone and aldehyde substrates were screened with this variant where excellent activity (up to >99 % conv) was seen with hindered acetophenone derivatives. α‐Tetralone and α‐chromanone derivatives were also well accepted at 50 mM substrate loadings, previously not tolerated with engineered AmDHs. Reaction intensifications up to 100 mM were conducted with 86 % conv. The catalytic performance of LE‐AmDH‐v1 was shown to be 15 times more efficient than Ch1‐AmDH, where *in silico* studies complemented the mutagenesis identifying key roles of mutations. Harnessing this enzyme's catalytic efficiency, LE‐AmDH‐v1 was then combined with a NOX for the kinetic resolution of primary amines to generate (*S*)‐configured amines.^[231]^ *rac*‐α‐Methylbenzylamine was used as the model substrate, however only aryl‐substituted amines could be accepted, aliphatic amines were not tolerated.

The tolerance of F‐AmDHs towards generating secondary amines was also explored through the engineering of a thermostable PheDH from *Geobacillus kaustophilus* to generate *Gk*AmDH.^[232]^ The variant was screened across a diverse set of ketones namely *para*‐, *ortho*‐ and *meta*‐phenylacetone derivatives and aliphatic ketones from 2‐hexanone to 2‐octanone. Interestingly, the group explored the synthesis of chiral secondary amines with *Gk*AmDH, use of methyl‐, ethyl‐, cyclopropyl‐, and allylamine. Good conversions were obtained with methyl‐ and cyclopropylamine 97 and 88 %, respectively, demonstrated also through a series of preparative‐scale transformations. Recently, the chimeric AmDH, Ch1‐AmDH was immobilised onto a continuous flow pack‐bead reactor system in the amination of 5‐methyl‐2‐hexanone coupled with an FDH.^[233]^ Both enzymes were co‐immobilized onto the Nuvia® IMAC resin from Bio‐Rad, with conversions up to 48 % and volumetric productivities up to 443 g L^−1^ day^−1^, highlighting the use of flow biocatalysis in the field of AmDHs.

#### 2.2.2 Naturally occurring AmDHs

**Scheme 29:**
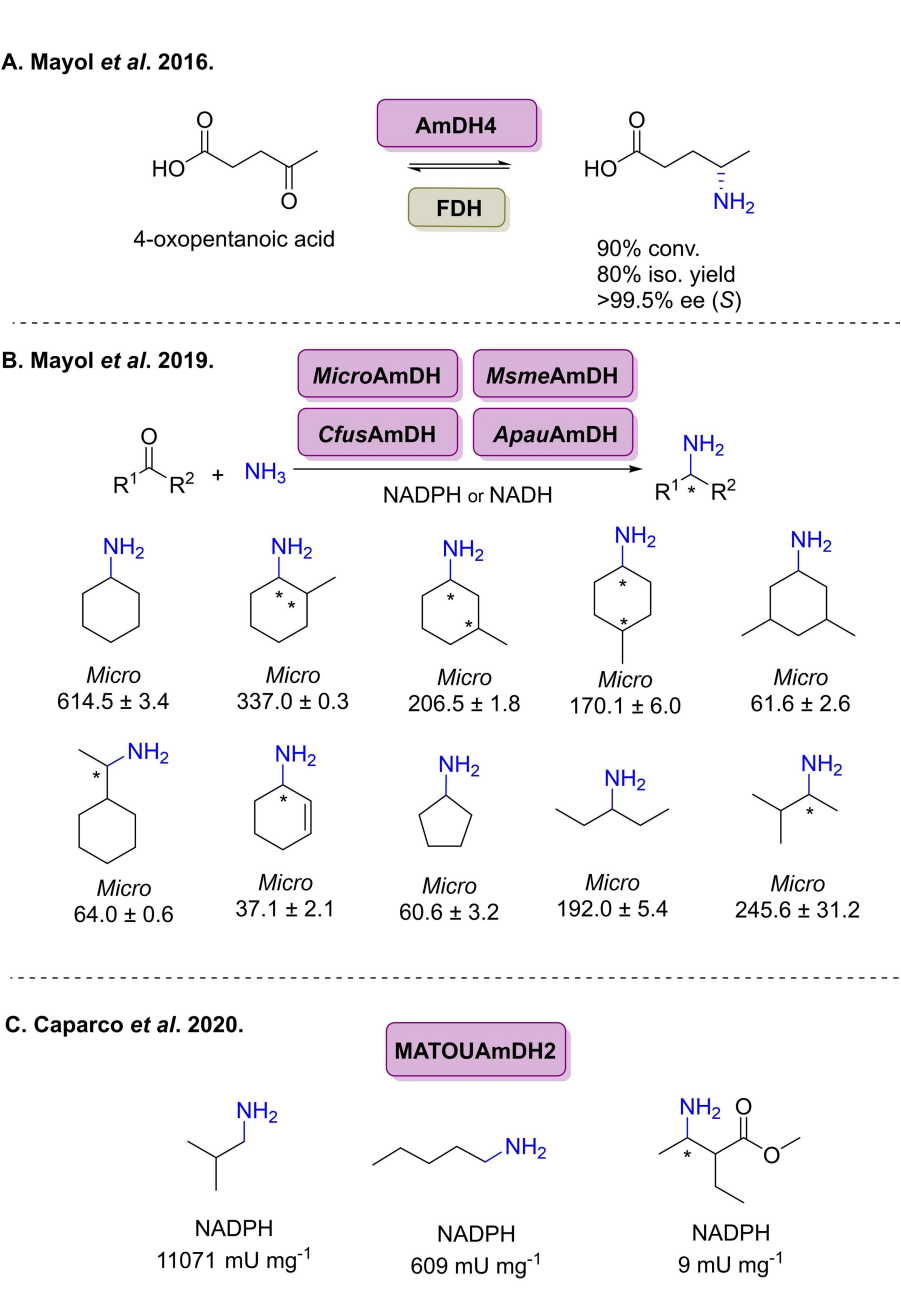
The scope and applications of natural and metagenomic AmDHs. (A) First example of wild‐type AmDHs, AmDH4 in the reductive amination of 4‐oxopentanoic acid. (B) Exploration of wild‐type genomic AmDHs with the enzyme studied listed in the table with example of the scope and specific activity mU mg^−1^ given below each product. (C) Exploration of metagenomics resources for the discovery of new AmDHs.

The very first naturally occurring AmDH was reported in 2000 by Itoh and co‐workers from *Streptomyces virginae* IFO 128. The enzyme was purified and screened *in‐vitro*, although no sequence information was disclosed hindering further enzyme discovery.^[234]^ Further work towards the discovery of natural AmDHs was made by Vergne‐Vaxelaire and co‐workers in 2016 who performed a search on dehydrogenase homologues excluding those that aminate α/β‐carboxylate groups.^[235]^ Using the L‐erytho‐3,5‐diamionohexanoate dehydrogenase sequence the group identified 169 homologous sequences, 20 were cloned and expressed. Characterisation of these enzymes identified AmDH‐4 able to reductively aminate 4‐oxopentanoic acid to generate (*S*)‐4‐aminopentaoic acid in 80 % isolated yield (Scheme 29A). Although the carboxylate group was still a pre‐requisite for activity, further sequence exploration and chemical space exploration was required.

In a further advancement towards natural AmDHs with broader substrate scopes, Vergne‐Vaxelaire and co‐workers utilised AmDH‐4 as a seed sequence for further natural AmDH discovery in 2019.^[236]^ This sequence was chosen as it shared low sequence similarity to AADHs, IREDs or RedAms. 23 enzymes were identified and subsequently screened in cell‐free extract (CFE) format (Scheme 29B). One enzyme, *Msme*AmDH displayed activity in the reductive amination for cyclohexanone (a previously poor AmDH substrate), but displayed no activity towards (2*R*,4*S*)‐2,4‐diaminopentanoate or 4‐aminopentanoate. Further enzyme discovery was undertaken around *Msme*AmDH, identifying 3 homologues *Cfus*AmDH, *Micro*AmDH and *Apau*AmDH. Where biochemical characterisation identified these enzymes to be homodimeric with a preference towards NADPH. These newly identified enzymes were then compared to AADHs, engineered to AmDH and IREDs through a sequence similarity network (SSN) with >5,000 homologous nodes in the network. This highlighted these newly discovered enzymes are evolutionarily distinct from previously identified AmDHs. In the SSN approximately 70 % of the enzymes were annotated as dihydropiconilate reductases, an asymmetric imine reducing enzyme class. Structurally, these enzymes also shared a high resemblance to the dihydropicolinate reductase family, confirmed by the homodimeric nature and conserved Rossmann‐fold. Kinetics coupled with point mutagenesis gave a proposed mechanism, like previously reported AmDH reductive aminations.

The scope of these enzymes was also challenged across a broad range of ketones including cycloalkanones, aliphatic aldehydes, branched acyclic ketones where these substrates were well accepted. However, little activity was detected with aromatic substrates such as phenylacetone derivatives, typically well‐tolerated with engineered AmDHs. Methylamine and ammonia were both well‐tolerated as amine donors, and importantly no conversion to the corresponding alcohol was observed. Engineering of AmDH4 was also undertaken towards the acceptance of 2‐pentanone where specific activities of 104.8 mU mg^−1^ were obtained. Overall, these genomically mined AmDHs offer new scopes for the AmDH family being able to tolerate substrates lacking aromaticity.

As previously discussed, metagenomics is an emerging and fruitful platform for the identification of novel enzymes. In further work towards expanding the area of wild‐type AmDHs in a collaboration between the Vergne‐Vaxelaire and the Bommarius labs, oceanic (a rich source of enzymes) and human microbiomes were screened, identifying 18 metagenomic AmDHs, where a further subset of 6 enzymes were taken forward for reductive aminations.^[237]^ This represents the first AmDH to be of eukaryotic origin. The enzyme MATOUAmDH2 was shown to be the most promising candidate, with good activity towards aliphatic aldehydes such as isobutyraldehyde and pentaldehyde, previously unattainable with natural AmDHs (Scheme 29C). MATOUAmDH2 was engineered to accept bulkier ketones and γ‐ketoesters such as methyl 2‐ethylacetoacetate. This represented a nice diversification for the AmDH family, where the catalytic efficiency of this enzyme was shown to be high, rendering it suitable to hydrogen‐borrowing cascades. Although further engineering would be required to access more aromatic substrates. Finally, engineering of a wild‐type thermostable AmDH from *Petrotoga mobilis* was undertaken by Cai and co‐workers.^[238]^ This enzyme naturally catalyses the amination of levulinic acid. The best performing variant was used to generate the optically pure (*S*)‐4‐aminopentanoic acid with >99 % *ee* and 90 % yield in the valorisation of lignin waste.

#### 2.2.3 Other biocatalysts for bioreductive amination

**Scheme 30:**
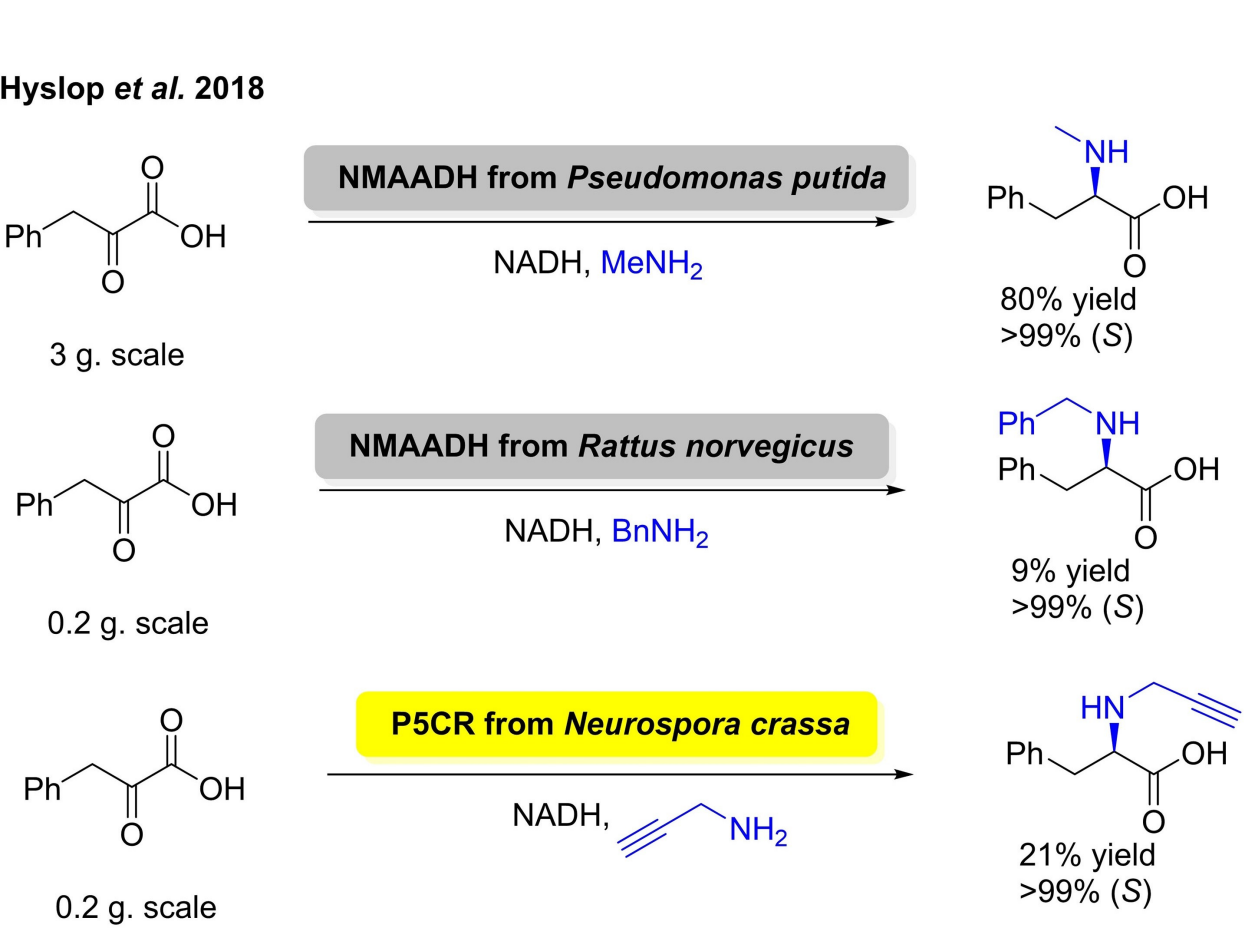
*N*‐Methyl amino acid dehydrogenases (NMAADHs/DpkAs), ketimine reductases (KIREDs), and pyrroline‐5‐carboxylate reductases (P5CRs) for the synthesis of *N*‐alkylated amino acids.

Besides IREDs, RedAms and AmDHs, other reductases have recently been found to be useful catalysts for bioreductive aminations. *N*‐Methyl amino acid dehydrogenases (NMAADHs/DpkAs), ketimine reductases (KIREDs), and pyrroline‐5‐carboxylate reductases (P5CRs) have been the focus of research by researchers at GSK, where various enzymes from these families were used to access a series of *N*‐alkylated amino acids (Scheme 30).^[239]^ Although NMAADHs, ketimine reductases and pyrroline‐5‐carboxylate reductases all perform similar reactions in nature, there present low sequence homology between each family. A number of biocatalysts belonging to these families were screened towards the reductive amination of phenylpyruvic acid derivatives with different amine partners. These enzymes were demonstrated to be useful catalysts for the synthesis of *N*‐functionalised amino acids, with complementary amine substrate scope and with some of these transformations carried out at a gram scale. Although activity was observed to dramatically decrease from 10 to 1 amine equiv., successful evolution campaigns on IREDs suggest that this limitation can be overcome soon. Finally, and in the same context, the DpkA from *Pseudomonas putida* was recombinantly expressed in *Corynebacterium glutamicum* to generate a range of *N*‐alkylated glycine derivatives.^[240]^

## 3 Novel Approaches on Biocatalytic C−O Bond Formation

The selective introduction of an oxygen atom into a non‐activated C−H bond represents a major challenge in organic chemistry.^[241]^ Besides cytochrome P450 monooxygenases, which have been extensively used for this task in the last two decades,^[242]^ recent research on peroxygenases,^[243]^ other monooxygenases,^[244]^ and hydratases^[245]^ has greatly expanded the range of biocatalysts for this purpose. In this survey, we have focused on new research conducted on two classes of enzymes which we envisage will be the focus of extensive studies in the coming years.

### 3.1 *O*‐Methyltransferases

**Scheme 31:**
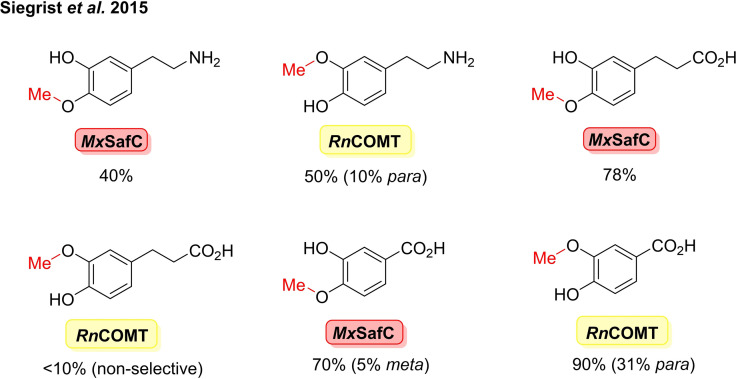
Substrate scope, conversions and regioselectivity displayed by the catechol *O*‐methyltransferases from *Myxococcus xanthus* (*Mx*SafC) and *Rattus norvegicus* (*Rn*COMT).

Methylation is an important and common structural modification in organic molecules especially in medicinal chemistry where methods for the selective introduction of methylene groups are popular in drug late‐stage modifications.^[246]^ Methyltransferases (MTs) are widespread in Nature and are found in a large variety of organisms across the different kingdoms.^[247]^ Their use in organic chemistry represents an attractive alternative to conventional approaches that often require the use of toxic compounds such as alkylhalides or sulfates, as well as highly reactive species such as Grignard reagents or organolithium compounds.^[248]^ Most methyltransferases employ *S*‐adenosyl‐L‐methionine (SAM) as methyl donor in which the methyl group is delivered from the sulfonium moiety present in the molecule. Traditionally, these enzymes have presented several limitations that have impeded the transition from lab scale experiments to chemical process. Firstly, MTs do not display the broad substrate scope other valuable enzymes have although we envisage that novel approaches on enzyme discovery as well as directed evolution campaigns targeting these enzymes will soon provide a broad range of MTs able to act on a wide variety of substrates. A key aspect on the viability of SAM‐dependent MTs is closely related to the development of efficient, cheap, and simple regeneration strategies to be used *in vitro* as SAM is expensive, rather unstable, and presents a low atom efficiency. Several strategies have been developed over the last decade which mainly consist in two approaches: a) *in situ* SAM formation through enzymatic cascades or b) catalytic cofactor regeneration.^[249]^ For instance, SAM can be supplied from ATP and L‐methionine employing a methionine adenosyltransferase. Following this approach, Andexer and co‐workers studied the regioselective methylation of several phenolic compounds^[250]^ using two regiocomplementary catechol *O*‐methyltransferases (COMTs) (Scheme 31). By using the COMT from *Rattus norvegicus* the corresponding methylated products of dopamine, dihydrocaffeic acid or 3,4‐dihydroxybenzoic acid in the *meta*‐position were obtained in conversions that ranged from excellent in the case of 3,4‐dihydroxybenzoic acid (90 % conversion, 2 : 1 regioselectivity ratio), moderate for dopamine (50 %, 4 : 1 ratio) and poor for dihydrocaffeic acid (<10 %). For the selective *para*‐methylation of such substrates, the *Mx*SafC from the saframycin biosynthetic pathway in *Myxococcus xanthus* was selected. This enzyme shows in most cases an excellent selectivity (>13 : 1) towards that position although this has been found to be substrate dependent, observing *meta*‐selectivity for 3,4‐dihydroxybenzoic acid. Conversions from 40 % for dopamine up to 80 % with both acids were obtained. An additional enzyme was required to deplete SAH as inhibition was observed, thus highlighting the importance of developing catalytic strategies.

Subsequently, in this context, the same group developed an approach in which only the substrate, L‐methionine, and polyphosphate are added into the reaction vessel as substrates.^[251]^ A linear cascade consisting of a three‐enzyme cascade to produce and regenerate ATP was firstly implemented on the systems abovementioned observing full conversion in both the *Rn*COMT‐mediated methylation of 3,4‐dihydroxybenzoic acid and *Mx*SafC‐catalysed methylation of dihydrocaffeic acid. A cyclic cascade was then developed by substituting the nucleosidase for a SAH hydrolase to generate adenine which would enter the cycle again to form ATP. Despite the efforts, a significant drop in turnover numbers was observed. Albeit very elegant, these complex systems still represent a major challenge in large‐scale synthesis as multi‐enzyme cascades are not always easy to efficiently combine in a one‐pot manner.

**Scheme 32:**
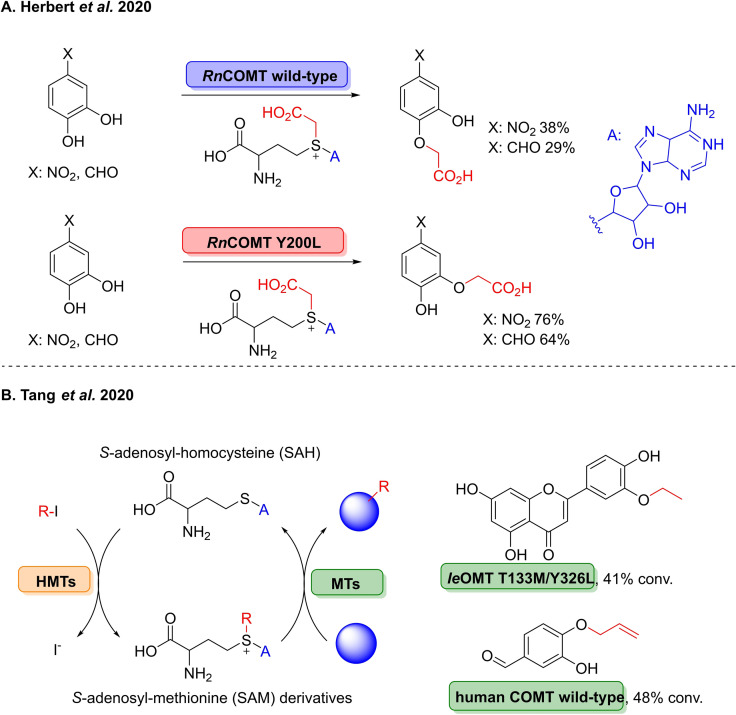
Novel strategies for general enzymatic *O*‐alkylation involving methyltransferases using (A) stoichiometric naturally occurring SAM derivatives and (B) in combination with halide methyltransferases to generate artificial SAM derivatives.

Recently, Seebeck *et al*. demonstrated that methyl halide transferases (HMTs) can be used to remethylate SAH by using methyl iodide.^[252]^ The authors tested the versatility of this system by combining it with different MTs for C, N, and O‐methylation, including the Inositol 4‐MT from *Mesembryanthemum crystallinum* (IMT) obtaining 4‐methyl inositol in 90 % conversion. The use of HMTs has not only allowed for a simpler regeneration system for SAM‐dependent methylations, but it has also opened the possibility to expand the scope towards general enzymatic alkylations using catalytic loadings of the cofactor, as HMTs can accept other alkyl groups from alkyl halides to produce different SAM analogues (Scheme 2). SAM analogues for alkylations had been previously developed using different chemical and enzymatic approaches[^247^, ^253^] and applied to the synthesis of alkylated and carboxymethylated analogues of rapamycin, rebeccamycin^[254]^ and phenolic compounds (Scheme 32A),^[255]^ although as previously mentioned, the need for stoichiometric amounts of these cofactors represents a major drawback for the large‐scale use of this strategy *in vitro*.

The use of HMTs for catalytic alkylation has been recently exploited by the Bornscheuer laboratory. They identified the HMT from *Arabidopsis thaliana* (*At*HMT) as the most promising catalyst to be engineered to broaden the scope of alkyl iodides to be efficiently accepted.^[256]^ A high‐throughput screening based on iodide detection was developed for the selection of improved variants. Libraries targeting residues in the active site were generated and screened for ethyl iodide acceptance, finding a variant V140T that showed a 6‐fold improvement in *k_cat_* over that of the wild‐type. The system was applied to the ethylation of luteolin using the isoeugenol MT (*Ie*OMT) double variant T133M/Y326L and the allylation of 3,4‐dihydroxybenzaldehyde using human COMT obtaining the corresponding products in 41 % and 48 % conversions, respectively (Scheme 32B).

Very recently, the Seebeck laboratory have also demonstrated that the HMT from *Burkholderia xenorovans* can produce *S*‐adenosyl‐(fluoromethyl)‐L‐homocysteine (F‐SAM) from fluoromethyliodide and SAM and hence employed in fluoromethylation reactions.^[257]^ Several methyltransferases were assayed using this system, including the trans‐aconitate 3‐methyltransferase from *S. cerevisiae* (TAMT) for the enzymatic fluoromethylation of trans‐aconitate and 3‐isopropylmalate, observing the formation of the corresponding fluororomethylated esters in excellent conversions.

Previous research by Mickefield *et al*. demonstrated that scope and regioselectivity can be tunned^[258]^ so further studies on MTs engineering as well as the implementation of these newly developed systems for cofactor regeneration and general alkylation can greatly expand and make these systems readily available for the use in general synthetic chemistry.

Alternatively, cobalamin‐dependent MTs have also emerged as a valuable tool for *O*‐methylation. Their scope to date is very similar to that of SAM‐dependent MTs although, unlike SAM‐dependent enzymes which can only catalyse the methylation/alkylation reactions, cobalamin‐dependent MTs can also be employed to perform the cleavage of C−O bonds and have been recently used as a complementary tool to P450s for enzymatic demethylation.[^259^, ^260^] Methylation of phenols is a common strategy in organic synthesis to protect the phenol moiety and perform further chemistry. Subsequent deprotection often involves the use of strong acids such as HBr or hazardous reagents such as BBr_3_ so alternative strategies to perform this process under mild conditions are advantageous. The use of cytochrome P450 for regioselective demethylation has been the focus of research by the Flitsch and Bornscheuer groups.[^261^, ^262^, ^263^] This process is NADPH dependent and proceeds through methyl group hydroxylation followed by decomposition of this unstable species.

**Scheme 33:**
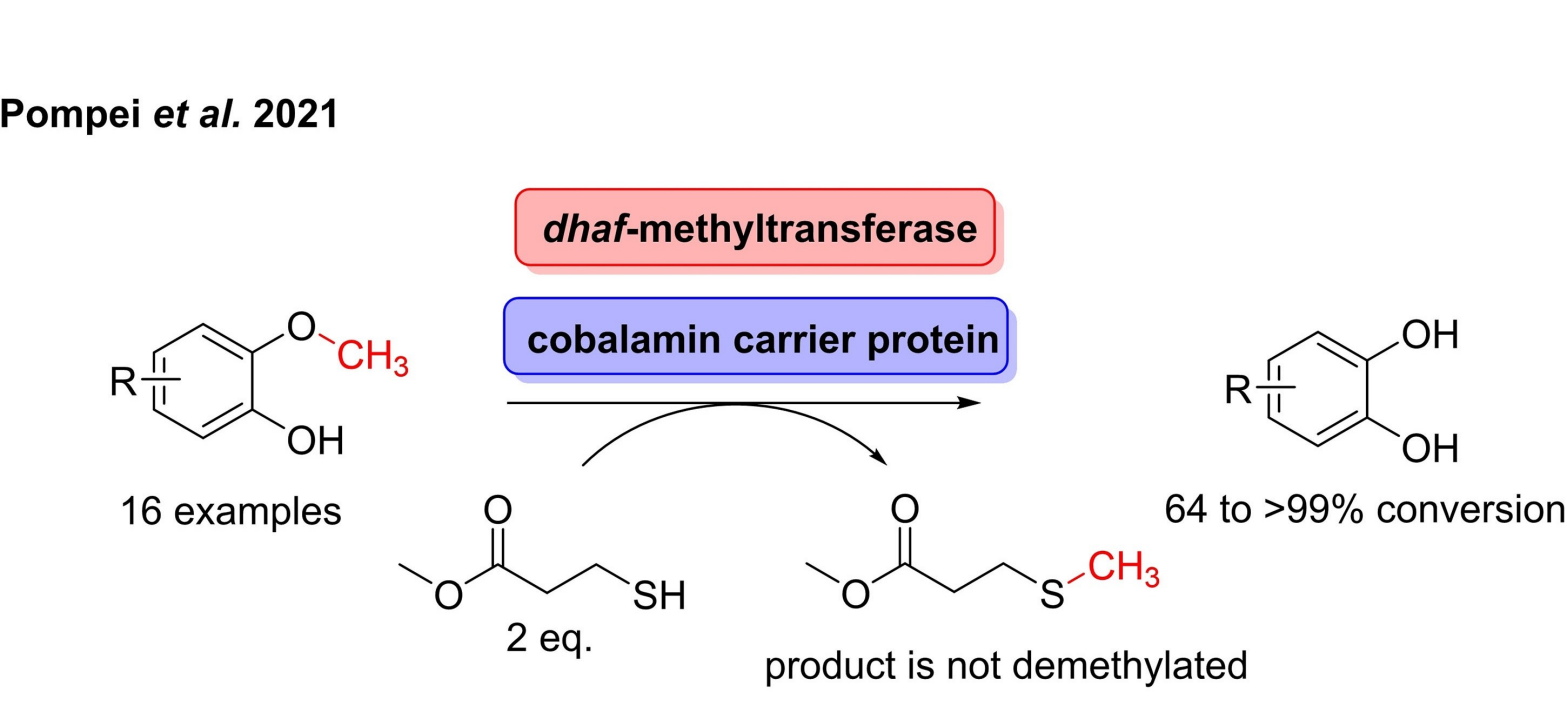
Enzymatic demethylation employing cobalamin‐dependent methyltransferases using thiols as methyl acceptors.

Extensive research in this aspect has recently been done by Kroutil and co‐workers. In 2018 they explored the use of a two‐enzyme system for the methylation/demethylation of a series of phenyl methyl esters.^[264]^ This system consisted in the combination of a methyl transferase from *D. hafniense* which, working together with a corrinoid protein as methyl group shuttle, demethylates one substrate and methylates a second one. In this manner, the demethylation of a series of methyl phenyl ethers was achieved in conversions up to 82 %. However, the need for a large excess of the methyl group acceptor to drive the equilibrium towards the formation of the desired product as well as side products observed due to isomerisation^[265]^ represent a limitation for large scale applications. Very recently, the same authors have reported the use of thiols as methyl acceptors.^[266]^ The use of these compounds represents a step forward towards the applicability of these systems in synthetic chemistry as they cannot be demethylated, therefore a large excess of the acceptor is no longer needed to drive the equilibrium. Using the enzymatic demethylation of guaiacol as the model system, different thiols were screened, finding mercaptopropionic acid ethyl ester as the best methyl acceptor. A series of substituted guiacols were tested for demethylation using 2 equivalents of acceptor observing conversions that ranged from 64 to >99 %, with isomerisation detected in some cases as a side reaction (Scheme 33).

Finally, another interesting class of SAM‐dependent MTs to be exploited are carboxyl methyltransferases (CMTs). They catalyse the methylation of the hydroxyl group in a carboxylic acid, yielding the corresponding methyl ester. A recent review by Carnell and co‐workers covers this subclass of methyltransferases comprehensively.^[267]^ Several potential applications of CMTs have being identified such as biodiesel production and in bio‐based materials. However, challenges involving the use and regeneration of SAM, as well as the fact that other suitable enzymatic strategies (such as lipases) are well‐established, limit their potential applicability in industrial biocatalysis. However, we envisage that this class of enzymes can be a valuable tool in medicinal chemistry in late‐stage functionalisation due to their selectivity and the mild reactions conditions they operate under.

### 3.2 Fe and 2‐oxoglutarate dependent oxygenases

A particularly attractive alternative to other enzymatic strategies for the insertion of hydroxyl groups into non‐activated C−H bonds are iron and α‐ketoglutarate dependent oxygenases (Fe/αKGs). These enzymes present some potential advantages for industrial biocatalysis compared to other well studied classes of enzymes such as P450 monooxygenases. For instance, αKG and oxygen are cheaper compared to nicotinamide cofactors, and they do not present the H_2_O_2_ inhibition issues observed in peroxygenases. Moreover, these enzymes are widespread in Nature and are involved in the selective hydroxylation of a broad range of compounds, including small molecules and amino acids in high regio‐ and stereoselectivity.^[268]^ To date, most Fe/αKGs have been applied to the selective hydroxylation of amino acids and given that these compounds have been extensively used by chemists as chiral pools in total synthesis, Fe/αKGs have been successfully implemented in chemoenzymatic approaches to natural products. A comprehensive and recent review by Renata and co‐workers covers these studies.^[269]^ In this section we will focus on amino acid Fe/αKGs, which we believe have a larger potential for small chiral molecule synthesis and therefore applications in different industries.

**Figure 3:**
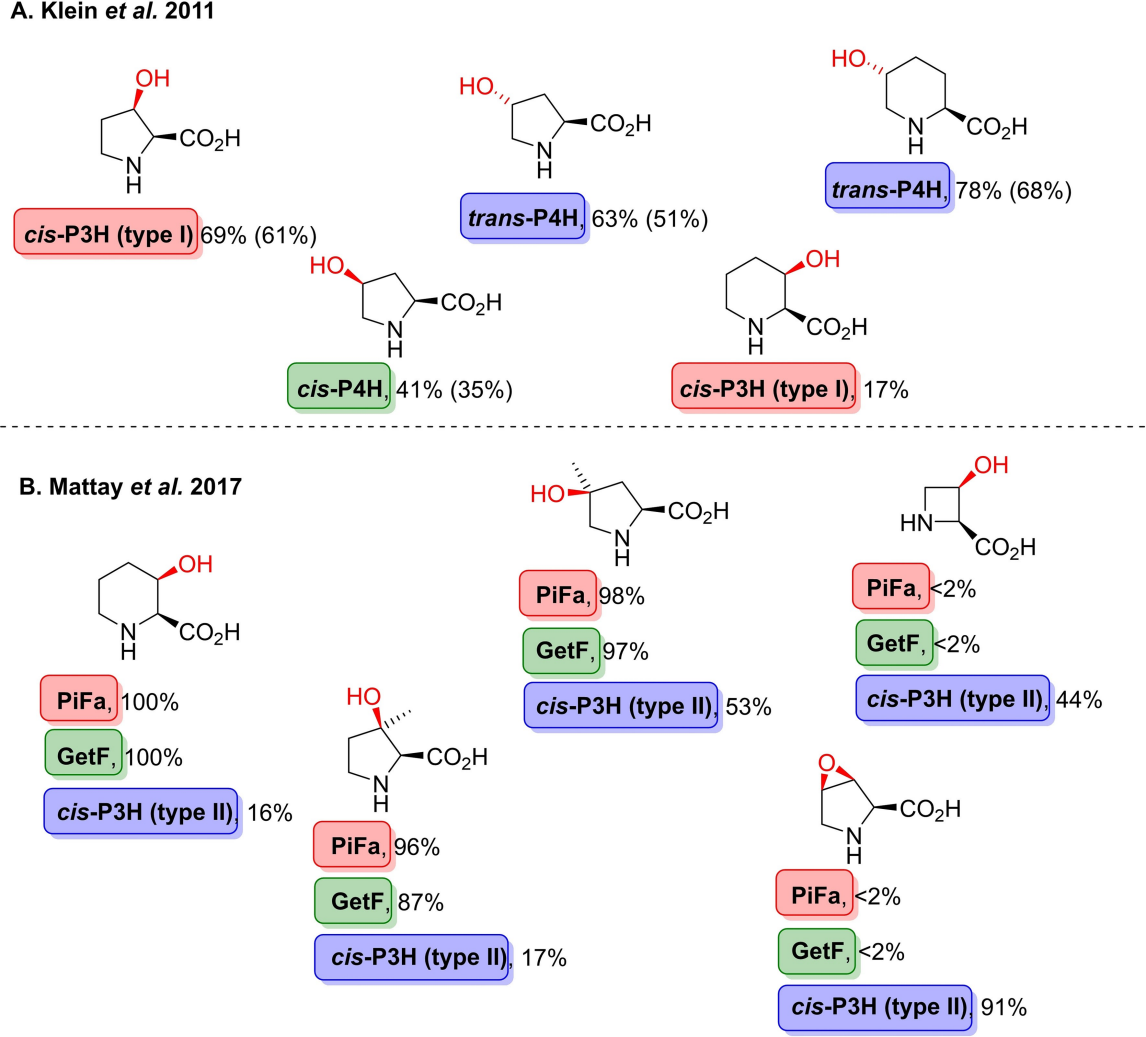
(A) Proline hydroxylases for the synthesis of different proline and pipecolic acid hydroxylated derivatives. Yields for selected biotransformations at up to 500 mg scale are in brackets. (B) Pipecolic acid hydroxylases and substrate scope. Relative conversions normalised to their natural substrate (PiFa and GetF: pipecolic acid; *cis*‐P3H: proline).

For instance, proline hydroxylases catalyse the regio‐ and stereoselective hydroxylation of proline and several enzymes have now been identified and heterologously expressed to access all possible stereoisomers.[^270^, ^271^] Hydroxyprolines are important building blocks in pharmaceutical and fine chemical industries as this core can be found in different active pharmaceutical ingredients, and proline derivatives are powerful tools that are extensively used in organocatalysis. In 2011, Klein *et al*. described the production and applications of four proline hydroxylases, termed *cis*‐P3H, *cis*‐P4H, *trans*‐P3H, and *trans*‐P4H according to the major stereoisomer obtained (Figure 3A).^[271]^ A thorough characterisation was carried out and reactions up to a 500 mg scale performed obtaining the corresponding products in moderate to good yields (35–68 %).

More recently, a clade within this family was found to be active on pipecolic acid and some proline derivatives but no proline itself, which prompted the authors to name this subgroup of enzymes pipecolic acid hydroxylases (PiHs) (Figure 3B).^[272]^ Three of them, GetF, PiFa, and *cis*‐P3H (type II) were characterised, and their substrate scope mapped. Remarkably, not only different C‐methylated proline derivatives yielding the corresponding tertiary alcohols can now be accessed, but also 3,4‐dehydroproline and L‐azetidine‐2‐carboxylic acid can be hydroxylated leading to highly functionalised small molecules. The possibility to access all possible stereoisomers as well as the ability to hydroxylate other cyclic amino acids made this group of enzymes an attractive tool for early‐stage modifications.

**Scheme 34:**
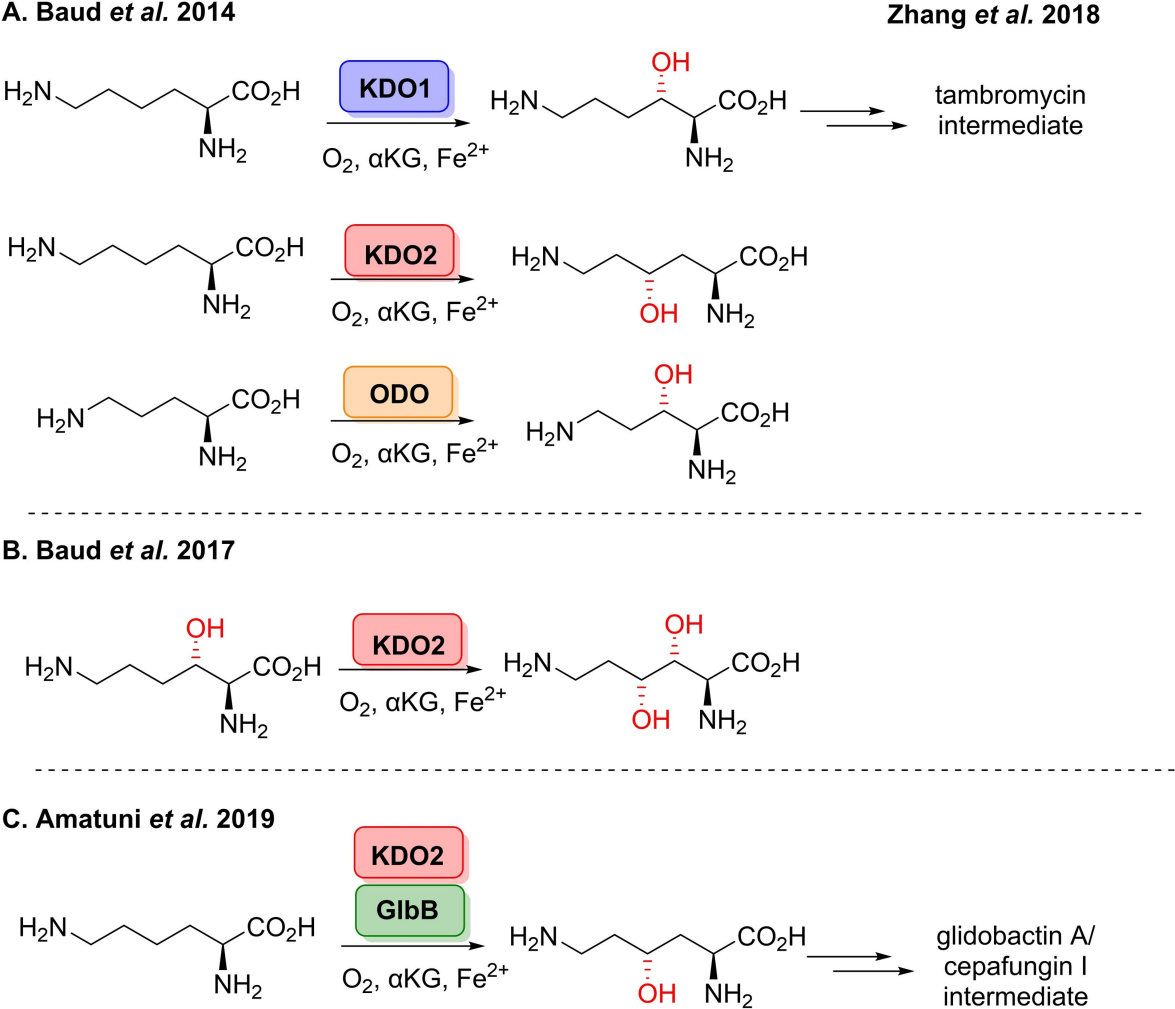
Lysine and ornithine hydroxylases and their synthetic utility towards the preparation of amino alcohols and valuable intermediates in the total synthesis of natural products.

In 2014, Zaparucha and co‐workers discovered several enzymes able to hydroxylate the side chain of basic amino acids such as lysine or ornithine through a genome mining approach.^[273]^ These enzymes – named KDO1‐5 and ODO – showed complementary regioselectivity and substrate acceptance (Scheme 34A), and in a follow‐up report they also demonstrated that they can be sequentially combined to access highly functionalised amino alcohols (Scheme 34B).^[274]^ The synthetic utility of these family of enzymes has been recently proved by the Renata lab by using KDO1 to hydroxylate L‐lysine at a gram scale in full conversion that was subsequently transformed to produce an intermediate towards the synthesis of the natural product tambromycin.^[275]^ The same group has recently identified a new lysine hydroxylase showing a preference for γ‐hydroxylation, GlbB, which despite showing a narrow substrate scope, demonstrated its synthetic utility in the synthesis of a fragment of glidobactin (Scheme 34C).^[276]^

**Scheme 35:**
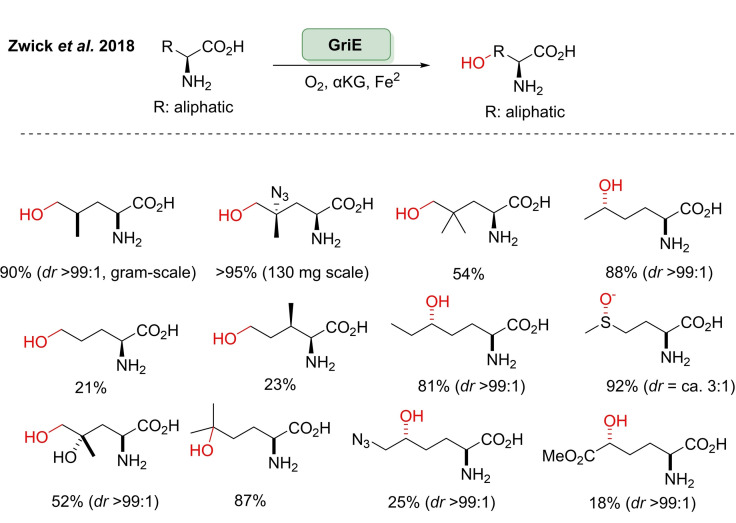
Substrate tolerance of the leucine hydroxylase GriE. Yields and *d.r*. of biotransformations performed at a 0.02 mmol scale when not specified.

Concerning acyclic aliphatic amino acids, Müller and co‐workers in 2017 discovered a Fe/αKG oxygenase encoded in the gene cluster involved in the biosynthesis of 4‐methyl‐proline, in which the first step comprises the terminal hydroxylation of L‐leucine.^[277]^ A crystal structure of this new leucine hydroxylase ‐ named GriE ‐ was obtained that gave insight into the residues involved in the reaction that can lead to future structure‐guided engineering. The synthetic potential of this enzyme was recently exploited by Zwick *et al*. A study of the substrate scope was performed showing that this enzyme possesses a broad substrate tolerance towards the hydroxylation of branched and linear aliphatic amino acids (Scheme 35), with some of these reactions performed at a gram‐scale and used in the total synthesis of alkaloids.^[278]^

Due to the occurrence of these enzymes, the broad scope some of them display, and the advantages in terms of process intensification they have compared to other biocatalysts with similar reactivity, we envisage they can become a powerful tool in small molecule synthesis in the coming decade.

## Conflict of interest

The authors declare no conflict of interest.

## Biographical Information

*Jack Sangster received his M.Sc. in Chemistry from Heriot‐Watt University (Edinburgh) where he completed his research project on molecular biosensors for oesophageal cancer detection under the supervision of Dr. Nicola Howarth. He is currently studying for his PhD at the University of Manchester under the supervision of Professor Nicholas Turner. His research focuses on enantioselective chemoenzymatic cascades for C−C bond formation*.

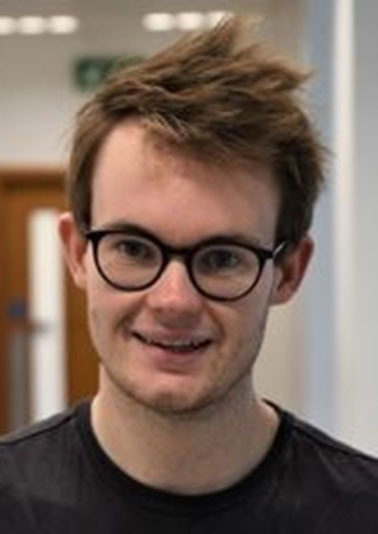

## Biographical Information

*James Marshall obtained his bachelors in Biochemistry from Newcastle University in 2017 and completed his Ph.D. at the University of Manchester in the Manchester Institute of Biotechnology (MIB) under the supervision of Professor Nicholas Turner. He worked for a year at the enzyme discovery and production company Prozomix Ltd. in Haltwhistle Northumberland and then joined the MIB in 2017 where his research focused on a metagenomics approach to discover novel biocatalysts for chiral amine synthesis, namely in the area of imine reductases. He now works at Unilever Research & Development, Port Sunlight*.

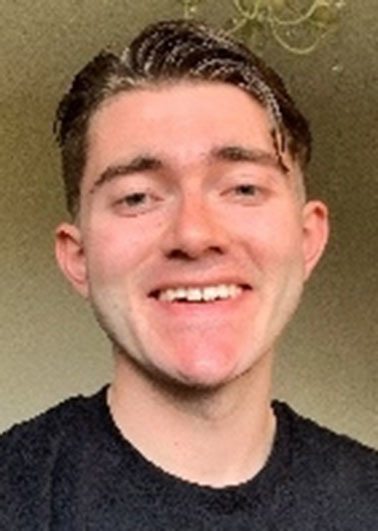

## Biographical Information

*Nick Turner obtained his DPhil in 1985 with Professor Sir Jack Baldwin and from 1985–1987 was a Royal Society Junior Research Fellow, spending time at Harvard University with Professor George Whitesides. He was appointed lecturer in 1987 at Exeter University and moved to Edinburgh in 1995, initially as a reader and subsequently professor in 1998. In October 2004 he joined Manchester University as professor of chemical biology in the Manchester Institute of Biotechnology. He is director of the Centre of Excellence in Biocatalysis (CoEBio3) and a co‐director of SYNBIOCHEM, the BBSRC Synthetic Biology Research Centre. He is also a co‐founder of Ingenza (* www.ingenza.com *). His research interests are in the area of biocatalysis with particular emphasis on the discovery and development of novel enzyme catalysed reactions for applications in organic synthesis. His group is also interested in the application of directed evolution technologies for the development of biocatalysts with tailored functions*.

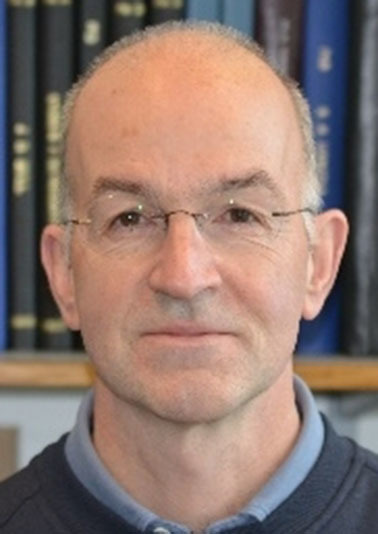

## Biographical Information

*Juan Mangas obtained his Ph.D. at the University of Oviedo (Spain) under the supervision of Vicente Gotor‐Fernandez working on new biocatalytic routes to optically active alcohols. He then moved to Lund University in Sweden to work in Professor Patrick Adlercreutz's group on the optimisation of chemoenzymatic processes to obtain biodiesel, tailored triglycerides and prebiotics using hydrolases. In 2015, he joined the group of Professor Nicholas Turner as a research associate at the Manchester Institute of Biotechnology to work on the discovery, engineering, characterisation, and applications of novel biocatalysts to produce chiral amines. He has recently been recruited by the Aragonese Foundation for Research & Development (ARAID) and now works as a senior scientist at the Institute of Chemical Synthesis and Homogeneous Catalysis (ISCQH‐CSIC) in Zaragoza. He is interested in the development of sustainable synthetic routes to high value chemicals through chemoenzymatic cascades, enzyme evolution, and artificial metalloenzymes*.